# Effect of Treatment Expectation on Placebo Response and Analgesic Efficacy: A Secondary Aim in a Randomized Clinical Trial

**DOI:** 10.1001/jamanetworkopen.2020.2907

**Published:** 2020-04-16

**Authors:** Anne E. Sanders, Gary D. Slade, Roger B. Fillingim, Richard Ohrbach, Samuel J. Arbes, Inna E. Tchivileva

**Affiliations:** 1 Division of Pediatric and Public Health, Adams School of Dentistry, University of North Carolina at Chapel Hill; 2 Department of Community Dentistry and Behavioral Science, University of Florida, Gainesville; 3 Department of Oral and Maxillofacial Surgery, University at Buffalo, State University of New York, Buffalo; 4 Rho Inc, Durham, North Carolina; 5 Division of Oral and Craniofacial Health Sciences, Adams School of Dentistry, University of North Carolina at Chapel Hill

## Abstract

This randomized clinical trial assessed the effect of patients’ treatment expectations on the efficacy of propranolol vs placebo among patients with temporomandibular disorder–associated myalgia.

## Introduction

Amid the United States’ chronic pain crisis, novel analgesics are failing to show efficacy in clinical trials.^1^ High failure rates are attributed to an upward trend in placebo response,^2^ driven by patients’ heightened expectation of treatment benefit.^3^ We hypothesized that heightened expectations differentially amplify placebo analgesia, leading to underestimation of the treatment effect in randomized clinical trials.

## Methods

SOPPRANO (Study of Orofacial Pain and Propranolol) is a double-blind, placebo-controlled, parallel-group, phase 2b randomized clinical trial that enrolled 200 adults aged 18 to 65 years with examiner-verified temporomandibular disorder–associated myalgia from August 1, 2015, to January 31, 2018, at 3 US study sites. Participants were randomized 1:1 to propranolol hydrochloride (60 mg twice a day) or placebo administered for 9 weeks. Using daily pain diaries, treatment response was defined as the proportion with at least 30% reduction in mean pain index (facial pain intensity multiplied by duration) at 9 weeks. Efficacy was further quantified as the number needed to treat (NNT) with 95% CIs. Treatment expectation was determined from participants’ baseline expectation that the study treatment would reduce their facial pain. Ratings of moderate or strong were classified as high treatment expectation, and ratings of none or slight were classified as low expectation. In this planned, intention-to-treat analysis, we tested whether treatment expectation modified the analgesic efficacy of propranolol using a log binomial generalized estimating equation regression model incorporating data from 4 study visits with adjustment for covariates. The generalized score statistic for generalized estimating equation models was used to test for modification of analgesic efficacy. A 2-tailed *P* < .05 was deemed statistically significant, and values of .05 to .10 were deemed credible. Other results were deemed statistically significant when 2-tailed 95% CIs excluded the null value. A separate logistic regression model assessed the odds of high treatment expectation using continuous measures of clinical and experimental pain, psychological factors, and health-related quality of life at baseline, all standardized to *z* scores.

SOPPRANO’s study flow diagram and checklist are not yet published.^4^ The trial protocol and statistical plan are available in Supplement 1. This study followed the Consolidated Standards of Reporting Trials (CONSORT) reporting guidelines, and the study flow diagram is available in the eFigure in Supplement 2. The institutional review boards at each site approved the trial protocol. All participants provided written informed consent.

## Results

Among 198 participants in the intention-to-treat sample who rated their treatment expectation, the mean (SD) age was 34 (0.90) years, 155 (78.3%) were women, and 118 (59.6%) had a high expectation of pain relief. Pressure pain sensitivity in the masseter muscle (odds ratio, 1.5; 95% CI, 1.1-2.0) and trapezius muscle (odds ratio, 1.5; 95% CI, 1.1-2.0) was associated with a heightened expectation of pain relief (Table). Among participants with low expectation of pain relief (Figure, A), treatment responders composed 73.5% in the propranolol group and 42.7% in the placebo group. This difference corresponded to an NNT of 3.2 (95% CI, 1.9-11.8; *P* = .007). In the high-expectation stratum (Figure, B), treatment responders composed 67.0% and 63.6% in propranolol and placebo groups, respectively. The heightened response in the placebo group nullified efficacy (NNT = 29.6; 95% CI, 4.4 to −6.3; *P* = .73). The *P* value for this interaction was .07.

**Table.** Unadjusted Associations Between Baseline Participant Characteristics and High Expectation of Treatment Benefit

Baseline characteristic	Participants, No.^**a**^	Standardized odds ratio (95% CI)^b^
Age, y (range, 18-64 y)	198	1.4 (1.0-1.9)
Sex^c^		
Male	43	1.0 (0.5-2.1)
Female	155	1 [Reference]
Race/ethnicity^c^		
White	154	0.8 (0.4-1.6)
Other	44	1 [Reference]
Study group^c^		
Propranolol	100	1.6 (0.9-2.8)
Placebo	98	1 [Reference]
Coping Strategies Questionnaire, subscale		
Distraction	198	1.0 (0.8-1.4)
Catastrophizing	198	1.1 (0.8-1.4)
Ignoring pain	196	1.0 (0.8-1.4)
Distancing	198	0.9 (0.7-1.3)
Coping	195	0.9 (0.7-1.2)
Praying	197	1.6 (1.2-2.2)
Graded Chronic Pain Scale		
Facial pain intensity	198	1.3 (0.9-1.7)
Facial pain interference	198	1.3 (1.0-1.8)
Psychosocial factors		
HADS Anxiety	197	1.1 (0.8-1.4)
HADS Depression	196	1.1 (0.8-1.5)
Perceived Stress Scale	197	1.1 (0.9-1.5)
Symptom Checklist 90R Somatization	195	1.3 (0.9-1.7)
Physical and mental functioning		
Headache Impact Test-6	194	1.0 (0.8-1.4)
Jaw Functional Limitation Scale	195	1.1 (0.8-1.4)
SF-12v2 physical composite score	192	0.8 (0.6-1.0)
SF-12v2 mental composite score	192	1.0 (0.8-1.4)
Vital signs		
Heart rate	198	1.1 (0.8-1.4)
Systolic blood pressure	198	0.9 (0.7-1.3)
Diastolic blood pressure	198	1.2 (0.9-1.6)
Experimental pressure pain thresholds^d^		
Temporalis muscle	198	1.3 (1.0-1.7)
Masseter muscle	198	1.5 (1.1-2.0)
Temporomandibular joint	198	1.2 (0.9-1.6)
Trapezius muscle	197	1.5 (1.1-2.0)
Lateral epicondyle	196	1.3 (1.0-1.7)

Abbreviations: HADS, Hospital Anxiety and Depression Scale; SF-12v2, Short-Form 12 Health Survey, version 2.

^a^ Data are missing for variables with fewer than 198 observations.

^b^ Standardized odds ratios from a binary logistic regression model are interpreted as the increase in odds of high treatment expectation per standard deviation increase in baseline characteristic.

^c^ Sex, race/ethnicity (self-reported), and treatment group are binary variables and therefore were not standardized.

^d^ Mean pressure pain threshold values are reverse scored so that higher values denote greater sensitivity to experimental pain.

**Figure.**
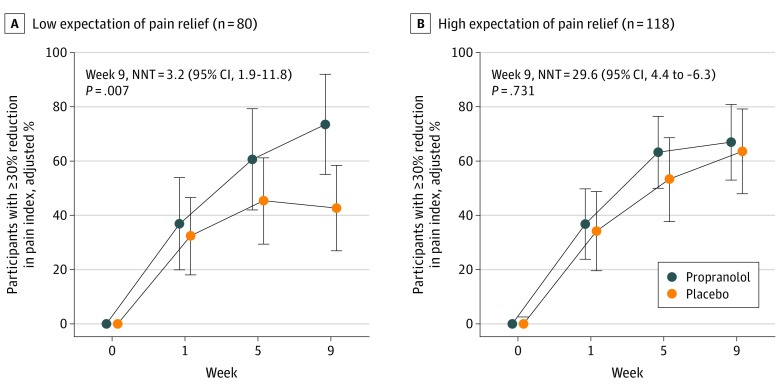
Propranolol and Temporomandibular Disorder in Low and High Strata of Treatment Expectation Analgesic efficacy of propranolol (60 mg twice a day) on facial pain in participants with temporomandibular disorder myalgia was modified by baseline expectations of pain relief (*P* = .07 for interaction at week 9 of treatment expectation and treatment group). A, For 80 participants with low treatment expectation, the placebo response was low (decreasing between the third and fourth visits), propranolol was efficacious, and the number needed to treat (NNT) was 3.2. B, For 118 participants with high treatment expectation, placebo response increased at each subsequent visit, and no therapeutic effect of propranolol was evident. Adjusted percentages and their 95% confidence intervals (error bars) were estimated with a log binomial generalized estimating equation regression model allowing for repeated visits by study participants, with adjustment for study site, sex, and self-reported race. *P* < .05 was the threshold for statistical significance.

## Discussion

In this study, propranolol was superior to placebo, but only among participants whose expectations of treatment were modest. In the presence of heightened expectations, placebo analgesia overwhelmed the efficacy signal, inflated the NNT, and nullified differences between treatment groups. The relatively small sample size limited the power to test if the magnitude of effect of propranolol on temporomandibular disorder–associated pain differed by treatment expectation, yet our findings offer credible evidence of interaction. Few clinical or psychological factors were associated with heightened expectation, with sensitivity to experimental pressure pain being the exception. Greater pain sensitivity may manifest as greater need for and expectation of pain relief. It is well established that treatment expectations are susceptible to verbal suggestion, physician manner, and social observation.^5^ A more recent influence may be information relayed through direct-to-consumer advertising of prescription drugs. Direct-to-consumer advertising budgets for prescription drugs more than quadrupled in the United States from 1997 to 2016,^6^ coinciding with the steady increase in placebo response. We recommend assessing treatment expectation to better understand its potential bias on success rates in clinical trials.

## Notes

Supplement 1. **Trial Protocol**

Supplement 2. **eFigure. **CONSORT Flow Diagram

Supplement 3. **Data Sharing Statement**
