# Supplementary material for: Effect of Treatment Expectation on Placebo Response and Analgesic Efficacy: A Secondary Aim in a Randomized Clinical Trial
Source: JAMA Netw Open. 2020 Apr 16;3(4):e202907. doi: 10.1001/jamanetworkopen.2020.2907 (PMC7163405; doi:10.1001/jamanetworkopen.2020.2907)
Supplement: Supplement 1. — Trial Protocol [file jamanetwopen-3-e202907-s001.pdf]

Effect of *COMT* Genetic Polymorphisms on Response to Propranolol Therapy in Temporomandibular Disorder

NIDCR Protocol Number: 14-067-E

NIDCR Funding Mechanism: DE024169-01

Principal Investigator: Inna Tchivileva, MD

NIDCR Program Official: Gallya Gannot, DMD, PhD

Version Number: 12.0

12 Feb 2018

STATEMENT OF COMPLIANCE

The study will be carried out in accordance with Good Clinical Practice (GCP) as required by the following:

- United States (US) Code of Federal Regulations (CFR) applicable to clinical studies (45 CFR Part 46, 21 CFR Part 50, 21 CFR Part 56, and 21 CFR Part 312)
- International Conference on Harmonisation (ICH) E6
- National Institutes of Health (NIH) Clinical Terms of Award

All key personnel have completed Human Subjects Protection Training.

SIGNATURE PAGE: UNIVERSITY OF NORTH CAROLINA

The signature below constitutes the approval of this protocol and the attachments and provides the necessary assurances that this trial will be conducted according to all stipulations of the protocol, including all statements regarding confidentiality, and according to local legal and regulatory requirements and applicable US federal regulations and ICH guidelines. The protocol should be signed by the clinical site investigator who is responsible for the day-to-day study implementation at his/her specific clinical site.

Site Investigator:

Signed: ______________________________ Date: ______________

Inna Tchivileva, MD
Research Assistant Professor
University of North Carolina School of Dentistry

SIGNATURE PAGE: UNIVERSITY OF FLORIDA

The signature below constitutes the approval of this protocol and the attachments and provides the necessary assurances that this trial will be conducted according to all stipulations of the protocol, including all statements regarding confidentiality, and according to local legal and regulatory requirements and applicable US federal regulations and ICH guidelines. The protocol should be signed by the clinical site investigator who is responsible for the day-to-day study implementation at his/her specific clinical site.

Site Investigator:

Signed: ______________________________ Date: ________________

Roger Fillingim, PhD
Professor
University of Florida College of Dentistry

SIGNATURE PAGE: UNIVERSITY AT BUFFALO

The signature below constitutes the approval of this protocol and the attachments and provides the necessary assurances that this trial will be conducted according to all stipulations of the protocol, including all statements regarding confidentiality, and according to local legal and regulatory requirements and applicable US federal regulations and ICH guidelines. The protocol should be signed by the clinical site investigator who is responsible for the day-to-day study implementation at his/her specific clinical site.

Site Investigator:

Signed: ______________________________________ Date: ________________

Richard Ohrbach, DDS, PhD
Associate Professor
University at Buffalo School of Dental Medicine

TABLE OF CONTENTS

Page

Statement of Compliance ii
Signature Page: University of North Carolina iii
Signature Page: University of Florida iv
Signature Page: University at Buffalo v
Table of Contents vi
List of Abbreviations x
Protocol Summary xii
1 Key Roles 17
2 Introduction: Background Information and Scientific Rationale 21
2.1 Background Information 21
2.1.1 Background on Temporomandibular Disorder 21
2.1.2 Current Pharmacologic Management of TMD 21
2.1.3 Adrenergic System in Pathophysiology of TMD 21
2.1.4 Background on Propranolol 22
2.1.5 Background on Catechol-O-Methyltransferase as a Genetic Marker of Response to Propranolol 22
2.2 Rationale 23
2.3 Potential Risks and Benefits 24
2.3.1 Potential Risks 24
2.3.2 Known Potential Benefits 25
2.4 Study Objectives 25
2.4.1 Primary Objective 25
2.4.2 Secondary Objectives 25
2.4.3 Exploratory Objectives 26
2.5 Study Outcome Measures 26
2.5.1 Primary Outcome Measures 26
2.5.2 Secondary Outcome Measures 26
3 Study Design Overview 27
4 Study Enrollment and Withdrawal 28
4.1 Participant Inclusion Criteria 28
4.2 Participant Exclusion Criteria 29
4.3 Strategies for Recruitment and Retention 30
4.4 Treatment Assignment Procedures 31
4.4.1 Randomization Procedures 31
4.4.2 Blinding Procedures 31
4.4.3 Unblinding Procedures 31
4.4.4 Reasons for Participant Withdrawal 32
4.4.5 Handling of Withdrawals and Discontinuation 32
4.4.6 Suspension or Termination of the Study 33

5 Study investigational Product and Placebo....33
5.1 Investigational Product....33
5.1.1 Acquisition....33
5.1.2 Formulation, Packaging, and Labeling....33
5.1.3 Product Storage and Stability....34
5.2 Dosage and Administration of Investigational Product....34
5.3 Modification of Dosage of Investigational Product....34
5.4 Placebo....34
5.5 Accountability Procedures....35
5.6 Assessment of Participant Compliance....35
5.7 Concomitant Medications and Therapies....35
5.7.1 Rescue Medication....35
5.7.2 Nonpharmacological Therapy....36
5.8 Protocol Deviations Related to the Use of Investigational Product and Concomitant Therapies....36
6 Study Schedule....36
6.1 Prescreening (Day -49 to Day-7)....37
6.2 Screening and Baseline Visit 0 (Day -21 to Day-7)....37
6.3 Treatment (Visits 1-4)....38
6.3.1 Randomization and Titration Visit 1 (Day 0)....38
6.3.2 Maintenance Visit 2 (Day 7 [+ 3 days])....39
6.3.3 Maintenance Visit 3 (Day 35 [± 7 days])....39
6.3.4 Tapering Visit 4 (Day 63 [+6 days])....40
6.4 Follow-up Visit 5 (Day 77 [+ 7 days])....41
6.5 Early Termination Visit....41
6.6 Unscheduled Visit....41
7 Study Procedures....42
7.1 Clinical Evaluations....42
7.1.1 Medical History....42
7.1.2 Vital Signs and Physical Measurements....42
7.1.3 Pregnancy Test....42
7.1.4 Blood Draw....42
7.1.5 Electrocardiogram....42
7.1.6 Temporomandibular Disorder Examination....43
7.1.7 Tests for Heat Sensitivity....43
7.1.8 Pressure Pain Threshold Measurements....43
7.1.9 Manual Tender Point Examination....43
7.1.10 Daily Symptom Diary....44
7.1.11 The Alcohol Use Disorders Identification Test....45
7.1.12 Smoking Questionnaire....45
7.1.13 Symptom Inventory....45
7.1.14 Outcome Measure Questionnaires....45
7.2 Laboratory Evaluations....47

7.2.1 Clinical Laboratory Evaluations 47
7.2.2 Research Laboratory Evaluations....47
7.2.3 Specimen Preparation, Handling, and Shipping 47
8 Assessment of Safety....47
8.1 Specification of Safety Parameters 47
8.1.1 Unanticipated Problems 48
8.1.2 Adverse Events 48
8.1.3 Serious Adverse Events 49
8.2 Time Period and Frequency for Event Assessment and Follow-Up....49
8.3 Characteristics of Adverse Events 49
8.3.1 Relationship to Study Drug....49
8.3.2 Relationship to Study Procedures 50
8.3.3 Expectedness of AEs 50
8.3.4 Severity of Event 50
8.4 Reporting Procedures....50
8.4.1 Adverse Event Reporting to IRB and NIDCR....50
8.4.2 Unanticipated Problem Reporting to IRB and NIDCR 51
8.4.3 Serious Adverse Event Reporting to NIDCR....51
8.4.4 Reporting Abnormal Clinical Findings....52
8.4.5 Reporting of Pregnancy....52
8.5 Halting Rules 53
9 Study Oversight....53
10 Clinical Monitoring 53
11 Statistical Considerations 54
11.1 Study Hypotheses....54
11.1.1 Primary Hypothesis 54
11.1.2 Secondary Hypothesis 54
11.1.3 Exploratory Hypotheses 55
11.2 Sample Size Considerations....55
11.3 Analysis Samples 56
11.3.1 Intention-To-Treat Sample....56
11.3.2 Per-Protocol Sample 56
11.3.3 Safety Sample....56
11.4 Analyses....56
11.4.1 Analysis of the Primary Hypothesis 56
11.4.2 Analysis of the Secondary Hypotheses 57
11.4.3 Analysis of the Exploratory Hypotheses 58
11.5 Planned Interim Analyses 58
12 Source Documents and Access to Source Data and Documents 59
13 Quality Control and Quality Assurance 59
14 Protection of Human Participants 60
14.1 Institutional Review Board 60
14.2 Informed Consent Process 60

14.3 Exclusion of Special Populations 60
14.4 Participant Confidentiality 60
14.5 Future Use of Stored Specimens 61
14.6 Data Handling and Record Keeping 61
14.7 Data Management Responsibilities 61
14.8 Data Capture Methods 62
14.9 Types of Data 62
14.10 Timing of Reports 62
14.11 Study Records Retention 62
14.12 Protocol Deviations 62
15 Publication/Data sharing Policy 63
16 Literature References 64
Appendix A: Schedule of Events 68
Appendix B: Package Insert for Propranolol Hydrochloride extended-release 71

LIST OF ABBREVIATIONS

ACR	American College of Rheumatology
ADRB2	Adrenergic receptor β2
ADRB3	Adrenergic receptor β3
AE	Adverse event
APS	Average pain sensitive
AUDIT	Alcohol Use Disorders Identification Test
CFR	Code of Federal Regulations
CMP	Clinical Monitoring Plan
COMT	Catechol-O-methyltransferase
CROMS	Clinical Research Operations and Management Support
CSQ-R	Coping Strategies Questionnaire Revised
DCC	Data coordinating center
DC/TMD	Diagnostic Criteria for Temporomandibular Disorder
DNA	Deoxyribonucleic acid
DSMB	Data and Safety Monitoring Board
ECG	Electrocardiogram
EDC	Electronic data capture
FDA	Food and Drug Administration
FDAAA	Food and Drug Administration Amendments Act of 2007
GCP	Good Clinical Practice
GCPS	Graded Chronic Pain Scale
GWAS	Genome-Wide Association Studies
HIT-6	Headache Impact Test
HPS	High pain sensitive
ICH	International Conference on Harmonisation
ICMJE	International Committee of Medical Journal Editors
IND	Investigational New Drug (Application)
IRB	Institutional Review Board
LPS	Low pain sensitive
MOP	Manual of Procedures
NIDCR	National Institute of Dental and Craniofacial Research
NIH	National Institutes of Health
NRS	Numeric rating scale
NSAID	Nonsteroidal anti-inflammatory drug
OCTOM	Office of Clinical Trials and Operations Management
OHRP	Office of Human Research Protection
PGIC	Patient Global Impression of Change
PI	Principal Investigator
PPT	Pressure pain threshold
SAE	Serious adverse event
SCL-90R	Symptom Checklist 90-Revised

SF-12 v2	Short Form 12 Health Survey Version 2
TENS	Transcutaneous electrical nerve stimulation
TMD	Temporomandibular disorder
TMJ	Temporomandibular joint
UB	University at Buffalo
UF	University of Florida
UNC	University of North Carolina
UP	Unanticipated problem
US	United States

PROTOCOL SUMMARY

Title: Effect of *COMT* Genetic Polymorphisms on Response to Propranolol Therapy in Temporomandibular Disorder

Short Title: Study of Orofacial Pain and PropRANOlol (SOPPRANO)

Précis: A total of 200 participants of either sex and any race or ethnicity, recruited at 3 clinical sites, aged 18 to 65 years with chronic temporomandibular disorder (TMD), will be randomized in a 1:1 ratio to propranolol or placebo in a parallel-group, blinded clinical trial. Participants will attend 6 clinic visits over a period of 12 to 15 weeks.

Potential participants will be prescreened by telephone or at a clinic visit. During the Screening and Baseline Visit, participants will be consented, evaluated for eligibility, and assessed for baseline characteristics. Baseline procedures will include clinical tests and examinations and the administration of questionnaires. Additional questionnaires will be distributed to be completed and returned at the following visit.

At the Randomization and Titration Visit, participants will be randomized to propranolol or placebo and will begin a 10-week drug treatment phase that is divided into 1 week of drug titration, 8 weeks of drug maintenance, and 1 week of drug tapering. During treatment, many of the baseline assessments will be repeated. The final study visit will occur 1 week after drug tapering ends.

The primary endpoint will be a weekly pain index derived from a daily symptom diary. Secondary endpoints will be additional ratings of clinical pain; examiner assessments of experimental pain sensitivity; participant ratings of physical function, emotional function, and global improvement; occurrence of symptoms and adverse events (AEs); and use of rescue medications. DNA analyses will be conducted to identify polymorphisms in the gene that encodes catechol-O-methyltransferase (COMT), an enzyme that metabolizes catecholamines such as epinephrine, norepinephrine, and dopamine, and is associated with responses to pain.

Objectives: The primary objective is to investigate the efficacy of extended-release propranolol compared with placebo in the reduction of a pain index in participants with TMD at week 9 of treatment (at

	Visit 4).<br>The secondary objectives are as follows:<br>• To investigate gene-by-treatment group interaction to determine whether the efficacy of extended-release propranolol in the reduction of the pain index varies according to participants' polymorphisms in the coding region of the *COMT* gene.<br>• To investigate the efficacy of extended-release propranolol compared with placebo using secondary endpoints.<br>The exploratory objectives are as follows:<br>• To investigate whether the efficacy of extended-release propranolol in the reduction of the pain index varies according to participants' polymorphisms in 3 other genetic regions.<br>• To investigate whether the efficacy of extended-release propranolol in the reduction of the pain index varies according to various phenotypic characteristics.<br>• To collect and archive biospecimens for future biological and statistical analyses.
**Population:**	200 participants of either sex and any race or ethnicity between 18 and 65 years of age (inclusive) with chronic TMD will be randomized
**Phase:**	Phase II
**Number of Sites:**	Three clinical sites:<br>• University of North Carolina School of Dentistry at Chapel Hill, NC<br>• University of Florida College of Dentistry, Gainesville, FL<br>• University at Buffalo School of Dental Medicine, Buffalo, NY
**Study Duration:**	Approximately 3 years
**Participant Duration:**	12 to 15 weeks
**Description of Agent:**	Propranolol hydrochloride extended-release in 60 mg capsules taken orally twice a day over a 10-week treatment period
**Enrollment Duration:**	Approximately 2 years

Figure 1: Schematic of Study Design

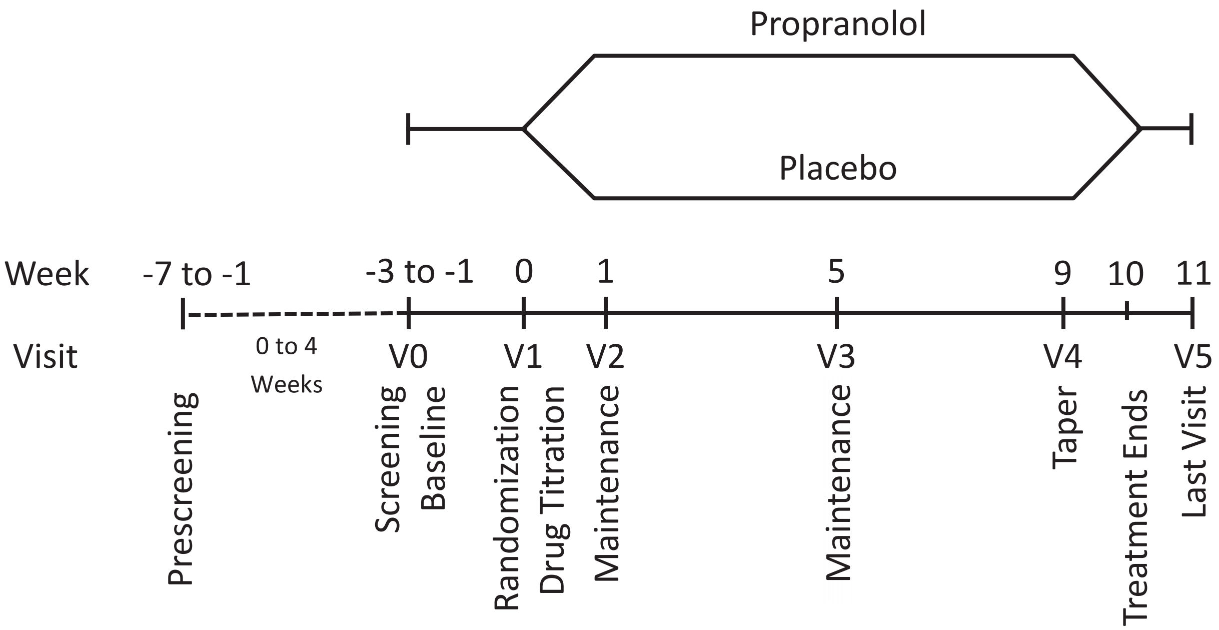

Prescreening
Day -49 to Day-7

Prescreen approximately 600 participants. Obtain verbal consent. Complete prescreening interview. Prescreen potential subjects by inclusion and exclusion criteria. Collect contact information.

Visit 0: Screening and Baseline
Day -21 to Day-7

Screen and baseline approximately 300 participants for study inclusion. Obtain informed consent. Screen potential subjects by inclusion and exclusion criteria; obtain history and Symptom Inventory, document. Perform baseline assessments (tender point and TMD exams, heat and pressure tests, 12-lead ECG) and urine pregnancy test (females). Dispense outcome measure questionnaires and daily symptom diaries.

Visit 1
Day 0

Obtain history and Symptom Inventory, document. Collect and assess daily symptom diaries. Assess AEs. Perform blood draw for DNA analyses, optional blood draw for future analyses, and urine pregnancy test (females). Review eligibility; collect outcome measure questionnaires.

Randomize and dispense study drug

Arm 1
100 subjects

Arm 2
100 subjects

Visit 2
Day 7 - 10

Obtain history and Symptom Inventory, document. Collect and assess daily symptom diaries and drug containers. Assess AEs. Perform urine pregnancy test (females). Administer Pain Questionnaire. Dispense study drug.

Visit 3
Day 35 ± 7

Obtain history and Symptom Inventory, document. Collect and assess daily symptom diaries and drug containers. Assess AEs. Perform urine pregnancy test (females), TMD exam, and heat and pressure tests. Administer outcome measure questionnaires. Dispense study drug.

Visit 4
Day 63 +6

Obtain history and Symptom Inventory, document. Collect and assess daily symptom diaries and drug containers. Assess AEs. Perform optional blood draw for future analyses, urine pregnancy test (females), TMD exam, and heat and pressure tests. Administer outcome measure questionnaires. Dispense study drug.

Visit 5
Day 77 + 7

Final Assessments
Obtain history and Symptom Inventory, document. Collect and assess daily symptom diaries and drug containers. Assess AEs. Perform urine pregnancy test (females). Administer Pain Questionnaire.

1 KEY ROLES

Co-Principal Investigators:

Inna Tchivileva, MD
Research Assistant Professor
5417J Koury Oral Health Sciences
School of Dentistry
UNC Chapel Hill
CB# 7455
Chapel Hill, NC 27599-7455
Phone: (919) 537-3291
Fax: (919) 966-5339
E-mail: chivilei@dentistry.unc.edu

Gary Slade, BDSc, Dip DPH, PhD
Distinguished Professor
5417B Koury Oral Health Sciences
School of Dentistry
UNC Chapel Hill
CB# 7455
Chapel Hill, NC 27599-7455
Phone: (919) 537-3273
Fax: (919) 966-5339
E-mail: gary_slade@dentistry.unc.edu

Roger Fillingim, PhD
Professor
College of Dentistry, University of Florida
1329 SW 16th Street
PO Box 103628
Gainesville, FL 32610-3628
Phone: (352) 273-5963
Fax: (352) 273-5985
E-mail: rfilling@ufl.edu

Richard Ohrbach, DDS, PhD
Associate Professor
University at Buffalo School of Dental Medicine
355 Squire Hall
Buffalo, NY 14214-1412
Phone: (716) 829-3590
Fax: (716) 829-3554
Email: ohrbach@buffalo.edu

NIDCR Medical Monitor:
Kevin McBryde, MD
National Institute of Dental and Craniofacial Research/NIH
6701 Democracy Blvd. Room 638 Bethesda, MD 20892
Phone: (301) 594-0170
Fax: (301) 480-8319
Email: mcbrydekd@nidcr.nih.gov

NIDCR Program Official:
Gallya Gannot, DMD, PhD
National Institute of Dental and Craniofacial Research/NIH
6701 Democracy Boulevard, Room 632
Bethesda, MD 20892-4878
Phone: (301) 451-5096
E-mail: gallya.gannot@nih.gov

Co-Investigators:
Alan Hinderliter, MD
Associate Professor
6th floor, Burnett-Womack Bldg., Division of Cardiology
Campus Box 7075
Chapel Hill, NC 27599-7075
Phone: (919) 843-5447
Fax: (919) 966-1743
E-mail: alan_hinderliter@med.unc.edu

Pei Feng Lim, BDS, MS
Clinical Associate Professor
5417G Koury Oral Health Sciences Building
School of Dentistry
UNC Chapel Hill
CB# 7455
Chapel Hill, NC 27599-7455
Phone: (919) 265-3023
Fax: (919) 966-2991
E-mail: peifeng_lim@dentistry.unc.edu

Margarete Ribeiro-Dasilva, DDS, PhD
1395 Center Drive, D9-22
Gainesville, FL 32610
Phone: (352) 273-7954
Fax: (352) 846-1643
E-mail: mdasilva@dental.ufl.edu

David Sheps, MD, MSPH
2004 Mowry Road, Room 4234
Gainesville, FL 32610-3010
Phone: (352) 294-5947
Fax: (352) 273-5365
E-mail: dssheps@phhp.ufl.edu

John Campbell, DDS
Associate Professor
University at Buffalo School of Dental Medicine
112 Squire Hall
Buffalo, New York 14214
Phone: (716) 829-2722
Fax: (716) 829-3019
E-mail: jc294@buffalo.edu

Thomas Cimato, MD, PhD
Associate Professor
875 Ellicott Street, Suite 7030
Division of Cardiovascular Medicine
Clinical and Translational Research Center
Buffalo, New York 14203
Phone: (716) 829-2663
Fax: (716) 854-1840
E-mail: tcimato@buffalo.edu

Institutions:

The University of North Carolina at Chapel Hill
School of Dentistry
Manning Drive & Columbia Street
Campus Box 7450
Chapel Hill, NC 27599-7450

The University of Florida
College of Dentistry
1600 SW Archer Road
Gainesville FL 32610-3003

The University at Buffalo
School of Dental Medicine
3435 Main St #32
Buffalo, NY 14214-1412

2 INTRODUCTION: BACKGROUND INFORMATION AND SCIENTIFIC RATIONALE

2.1 Background Information

2.1.1 Background on Temporomandibular Disorder

The term “temporomandibular disorder” (TMD) encompasses all musculoskeletal disorders of the masticatory system.[1] Common TMDs include myalgia, arthralgia, temporomandibular joint (TMJ) disc displacements with and without reduction, and TMJ degenerative joint diseases. A recent study reported that the most prevalent TMD diagnostic group is muscle dysfunction.[2] TMD is among the most common chronic musculoskeletal pain conditions and has significant societal costs.[3] The prevalence of TMD ranges from 6% to 12% in the general population.[4] Overall, TMD results in 17.8 million lost work days per year for every 100 million working adults in the US.[5] Chronic TMD is associated with substantial disability and suffering, and it negatively impacts the quality of life.[6] Jaw pain is the most common symptom that compels patients to seek treatment. One study reported an average TMD pain intensity rating of 4.3 on a 11-point scale, similar to the averages reported for chest pain and back pain.[7] In addition to facial pain, TMD patients frequently report various comorbid idiopathic pain conditions such as headaches, low back pain, and fibromyalgia.[8, 9] Novel approaches to TMD therapy are urgently needed to improve clinical outcomes and reduce the economic impact of this debilitating disorder.

2.1.2 Current Pharmacologic Management of TMD

Although there is currently no medicinal product labeled by the Food and Drug Administration (FDA) specifically for the management of TMD, several classes of drugs are used to attempt to relieve TMD-associated pain, such as nonsteroidal anti-inflammatory drugs (NSAIDs), corticosteroids, benzodiazepines, sedative hypnotics, muscle relaxants, opioids, antidepressants, and anticonvulsants. However, evidence clearly establishing efficacy and safety of these drugs in the management of TMD is scarce.[10, 11] The justification for their use is often based on uncontrolled clinical reports claiming efficacy, poorly controlled clinical trials, or clinical trials in other pain disorders (e.g., acute postsurgical dental pain, arthritic pain, chronic lower back pain, and neuropathic pain). There is a growing need for controlled clinical trials in the TMD population.

2.1.3 Adrenergic System in Pathophysiology of TMD

The physiological mechanisms responsible for TMD symptoms are poorly understood. A growing body of evidence suggests that enhanced β-adrenergic drive contributes to the pathogenesis of TMD and other complex persistent pain conditions. For example, individuals

with myofascial pain conditions have elevated catecholamine levels and augmented sympathetic responses to stressors.[12, 13] Additionally, fibromyalgia patients have higher basal norepinephrine levels and exaggerated norepinephrine responses to the proinflammatory cytokine, interleukin-6.[14] In animal models, epinephrine produces mechanical hyperalgesia and sensitized cultured dorsal root ganglion neurons via action at a β-adrenergic receptor.[15]

While increased β-adrenergic drive appears to heighten pain, β-adrenergic antagonists can reduce clinical pain and/or nociceptive sensitivity. A recent study of a single-dose infusion of propranolol in TMD and fibromyalgia patients revealed a short-term improvement in clinical pain ratings.[16] The mixed β-adrenergic receptor/5-hydroxytryptamine $(HT)_{1A}$ presynaptic receptor antagonist pindolol was similarly efficacious in alleviating the cardinal symptoms of fibromyalgia, namely pain, stiffness, and nonrestful sleep.[17] In a rodent model of TMD, both the nonselective β-adrenergic receptor antagonist propranolol and a selective β_2-blocker impaired pain behavior.[18] In addition, the intramuscular injection of low-dose propranolol in rats reduced inflammatory pain associated with carrageenan-induced inflammation of the gastrocnemius muscle.[16]

2.1.4 Background on Propranolol

The study hypothesis is that low-dose therapy with the nonselective β-adrenergic receptor antagonist propranolol will provide an efficacious and safe treatment for painful TMD. Extended-release propranolol is approved for the treatment of hypertension, angina pectoris due to coronary atherosclerosis, migraine, and hypertrophic subaortic stenosis.[19] It has well-studied pharmacodynamic and pharmacokinetic characteristics and side-effect profile. Peak blood level following dosing with an extended-release formulation of propranolol hydrochloride occurs at approximately 6 hours, and the apparent plasma half-life is approximately 10 hours. Data from our preliminary study suggest that low-dose short-acting propranolol produces minimal side effects (e.g., mild nausea, diarrhea, and fatigue occurred at similar rates during propranolol and placebo treatment).[20]

2.1.5 Background on Catechol-O-Methyltransferase as a Genetic Marker of Response to Propranolol

Abnormalities in catecholamine physiology are associated with diminished activity of catechol-O-methyltransferase (COMT), a ubiquitously expressed enzyme that metabolizes catecholamines such as epinephrine, norepinephrine, and dopamine.[21] TMD patients exhibited lower COMT activity relative to pain-free controls.[22] Our group and others have shown that various functional polymorphisms in the *COMT* gene that result in 3- to 15-fold reductions in COMT activity[23, 24] are associated with fibromyalgia,[25] migraine,[26] experimental pain sensitivity,[27, 28] and increased morphine efficacy in cancer pain treatment.[29]

Three major haplotypes of *COMT*, designated as low pain sensitive (LPS), average pain sensitive (APS), and high pain sensitive (HPS), accounted for 11% of variability in experimental

pain sensitivity in women.[27, 30] The LPS haplotype produces higher levels of COMT enzymatic activity than the APS or HPS haplotypes.[31] In our recent prospective cohort study, the presence of even a single LPS haplotype was found to diminish the risk of developing TMD as much as 2.3-fold.[27] To identify the receptor mechanism whereby elevated catecholamine levels resulting from reduced COMT activity modulate pain sensitivity, our group conducted a set of behavioral pharmacologic studies in rats receiving a COMT inhibitor. We found that COMT inhibition enhanced mechanical and thermal pain sensitivity comparable to that produced by carrageenan, a chemical well known for its ability to sensitize nociceptors and increase pain behavior. COMT-related mechanical and thermal hyperalgesia was blocked by propranolol or by combined administration of selective β_2- and β_3-adrenergic receptor antagonists but not by dopaminergic, α-, or β_1-adrenergic antagonists, suggesting that the hyperalgesia is selectively mediated by a $\beta_{2/3}$-adrenergic mechanism.[32]

We hypothesize that individuals with lower COMT activity, such as those not carrying the LPS haplotype, will experience relatively greater reduction in TMD pain from propranolol therapy than individuals with other *COMT* haplotypes that confer greater COMT activity. Genotyping of the *COMT* gene will allow us to evaluate this hypothesized gene-by-treatment group interaction, potentially identifying a subset of TMD patients most likely to benefit from propranolol treatment.

2.2 Rationale

A randomized, double–blind, placebo-controlled, crossover pilot study with a 1-week treatment with propranolol (20 mg twice a day) has been completed in patients with painful TMD.[20] In this study, the number of patients reporting a reduction in pain intensity rating was greater during propranolol treatment ($p = 0.014$) compared with placebo. Propranolol also significantly reduced the composite pain index ($p = 0.02$), but did not decrease other clinical and experimental pain ratings. When stratified by the *COMT* high-activity haplotype (LPS), a beneficial effect of propranolol on pain perception was noted in participants not carrying this haplotype, a diminished benefit was observed in the heterozygotes, and no benefit was noted in the LPS homozygotes; this effect was statistically significant in many pain measures. In the present trial, the duration of therapy for TMD with propranolol will be extended to 10 weeks, an extended-release instead of short-acting formula of the drug will be administered to enhance patient compliance over a longer treatment period, and the propranolol extended-release dose will be increased to 60 mg twice a day. Additionally, a parallel instead of crossover design will be utilized to reduce the drop-out rate and avert potential carryover effects.

Consensus recommendations for clinical trials of pain from the IMMPACT group[33] encourage investigators to evaluate five outcomes related to pain: (1) pain; (2) physical functioning; (3) emotional functioning; (4) participant global ratings of improvement; and (5) symptoms and adverse events (AEs). They also encourage investigators to collect data to permit reporting of study conduct consistent with the recommendations of the CONSORT group.[34] Accordingly, this protocol proposes endpoints and data recording that will address the requirements of both sets of recommendations.

2.3 Potential Risks and Benefits

2.3.1 Potential Risks

Investigational Drug. The investigational product is propranolol hydrochloride extended-release capsules. Propranolol has been available commercially for over 40 years and is routinely used to treat hypertension, migraine, and essential tremor at doses much greater than will be administered in this study. As propranolol is FDA approved for many disorders and has well-characterized pharmacological and side effects profiles, the risks for TMD patients using this drug at the proposed dose are low.

The package insert for propranolol hydrochloride extended-release capsules is shown in Appendix B. Propranolol is contraindicated in patients with cardiogenic shock, sinus bradycardia and greater than a first degree block, bronchial asthma, and known hypersensitivity to propranolol hydrochloride. Additionally, propranolol is not recommended for use in patients with cardiac failure, nonallergic bronchospasm, major surgery, diabetes, thyrotoxicosis, or Wolff-Parkinson-White syndrome. Patients with the above-listed conditions will not be included in the study. At all clinic visits after Prescreening, resting blood pressure and heart rate will be monitored. A 12-lead electrocardiogram (ECG) will be performed at the Screening and Baseline Visit. The ECG will be reviewed by a cardiologist who will determine the clinical significance of any ECG abnormalities. Participants will be asked to report any planned surgical procedures to the study principal investigators (PIs) since propranolol hydrochloride extended-release may increase the risks associated with general anesthesia and surgical procedures.

In a study in which male and female rats were exposed to propranolol hydrochloride in their diets, from 60 days prior to mating and throughout pregnancy and lactation for two generations, there were no effects on fertility. Contraception in males will not be required in this study. However, there are no adequate and well-controlled studies of propranolol in pregnant women.

Pregnancy is an exclusion criterion and a participant discontinuation criterion in this study. A pregnancy test will be conducted on all women of childbearing potential at all visits. In addition, propranolol is excreted in human milk; therefore, nursing is an exclusion criterion for this study.

Cardiovascular, central nervous system, gastrointestinal, allergic, respiratory, hematologic, autoimmune, skin and mucous membranes, and genitourinary AEs have been observed in patients using propranolol. All reportable AEs will be collected from the Screening and Baseline Visit through the last clinic visit.

Electrocardiogram. Electrode stickers placed on the participant’s skin may cause discomfort when removed. Trained personnel will conduct the procedure.

Questionnaires. A participant may experience discomfort associated with being asked personal questions about her or his health history, symptoms, or emotional feelings. Participants will be told that they may choose not to answer any questions that cause discomfort.

TMD Examination and Psychophysical Tests. Four assessments of this kind will be performed: the TMD examination (Section 7.1.6), tests for heat sensitivity (Section 7.1.7), tests for pressure pain threshold (PPT) (Section 7.1.8), and a manual tender point examination (Section 7.1.9). Assessments of muscle and TMJ sensitivity to digital palpation during the TMD examination, responses to masticatory muscle pressure threshold tests, and responses to painful heat stimuli are designed to evoke brief pain or discomfort during application of the stimulus; however, none of these assessments are expected to result in lasting discomfort or damage to affected tissues. Transient redness or tenderness of skin is possible after tests. All testing modalities will be conducted within a range of stimulus intensities that produce a level of pain or discomfort that is acceptable to the participant. No test will exceed this range, and all tests will end upon completion of the modality or upon the participant’s request, whichever comes first.

Blood Collection. There is a possibility of mild pain and bruising associated with a blood draw. Trained personnel will perform the blood collection using standard procedures.

Violation of Confidentiality. The risk of violation of confidentiality exists because human participants are providing personal information, including genetic data. Procedures to minimize risks to confidentiality are described in Section 14.4.

2.3.2 Known Potential Benefits

Participants may or may not benefit from receiving the investigational drug by experiencing decreased pain and improved functionality. In the absence of any approved TMD medication, this search for an efficacious, safe, and affordable treatment may benefit many TMD patients in the future. In addition, the proposed clinical trial will advance our knowledge of the role of the adrenergic system in the pathogenesis of TMD.

2.4 Study Objectives

2.4.1 Primary Objective

The primary objective is to investigate the efficacy of extended-release propranolol compared with placebo in the reduction of a pain index (defined in Section 2.5.1) in participants with TMD at week 9 of treatment (at Visit 4).

2.4.2 Secondary Objectives

The secondary objectives are as follows:

- To investigate gene-by-treatment group interaction to determine whether the efficacy of extended-release propranolol in the reduction of the pain index varies according to participants' polymorphisms in the coding region of the *COMT* gene.
- To investigate the efficacy of extended-release propranolol compared with placebo using secondary endpoints, namely: (1) additional pain ratings; (2) examiner assessments of experimental pain sensitivity; (3) participant ratings of physical function, emotional function, and global impression of change; (4) occurrence of symptoms and AEs; and (5) use of rescue medications.

2.4.3 Exploratory Objectives

The exploratory objectives are as follows:

- To investigate whether the efficacy of extended-release propranolol in the reduction of the pain index varies according to participants' polymorphisms including, but not limited to three other genetic regions: 1) the *COMT* promoter area; 2) adrenergic receptor β2 *(ADRB2)* gene; and 3) the adrenergic receptor β3 *(ADRB3)* gene.
- To investigate whether the efficacy of extended-release propranolol in the reduction of the pain index varies according to various phenotypic characteristics.
- To collect and archive biospecimens for future biological and statistical analyses.

2.5 Study Outcome Measures

2.5.1 Primary Outcome Measures

The primary endpoint will be a weekly mean pain index, representing the arithmetic mean of daily pain index values during the week prior to the study visit. The pain index will be computed during analysis as the product of the pain intensity score, reported on a 0-100 numeric rating scale (NRS), multiplied by the pain duration score, reported on a 0-100 percentage scale, each as reported in the Daily Symptom Diary. The weekly mean pain index will be computed for the week prior to randomization and for each week during the treatment phase. The weekly mean pain index will be calculated if a participant completes at least 4 of the 7 daily reports of pain intensity and duration during the 7-day period prior to the visit. When there are fewer than 4 daily reports, the weekly mean pain index will be considered missing.

2.5.2 Secondary Outcome Measures

DNA analyses of blood samples will be conducted to identify polymorphisms in the gene that encodes catechol-O-methyltransferase *(COMT)*. Assessments of pain intensity (NRS 0-100) and pain duration (0-100 percentage scale) from the Daily Symptom Diary will be used as secondary outcomes. Additional assessments of clinical pain, heat and pressure pain, jaw function, health status, emotional status, participant ratings of global improvement, sleep, somatic symptoms, AEs, and the use of rescue medication will be made by questionnaires and examinations, as described in Section 7.1.

3 STUDY DESIGN OVERVIEW

Two hundred participants with chronic TMD recruited at 3 clinical sites, will be randomized in a 1:1 ratio to propranolol or placebo in a parallel-group, blinded clinical trial. Participants will be limited to age 18 – 65 years because pharmacokinetic data are not available for patients over 65 years for extended-release propranolol hydrochloride; standard propranolol drug clearance is decreased and half-life increased in older populations. Additionally, with aging, arthralgia, osteoarthritis, and osteoarthrosis become more prevalent TMD diagnoses than myalgia; propranolol will not be effective for these types of TMD.[35]

Participants will attend 6 clinic visits over a period of 12 to 15 weeks (Figure 1). The study should be completed in 3 years, with participant recruitment occurring during years 1 and 2.

Prescreening will be performed either in the clinic or over the telephone, and potentially eligible participants will be scheduled for a Screening and Baseline Visit (Visit 0). During Visit 0, participants will be consented and evaluated for eligibility, and information including demographics, medical history, and concomitant medications will be collected. Additionally, participants will be assessed for pain, health status, jaw function, emotional functioning, sleep, and somatic symptoms, among other measures. Procedures will include clinical examinations and tests and the dispensation of various questionnaires, all of which are listed in the Schedule of Events (Appendix A).

At Visit 1, participants who show compliance with the Daily Symptom Diary (at least 4 daily entries within 7 days) and meet the pain score and all other eligibility requirements will be randomized to propranolol or placebo and will begin a 10-week drug treatment phase that is divided into 1 week of drug titration, 8 weeks of drug maintenance, and 1 week of drug tapering. During that period, Visits 2-4 will take place, and many of the baseline assessments will be repeated. The final study visit, Visit 5, will occur 1 week after drug tapering ends and 2 weeks after Visit 4.

The primary endpoint will be the weekly pain index derived from the Daily Symptom Diary. Efficacy will be evaluated as the difference between the propranolol and placebo arms in the mean change in the pain index during treatment. In addition, variation in efficacy will be compared between groups of participants classified according to polymorphisms in the coding region of the *COMT* gene.

Enrollment will not be stratified according to *COMT* genotype because in the pilot study sample, the haplotype variants were sufficiently distributed for statistical evaluation of a treatment-haplotype interaction without stratified randomization. In the pilot study, 30% of participants had no copies of the LPS haplotype, 50% had one copy, and 20% had two copies. Stratification by *COMT* genotype would significantly increase the complexity of enrollment and could delay the start of treatment for participants while their DNA test results were pending. The DNA analyses will be conducted after all participants have been enrolled.

4 STUDY ENROLLMENT AND WITHDRAWAL

4.1 Participant Inclusion Criteria

A participant must meet all of the following inclusion criteria to be eligible for initial enrollment and randomization:

1) Provides a signed and dated informed consent form
2) Is between 18 and 65 years of age (inclusive; male or female and any race or ethnicity)
3) Meets diagnostic criteria for TMD, Group II: Masticatory Muscle Disorders, 1A: Myalgia[36]
4) Has experienced facial pain for at least 3 months
5) Has experienced facial pain for at least 10 days of the last 30 days
6) At Screening and Baseline Visit (Visit 0), reports an average pain intensity rating over the past week of ≥ 30 on a numerical rating scale (0-100)
7) At randomization (Visit 1), has satisfactorily completed at least 4 entries per week in the Daily Symptom Diary within the preceding week and reported an average pain intensity score ≥ 30 on a numerical rating scale (0-100) or reports a rating of average daily pain intensity of ≥30 on the same scale on at least 3 days over the past week
8) If taking a prescription medication (with the exception of prescription formulations of NSAIDs, acetaminophen, and aspirin) episodically (or as needed) for the management of pain, agrees to discontinue its use prior to or at the Screening and Baseline Visit
9) If taking a prescription medication daily for the management of pain, agrees to continue the daily use of the medication throughout the study
10) If taking an over-the-counter pain medication daily, agrees to continue the daily use of the medication throughout the study
11) Agrees to not commence any new prescription medication for the management of pain throughout the study
12) Agrees to not commence any injection therapy for pain (e.g., tender or trigger point injections, steroid injections) during the course of the study
13) Agrees to not use acupuncture, biofeedback, or transcutaneous electrical nerve stimulation (TENS) for the management of pain during the course of the study
14) Agrees to not commence occlusal splint therapy during the course of the study
15) Agrees to continue use of splint therapy if commenced more than 30 days previously

16) Females agree to limit consumption of alcohol to no more than 7 drinks* and males agree to limit consumption of alcohol to no more than 14 drinks* a week for the duration of the study

17) Females of childbearing potential, agrees to use one of the following methods of contraception throughout the study: licensed hormonal method, intrauterine device, condoms with contraceptive foam, abstinence, or vasectomy in partner (if post-menopausal, must not have menstruated for at least 12 consecutive months)

18) Willing and able to understand and comply with all study procedures and be available for the duration of the study

4.2 Participant Exclusion Criteria

A potential participant who meets any of the following criteria will be excluded from enrollment and randomization:

1) Has a history of congestive heart failure or the presence of any of the following cardiovascular conditions: clinically significant abnormal 12-lead ECG, sinus bradycardia (resting heart rate below 55 beats per minute although lower heart rates will be acceptable upon the review and approval by the site cardiologist), greater than first degree heart block, coronary artery disease, uncontrolled hypertension, or hypotension (systolic blood pressure below 90 mm Hg). In individuals with a heart rate below 55 beats per minute, the site cardiologist will review the participant's medical history, including any concomitant medications, to ensure that they are in good general health and have no medical contraindications to taking propranolol.

2) Has any of the following medical conditions: bronchial asthma, nonallergic bronchospasm (chronic obstructive pulmonary disease and emphysema), renal failure or dialysis, diabetes mellitus, hyperthyroidism, fibromyalgia, or uncontrolled seizures

3) Has known hypersensitivity to propranolol or components of the placebo capsules

4) Currently taking a β-blocker or medication that may interact with propranolol, including haloperidol, intravenous verapamil, and catecholamine-depleting drugs, such as reserpine, within 30 days prior to the Screening and Baseline Visit

5) Currently taking an opioid medication, whether episodically or daily, within 30 days prior to the Screening and Baseline Visit

6) Has commenced a new daily prescription medication for the management of pain within 30 days prior to the Screening and Baseline Visit

* one standard drink is defined as 12 ounces of regular beer, 5 ounces of wine, or 1.5 ounces of 80 proof distilled spirits.

7) Used any injection therapy (e.g., tender or trigger point injections, steroid injections) for the management of pain within 2 weeks prior to the Screening and Baseline Visit

8) Used acupuncture, biofeedback, or TENS for the management of pain within 2 weeks prior to the Screening and Baseline Visit

9) Has commenced occlusal splint therapy for the management of facial pain within 30 days prior to the Screening and Baseline Visit

10) Has experienced facial trauma or orofacial surgery within 6 weeks prior to the Screening and Baseline Visit

11) Is undergoing current active orthodontic treatment (passive retainers are permitted)

12) Has a history of major depression or other major psychiatric disorder requiring hospitalization within the last 6 months prior to the Screening and Baseline Visit

13) Has a history of treatment for drug or alcohol abuse within the last year

14) Has scored 8 or more on the Alcohol Use Disorders Identification Test (AUDIT) at the Screening and Baseline Visit

15) Currently smokes at least 25 cigarettes per day

16) Currently being treated with chemotherapy or radiation therapy

17) Has been treated with another investigational drug or treatment within 30 days prior to the Screening and Baseline Visit

18) Is pregnant or nursing

19) Anything that, in the opinion of the investigator, would place the participant at increased risk or preclude the participant’s full compliance with or completion of the study

4.3 Strategies for Recruitment and Retention

Participants will be recruited from existing TMD patient registries and from various clinics at the three universities. Clinicians at these sites will be made aware of the study and asked to inform their patients of the study and to provide their patients with study contact information. In addition, participants may be recruited through print and electronic media advertisements. All recruitment materials will be submitted to the Institutional Review Boards (IRBs) for review and approval prior to their use.

Participants will be reminded of each upcoming visit by telephone, text message, email, or letter. Participants will be told about the importance of keeping each scheduled appointment, and they will be instructed to contact the study staff as soon as possible if they are unable to attend a scheduled appointment. Participants who miss a scheduled visit without notifying the study staff will be contacted by one of the previously mentioned methods to encourage their

continued participation in the study and to ask them to contact the study team concerning their next appointment. Unscheduled visits may occur at the investigator's discretion (Section 6.6).

Participants will be compensated for their participation in the study in an amount stated in the informed consent form, and in some cases, the participant may be eligible for reimbursement of some travel expenses.

4.4 Treatment Assignment Procedures

4.4.1 Randomization Procedures

Prior to randomization, the inclusion and exclusion criteria will be reviewed, and any participant who does not meet the criteria with be discontinued from the trial. Randomization will be stratified across 3 clinical sites for a total of 200 randomized participants. Within each site, participants will be randomized in a 1:1 ratio of active drug to placebo within prespecified blocks. Randomization will be accomplished by a web-based randomization system developed and maintained by the data coordinating center. At the time of randomization, study staff, who will remain blinded to treatment assignment, will access the system, enter the participant's study identification number, and verify that the participant is eligible for randomization. The system will assign a randomization code that will indicate to the pharmacy the participant's treatment assignment.

4.4.2 Blinding Procedures

All study staff, including the study clinicians and investigators, will be blinded to the participants' treatment assignments throughout the data collection period. Propranolol hydrochloride extended-release capsules will be over-encapsulated and cannot be differentiated from the placebo capsules by appearance alone. The research pharmacies will be unblinded. The data coordinating center (DCC) project staff will remain blinded through the analysis for the primary objective. If oversight boards, such as the IRBs or Data and Safety Monitoring Board (DSMB), request an unblinded data report during the data collection period, an unblinded biostatistician at the DCC will generate the report.

4.4.3 Unblinding Procedures

Unblinding before the study is completed will occur only if a participant's well-being is threatened and is necessary to protect the participant. Except in extreme medical emergencies, before the treatment assignment for a participant is unblinded, the investigator must confer with the National Institute of Dental and Craniofacial Research (NIDCR) medical monitor and the site cardiologist as necessary. For extreme medical emergencies that occur during business hours, the clinical site investigator will contact the DCC to determine the participant's treatment assignment. For extreme medical emergencies that occur outside of business hours, medical personnel will contact the site pharmacy to determine the participant's treatment assignment. An event is considered an extreme medical emergency when appropriate medical management of

the participant necessitates knowledge of treatment assignment. Study participants will be provided with instructions and contact information for emergency situations. The emergency unblinding will be recorded and reported to the PI and to the NIDCR medical monitor as soon as possible. A full account of the event will be recorded, including the date and time of the emergency, the reason for unblinding, and the names of the medical monitor and others who were notified of the emergency. The unblinding will be reported to the DSMB and the IRBs according to reporting guidelines for those boards.

4.4.4 Reasons for Participant Withdrawal

Participants are free to withdraw from participation in the study at any time, and the investigator may withdraw a participant for any of the following reasons:

- Withdrawal of consent.
- Does not meet criteria for randomization.
- Pregnancy.
- Continued participation deemed to be not in the participant's best interest.
- Investigator discretion.

Participants requiring significant surgery will be withdrawn from the protocol. Investigators will use discretion in making this determination. Participants withdrawn after randomization will not be replaced. If a participant does not return for a scheduled visit, every effort should be made to contact the participant and document the outcome. If the participant withdraws consent, no further evaluations will be performed, and no attempts will be made to collect additional data.

4.4.5 Handling of Withdrawals and Discontinuation

Participants who withdraw from the study voluntarily or are withdrawn by an investigator will be discontinued from the study. Study staff will complete a study disposition form in the electronic data capture (EDC) system, indicating the reason for discontinuation. If the participant is on study drug at discontinuation, the investigators, in consultation with the site cardiologist, will decide the following: (1) the need for tapering of the investigational drug, (2) whether tapering will be conducted by the investigator prior to discontinuation or by a referring practitioner after discontinuation, and (3) whether unblinding of the treatment assignment will be required. If unblinding is required, the investigator will follow the unblinding procedures described in Section 4.4.3.

Any participant with an AE that is ongoing at the time of discontinuation will be followed until the event returns to baseline, resolves, or stabilizes. If the AE does not meet these outcomes within 30 days after discontinuation or the end of the study, the participant will be referred to an appropriate practitioner for continued care.

4.4.6 Suspension or Termination of the Study

The study may be suspended or prematurely terminated by the NIDCR if reasonable cause exists, as determined by the PI, the NIDCR program official or medical monitor, the IRB, or the DSMB. Circumstances that may warrant suspension or termination include, but are not limited to, the following:

- Determination of unexpected, significant, or unacceptable risk to participants.
- Insufficient adherence to protocol requirements.
- Data that are insufficient and/or not evaluable.

If the study is prematurely suspended or terminated, the PI will provide written notification to the investigators, the NIDCR program official, the IRB, and the DSMB.

5 STUDY INVESTIGATIONAL PRODUCT AND PLACEBO

The Investigators consulted with the FDA to determine whether this protocol should be conducted under an IND. The FDA determined that this clinical trial meets the exemption requirements for an IND. An overview of the investigational product and placebo are provided below; further details are available in the Manual of Procedures (MOP).

5.1 Investigational Product

The investigational product is propranolol hydrochloride extended-release capsules, an approved drug for the treatment of hypertension, angina pectoris due to coronary atherosclerosis, migraine, and hypertrophic subaortic stenosis. The package insert for propranolol hydrochloride extended-release is provided in Appendix B. Propranolol hydrochloride extended-release is a nonselective β-adrenergic receptor-blocking agent. The extended-release formula provides a sustained release of propranolol hydrochloride.

5.1.1 Acquisition

Propranolol hydrochloride extended-release 60 mg capsules will be acquired from a compounding pharmacy and shipped to the research pharmacy at each clinical site. The research pharmacies will dispense the drug.

5.1.2 Formulation, Packaging, and Labeling

The propranolol hydrochloride extended-release capsules will be over-encapsulated by the compounding pharmacy to have the same appearance as the placebo. Neither the study personnel nor the study participants will be able to differentiate the study drug from the placebo by appearance alone.

The research pharmacy will label and dispense the 60 mg capsules in tight, light-resistant containers. The containers will be labeled with the following information:

- Name and address of the dispensing pharmacy
- Protocol number
- Name of the drug labeled as “propranolol hydrochloride extended-release or placebo” to protect the blinding
- Strength of the drug (60 mg propranolol hydrochloride extended-release or placebo) and number of capsules dispensed
- Name of the prescribing clinician
- Instructions for use
- Participant name and medical record number
- Date dispensed
- The following statement: “Limited by Federal Law to Investigational Use Only”

5.1.3 Product Storage and Stability

Propranolol hydrochloride extended-release capsules will be shipped and stored according to United States Pharmacopeial Convention (USP) Controlled Room Temperature guidelines and protected from light, moisture, freezing, and excessive heat.

5.2 Dosage and Administration of Investigational Product

Propranolol hydrochloride extended-release 60 mg capsules will be dispensed at Visits 1, 2, 3, and 4 as described below:

- Visit 1 (beginning of the 1-week titration): 1 capsule per day in the evening
- Visit 2 (beginning of the first 4 weeks of maintenance): 2 capsules per day, one in the morning and one in the evening
- Visit 3 (beginning of the last 4 weeks of maintenance): 2 capsules per day, one in the morning and one in the evening
- Visit 4 (beginning of the 1-week tapering): 1 capsule per day in the evening

5.3 Modification of Dosage of Investigational Product

If a participant cannot tolerate the scheduled titration or maintenance dosage, they will remain in the study at either a lower, tolerated dosage or without taking study medication. These events will be recorded as protocol deviations.

5.4 Placebo

The placebo, which will be manufactured for this study, will be acquired, packaged, labeled, dispensed, and administered in the same manner as described above for the active drug.

Ingredients will be a microcrystalline cellulose filler and a gelatin capsule. Storage requirements for the placebo are the same as for the active drug.

5.5 Accountability Procedures

The research pharmacies will be responsible for maintaining either paper or electronic logs for the acquisition and dispensing of the active drug and placebo. The pharmacies will also be responsible for maintaining and monitoring storage conditions for the active drug and placebo. The logs and records of storage conditions will be reviewed by the clinical monitor during periodic monitoring visits.

5.6 Assessment of Participant Compliance

Participants will be asked to bring their medication containers to each visit during the treatment period. Study staff will count the number of capsules returned and will enter the number on a case report form in the EDC system. Participants will record the study medication use in the Daily Symptom Diary that will be reviewed by the study staff at Visits 2, 3, 4, and 5. Participant compliance with study medication will be defined as taking 60% or more of the study medication for the duration of the study.

5.7 Concomitant Medications and Therapies

Prior and concomitant medication information will be collected for all participants for the 30 days prior to the Screening and Baseline Visit and during the study. Information collected will include medication, total daily dose, start date, stop date (if applicable), and primary reason for use. Information about treatment with injection therapy, acupuncture, biofeedback, TENS, and occlusal splint therapy will also be collected for all participants for the 30 days prior to the Screening and Baseline Visit and during the study.

5.7.1 Rescue Medication

Rescue medications described below may be used to alleviate pain. Episodic use of a rescue medication will be defined as use for no more than 3 consecutive days and for no more than 18 total days from Visits 1 to 4.

5.7.1.1 Over-the-counter medications

NSAIDs are most often used to manage pain in TMD patients. If a participant is taking an over-the-counter medication daily for pain management, the participant will be encouraged to continue the same usage of that medication throughout the study. The usage of short-acting, non-prescription analgesics (e.g., NSAIDs, aspirin, and/or acetaminophen) by participants during the study will be recorded and quantified at each visit, and the usage will be classified as either daily or episodic.

5.7.1.2 Prescription medications

Participants who enter the study on a daily regimen of a prescription medication for pain will be encouraged to continue that regimen throughout the study. The usage of all prescription medications for pain during the study will be recorded and quantified at each visit.

5.7.2 Nonpharmacological Therapy

During the course of the trial, participants will not be restricted in the use of the nonpharmacological therapies, with the exception of acupuncture, biofeedback, and/or TENS. If, contrary to the study requirements, a participant starts acupuncture, biofeedback, and/or TENS treatment for the management of pain during the study, the use of these therapies will be recorded.

5.8 Protocol Deviations Related to the Use of Investigational Product and Concomitant Therapies

The following protocol deviations related to the use of investigational product and concomitant therapies will be considered major protocol deviations and reported on a case report form in the EDC system.

- Participant is unable to reach the maintenance dosage of the study drug or placebo or is unable to remain on the maintenance dosage.
- Initiation of opioid medications.
- Initiation of TMJ surgical treatment (including TMJ arthrocentesis).
- Initiation of therapeutic injections for the management of pain.
- Initiation of occlusal splint therapy.
- Initiation of any of the following nonpharmacological therapies: acupuncture, biofeedback, and/or TENS.
- Initiation after Visit 1 and usage of short-acting, non-prescription analgesics (e.g., NSAIDs, aspirin, and/or acetaminophen) for pain that exceeds the definition of episodic use as described in Section 5.7.1.

6 STUDY SCHEDULE

The Schedule of Events table, which provides an overview of the study phases, timeline, and events by visit, is provided in Appendix A. In the following section, events are listed by study phase and visit, though the order of events listed within a visit does not imply the order in which the events will occur. A description of the procedures is provided in Section 7.

6.1 Prescreening (Day -49 to Day-7)

Prescreening may occur by telephone or at a clinic visit, and it may be combined with the Screening and Baseline Visit (Visit 0). After obtaining verbal consent, a brief prescreening interview script will be administered and basic eligibility information, specific points of medical history, and contact information will be recorded. If a participant expresses interest in the study and if the participant is eligible, the participant will be scheduled for Visit 0 within 4 weeks.

6.2 Screening and Baseline Visit 0 (Day -21 to Day-7)

The following procedures and assessments will be conducted up until the time of screen failure:

1) Obtain consent for study participation
2) Obtain consent to store biological specimens for future studies
3) Obtain signed Health Insurance Portability and Accountability statement (as applicable)
4) Collect demographic information
5) Obtain medical history
6) Administer the AUDIT Questionnaire
7) Administer the Smoking Questionnaire
8) Administer the Symptom Inventory
9) Record concomitant medications
10) Record specified concomitant therapies
11) Collect physical measurements
12) Collect vital signs
13) Perform a urine pregnancy test in female participants of childbearing potential
14) Administer the Fibromyalgia Questionnaire
15) Perform the manual tender point examination
16) Administer the SF-McGill Pain Questionnaire
17) Perform TMD examination
18) Review eligibility criteria
19) Perform pressure and heat pain tests (baseline data)
20) Dispense outcome measure questionnaires as shown in Schedule of Events
21) Dispense new Daily Symptom Diaries
22) Schedule next visit

23) Perform a screening ECG

6.3 Treatment (Visits 1-4)

6.3.1 Randomization and Titration Visit 1 (Day 0)

The following procedures and assessments will be conducted at Visit 1:

1) Review medical history
2) Record the average number of alcoholic drinks consumed per week since the previous visit
3) Administer the Symptom Inventory
4) Record concomitant medications
5) Record specified concomitant therapies
6) Assess and record AEs
7) Collect vital signs
8) Collect Daily Symptom Diaries
9) Assess compliance with Daily Symptom Diaries
10) Collect outcome measure questionnaires as shown in Schedule of Events
11) Perform a urine pregnancy test in female participants of childbearing potential
12) Review eligibility criteria
13) Collect confidence in treatment outcome data
14) Perform randomization to study drug, if participant is eligible
15) Draw blood
16) Dispense new Daily Symptom Diaries
17) Schedule next visit
18) Dispense study drug

At least 4 entries in the Daily Symptom Diary must be completed satisfactorily within the week preceding this visit for the participant to meet the eligibility criterion for randomization. The participant must also have an average pain intensity score of ≥ 30 or reports a rating of average daily pain intensity of ≥ 30 on ate least 3 days in the preceding week, as calculated from the Daily Symptom Diary entries, to meet the eligibility criteria. If a participant meets these 2 eligibility criteria during this visit, the participant will be randomized and continue in the study.

6.3.2 Maintenance Visit 2 (Day 7 [+ 3 days])

The following procedures and assessments will be conducted at Visit 2:

1) Collect study drug containers issued at previous visit
2) Collect Daily Symptom Diaries
3) Assess participant compliance with Daily Symptom Diaries
4) Review medical history
5) Record the average number of alcoholic drinks consumed per week since the previous visit
6) Administer the Symptom Inventory
7) Record concomitant medications
8) Record specified concomitant therapies
9) Assess and record AEs
10) Collect vital signs
11) Perform a urine pregnancy test in female participants of childbearing potential
12) Administer the SF-McGill Pain Questionnaire
13) Dispense new Daily Symptom Diaries
14) Dispense study drug
15) Schedule next visit
16) When participant has left, assess participant compliance with study drug usage

6.3.3 Maintenance Visit 3 (Day 35 [± 7 days])

The following procedures and assessments will be conducted at Visit 3:

1) Collect study drug containers issued at previous visit
2) Collect all Daily Symptom Diaries (each diary covering one week)
3) Assess participant compliance with Daily Symptom Diaries
4) Review medical history
5) Record the average number of alcoholic drinks consumed per week since the previous visit
6) Administer the Symptom Inventory
7) Record concomitant medications
8) Record specified concomitant therapies

9) Assess and record AEs
10) Collect vital signs
11) Perform a urine pregnancy test in female participants of childbearing potential
12) Perform TMD examination
13) Perform heat and pressure pain tests
14) Administer outcome measure questionnaires as shown in Schedule of Events
15) Dispense new Daily Symptom Diaries
16) Dispense study drug
17) Schedule next visit
18) When participant has left, assess participant compliance with study drug usage

6.3.4 Tapering Visit 4 (Day 63 [+6 days])

The following procedures and assessments will be conducted at Visit 4:

1) Collect study drug containers issued at previous visit
2) Collect all Daily Symptom Diaries (each diary covering one week)
3) Assess participant compliance with Daily Symptom Diaries
4) Review medical history
5) Record the average number of alcoholic drinks consumed per week since the previous visit
6) Administer the Symptom Inventory
7) Record concomitant medications
8) Record specified concomitant therapies
9) Assess and record AEs
10) Collect vital signs
11) Perform a urine pregnancy test in female participants of childbearing potential
12) Perform TMD examination
13) Perform heat and pressure pain tests
14) Administer outcome measure questionnaires as shown in Schedule of Events
15) Dispense new Daily Symptom Diaries
16) Dispense study drug
17) Draw blood

18) Schedule next visit
19) When participant has left, assess participant compliance with study drug usage

6.4 Follow-up Visit 5 (Day 77 [+ 7 days])

The following procedures and assessments will be conducted at Visit 5:

1) Collect study drug containers issued at previous visit
2) Collect Daily Symptom Diaries (each diary covering one week)
3) Assess participant compliance with Daily Symptom Diaries
4) Review medical history
5) Administer Smoking Questionnaire
6) Record the average number of alcoholic drinks consumed per week since the previous visit
7) Administer the Symptom Inventory
8) Record concomitant medications
9) Record specified concomitant therapies
10) Assess and record AEs
11) Collect vital signs
12) Perform a urine pregnancy test in female participants of childbearing potential
13) Administer the SF-McGill Pain Questionnaire
14) When participant has left, assess participant compliance with study drug usage

6.5 Early Termination Visit

If a participant is discontinued before study completion, prior to the withdrawal of consent and with the participant's permission, an Early Termination Visit may be conducted, either during a scheduled or unscheduled visit. Each of the procedures and assessments described for Visit 4 (Section 6.3.4) and the administration of the Smoking Questionnaire will be conducted at the Early Termination Visit.

6.6 Unscheduled Visit

An unscheduled visit may occur at the discretion of the investigator. Reasons for an unscheduled visit would include, but are not limited to, an AE, the inability of the participant to attend a visit within the visit window, or the need for an Early Termination Visit.

7 STUDY PROCEDURES

Standard operating procedures for all study procedures will be detailed in the MOP, which will be provided to study staff prior to study start-up. Overviews of the procedures are provided below. Data collected during these procedures will be entered into case report forms within the EDC system or via Teleform.

7.1 Clinical Evaluations

7.1.1 Medical History

The medical history for each participant will be obtained by interview and entered into the case report form. Medical history will be updated at each visit.

7.1.2 Vital Signs and Physical Measurements

After the participant has rested in a chair for 10 minutes, trained personnel will take 3 readings of the participant's systolic blood pressure, diastolic blood pressure, and heart rate at 2-minute intervals. A physical examination will be performed at the Screening and Baseline Visit (Visit 0) and will include height and weight measurements.

7.1.3 Pregnancy Test

Because propranolol hydrochloride extended-release is not recommended for use during pregnancy (Appendix B), female participants of childbearing potential will have a urine pregnancy test (instant type) performed at all visits. The results will be read by a member of the study staff. The urine will be discarded after the test.

7.1.4 Blood Draw

Staff trained in phlebotomy will obtain up to 20 milliliters of blood by venipuncture. Two whole blood tubes for DNA analyses will be placed in short-term storage at each site at 4ºC, and 2 additional whole blood tubes will be processed to obtain plasma and red blood cells. Plasma and red blood cells for future cytokine and COMT activity analyses will be divided into aliquots and placed in storage along with the tubes for DNA analyses at -80ºC at each site until shipping to the facilities that will perform DNA extraction (Section 7.2.3).

7.1.5 Electrocardiogram

Trained personnel will administer a 12-lead ECG. The procedure involves having the participant lie prone on an examination table and placing electrode stickers on the participant's skin. The original ECG will be reviewed by a cardiologist. At the University of North Carolina (UNC), the ECG will be performed at the Clinical and Translational Research Center, and at the University of Florida (UF), the ECG will be performed at the Clinical Research Center.

7.1.6 Temporomandibular Disorder Examination

Trained and calibrated study staff will perform a TMD examination based on the Diagnostic Criteria for Temporomandibular Disorder (DC/TMD).[36] The examination assesses pain in masticatory muscles and the TMJ in response to palpation and maneuvering of the jaw. The examiner will bilaterally palpate the extraoral masticatory muscles (temporalis and masseter) and TMJ. For eligibility, the participant must meet 2 criteria relating to TMD: 1) reported pain or ache in the jaw, temple, face, preauricular area, or inside the ear that is changed by jaw function; and 2) finding(s) of TMD myalgia classified according to the DC/TMD criteria during the examination.

7.1.7 Tests for Heat Sensitivity

Trained study staff will perform the heat sensitivity tests. Contact heat stimuli will be delivered using a computer-controlled thermal sensory analyzer as described in the MOP. Thermal threshold and tolerance will be measured on the ventral forearm by an ascending method of limits. The thermal pain threshold will be defined as the temperature at which the participant first perceives heat pain, whereas thermal pain tolerance will be defined as the temperature at which the participant can no longer tolerate the pain. The temperature will increase from a baseline of 32°C with a 0.5°C/s rate of rise until the participant responds by pressing the button. The cutoff temperature for both measurements will be 50°C. Average thermal threshold and tolerance will be calculated from 4 assessments conducted with a 5-second interstimulus interval at different sites of the ventral forearm. For all heat tests, participants will be first given practice runs on a site distant from subsequent testing to verify the participant's understanding of the protocol.

7.1.8 Pressure Pain Threshold Measurements

Trained study staff will perform the PPT measurements. The PPT will be assessed bilaterally over the temporalis, masseter, and trapezius muscles, the TMJs, and the lateral epicondyles with a pressure algometer as described in the MOP. The PPT will be defined as the amount of pressure at which the participant first perceives the stimulus to be painful. One pre-trial assessment will be performed at each site followed by additional assessments until 2 measures differing by less than 0.2 kg are obtained, or 5 assessments are administered. In either case, the mean of the 2 closest values will be recorded as the threshold estimate. Pressure stimuli will be delivered at an approximate rate of 1 kg/s. The cutoff pressure for all sites will be 5 kg. The values from the right and left sides will be averaged to obtain a single PPT value per anatomical site.

7.1.9 Manual Tender Point Examination

The manual tender point examination will be performed by trained study staff, as a screening procedure. This assessment verifies a report of pain at a minimum of 11 of 18 specific locations (tender points), when palpated with approximately 4 kg of digital pressure. The 18 sites (9 pairs) examined are identical to those defined in the American College of Rheumatology (ACR) criteria

for the classification of fibromyalgia (1990),[37] which require a history of widespread pain present for at least 3 months with pain in 11 of 18 tender point sites on digital palpation. Pain is considered widespread when all of the following are present: pain in the left side of the body, pain in the right side of the body, pain above the waist, and pain below the waist. In addition, axial skeletal pain (cervical spine, anterior chest, thoracic spine, or low back) must be present. In this definition, shoulder and buttock pain is considered pain for each involved side. Low back pain is considered lower segment pain. Digital palpation should be performed with an approximate force of 4 kg. For a tender point to be considered positive, the participant must state that the palpation was “painful”; “tender” is not considered painful. For classification purposes, participants will be said to have fibromyalgia and be ineligible for study participation if both criteria are satisfied.

Pain, on digital palpation, must be present in at least 11 of the following 18 tender point sites:

1) Occiput (bilateral, at the suboccipital muscle insertions)
2) Low cervical (bilateral, at the anterior aspects of the intertransverse spaces at C5-C7)
3) Trapezius (bilateral, at the midpoint of the upper border)
4) Supraspinatus (bilateral, at origins, above the scapula spine near the medial border)
5) Second rib (bilateral, at the second costochondral junction, just lateral to the junctions on upper surfaces)
6) Lateral epicondyle (bilateral, 2 cm distal to the epicondyles)
7) Gluteal (bilateral, in upper outer quadrants of buttocks in anterior fold of muscle)
8) Greater trochanter (bilateral, posterior to the trochanteric prominence)
9) Knee (bilateral, at the medial fat pad proximal to the joint line)

7.1.10 Daily Symptom Diary

The participants will be asked to complete the Daily Symptom Diary at the end of each day starting at Visit 0 and ending at Visit 5. Participants who complete at least 4 out of the 7 entries per week at Visit 1 will be considered eligible for study participation. The diary requests information about the participant’s pain intensity (reported on a 0-100 numeric rating scale) and duration (reported on a 0-100 percentage scale), fatigue, sleep, and somatic symptoms. The participants will complete a Daily Symptom Diary on a new paper form each week of the study, as described in the MOP. The participants will return paper-based diaries either by mail or in person at study visits. Compliance with the Daily Symptom Diary will be defined as completion of at least 4 daily entries within 7 days. Any noncompliance found during Visits 2 through 5 will be reported as protocol deviations.

7.1.11 The Alcohol Use Disorders Identification Test

The Alcohol Use Disorders Identification Test is a method to screen for excessive drinking. [38] This questionnaire assesses recent alcohol use, alcohol dependence symptoms and alcohol-related problems. The AUDIT test will be performed at V0, as described in the MOP. Participants with a score of 8 or more will be ineligible for study participation.

7.1.12 Smoking Questionnaire

The Smoking Questionnaire is a method to screen for use of and exposure to tobacco smoke. This questionnaire assesses current and former use of combustible and smokeless tobacco, as well as current exposure to environmental tobacco smoke. The questionnaire will be administered at Visit 0 and Visit 5, as described in the MOP. Participants who self-report smoking at least 25 cigarettes per day at Visit 0 will be ineligible for study participation.

7.1.13 Symptom Inventory

The Symptom Inventory is a method to screen for AEs. Trained study staff will record participant responses to questions about the occurrence and severity of expected AEs known to be associated with propranolol hydrochloride extended-release. The Symptom Inventory will be administered at all visits, as described in the MOP. If a symptom has emerged or worsened, study personnel will evaluate the symptom for a possible AE.

7.1.14 Outcome Measure Questionnaires

The questionnaires that will be utilized are listed in the Schedule of Events (Appendix A) and are briefly described below. Copies of the questionnaires are reproduced in the attachments. Several questionnaires are self-administered, and others will be administered by trained study staff. For outcome measure questionnaires, responses will be recorded on paper Teleform case report forms, which will be scanned as described in the MOP.

Short-Form McGill Pain Questionnaire. This questionnaire consists of 15 descriptors (11 sensory, 4 affective) which are rated on an intensity scale as 0 (“none”), 1 (“mild”), 2 (“moderate”), or 3 (“severe”).[39]

Graded Chronic Pain Scale (GCPS). The GCPS comprises 7 items and assesses 2 dimensions of pain: pain intensity and pain-related disability.[7, 40]

Fibromyalgia Questionnaire. This questionnaire assesses the ACR criteria for fibromyalgia (1990)[37] and includes pain-related questions and manikin drawings of participant pain location.

Short Form 12 Health Survey Version 2 (SF-12 v2). The SF-12 v2 is a briefer measure of health-related quality of life derived by using 12 items from the original Short Form 36 instrument.[41] This questionnaire produces 2 scores: a mental health composite score and a physical health composite score.

Patient Global Impression of Change (PGIC). The PGIC is a participant-rated instrument that measures change in participant's overall status on a scale ranging from 1 ("very much improved") to 7 ("very much worse"). The PGIC is based on the Clinical Global Impression of Change, which is a validated scale.[42]

Jaw Functional Limitation Scale. This 20-item instrument measures limitations across 3 domains: mastication (6 items), vertical jaw mobility (4 items), and verbal and emotional expression (8 items).[43, 44] Two items are not scored as part of these 3 subscales. A degree of limitation is rated on a 0-10 scale from 0 ("no limitation") to 10 ("severe limitation").

Perceived Stress Scale. This questionnaire assesses 14 sources of stress and produces an overall perceived stress rating.[45]

Hospital Anxiety and Depression Scale. This 14-item instrument evaluates anxiety and depression.[46]

Symptom Checklist 90-Revised (SCL-90R) Somatization Subscale. The somatization subscale of the SCL-90R assesses a degree of somatic symptoms experienced by participants. It contains 12 items.[47]

Pittsburgh Sleep Quality Index. This 19-item instrument assesses sleep quality during the previous month across several domains: subjective sleep quality, sleep latency, sleep duration, habitual sleep efficiency, sleep disturbances, use of sleep medication, and daytime dysfunction.[48]

Coping Strategies Questionnaire Revised (CSQ-R). The CSQ-R consists of 27 items relating to how individuals cope with pain. Participants indicate the frequency with which they engage in specific coping activities when experiencing pain, using a 7-category numerical scale ranging from 0 (never do that) to 6 (always do that). It yields 6 subscales reflecting the pain coping strategies that individuals use: diverting attention, catastrophizing, praying and hoping, ignoring pain sensations, reinterpreting pain sensations, and coping self-statements.[49]

Headache Questionnaire. This questionnaire assesses diagnostic criteria for tension-type headache and migraine according to the International Classification of Headache Disorders 3 (beta version). [50]

Headache Impact Test (HIT-6). The HIT-6 evaluates the factors contributing to the burden of headache and consists of 6 items: pain, social functioning, role functioning, vitality, cognitive functioning, and psychological distress.[51] The participant answers each question using responses ranging from "never" to "always." These responses are summed to produce a total HIT-6 score.

7.2 Laboratory Evaluations

7.2.1 Clinical Laboratory Evaluations

In this study, clinical laboratory tests are not needed for the monitoring of safety or the evaluation of study objectives; therefore, none will be performed.

7.2.2 Research Laboratory Evaluations

DNA extracted from whole blood samples will be analyzed for polymorphisms including, but not limited to the *COMT*, *ADRB2*, and *ADRB3* genes. DNA, plasma, and red blood cells will be stored for possible future IRB-approved genetic, cytokine, and COMT activity analyses (Section 14.5).

7.2.3 Specimen Preparation, Handling, and Shipping

Standard operating procedures for specimen collection, preparation, handling, shipping, storage, and analysis will be provided in the MOP. The shipping and storage of all samples will be tracked by a web-based specimen tacking system programmed and maintained by the DCC.

DNA. Whole blood samples for DNA analyses will be collected and stored at -80ºC. Whole blood samples collected at UF and UB will be shipped in batches to UNC. Genomic DNA will be purified from whole blood samples and stored at -80ºC until genotyping is performed and for future use.

Future Use. Plasma and red blood cells for future cytokine and COMT activity analyses will be divided into aliquots and placed in short-term storage at -80ºC at each site. Plasma and red blood cells samples collected at UF and UB will be shipped in batches to UNC.

8 ASSESSMENT OF SAFETY

8.1 Specification of Safety Parameters

Participants in this study will undergo close follow-up. As described in Section 2.3, risks related to participation include: 1) risks related to the administration of propranolol hydrochloride extended-release; 2) risks related to the administration of the ECG; 3) risks associated with questionnaires; 4) risks associated with TMD examinations and psychophysical tests; 5) well-known and anticipated minor risks from venous phlebotomy; and 6) risks related to study participation such as loss of confidentiality of collected data.

The risks described above will be characterized and monitored to protect the safety of participants. In addition, the study has been designed to minimize risks. Participants will be monitored closely for AEs with frequent visits. Adverse events will be managed by the site dentist or site cardiologist depending on the nature of the event.

Examination and close follow-up of parameters capturing participants' overall health will be collected on case report forms. These will be completed at every study visit, and data will be compiled into a prespecified format and reviewed by the DSMB (Section 9).

As part of the study outcomes, study personnel will collect the occurrences of the following expected AEs known to be associated with propranolol hydrochloride extended-release:

- Dizziness or lightheadedness
- Unusual tiredness or fatigue
- Nausea or vomiting
- Upset stomach
- Hands numbness or tingling
- Sleep problems
- Depression

In this study, the following expected events will not be considered reportable AEs:

- Minor bleeding or bruising resulting from the blood draw
- Temporary pain or temporary increases in existing pain elicited during the TMD clinical examination or tests of heat, pressure, or tender points
- Worsening of TMD signs and symptoms during the participant's 12- to 15-week participation

8.1.1 Unanticipated Problems

The Office for Human Research Protections (OHRP) considers unanticipated problems (UPs) involving risks to participants or others to include, in general, any incident, experience, or outcome that meets **all** of the following criteria:

- Unexpected (in terms of nature, severity, or frequency) given (a) the research procedures that are described in the protocol-related documents, such as the IRB-approved research protocol and informed consent document and (b) the characteristics of the participant population being studied;
- Related or possibly related to participation in the research (in the guidance document, possibly related means there is a reasonable possibility that the incident, experience, or outcome may have been caused by the procedures involved in the research);
- Suggests that the research places participants or others at a greater risk of harm (including physical, psychological, economic, or social harm) than was previously known or recognized.

8.1.2 Adverse Events

The International Conference on Harmonisation (ICH) Guideline for Good Clinical Practice (GCP) defines an AE as "any untoward medical occurrence in a patient or clinical investigation

subject administered a pharmaceutical product and which does not necessarily have a causal relationship with this treatment. An adverse event (AE) can therefore be any unfavorable and unintended sign (including an abnormal laboratory finding), symptom, or disease temporally associated with the use of a medicinal (investigational) product, whether or not related to the medicinal (investigational) product."

8.1.3 Serious Adverse Events

A serious adverse event (SAE) is one that meets one or more of the following criteria:

- Results in death
- Is life-threatening (places the participant at immediate risk of death from the event as it occurred)
- Results in inpatient hospitalization or prolongation of existing hospitalization
- Results in a persistent or significant disability or incapacity
- Results in a congenital anomaly or birth defect
- An important medical event that may not result in death, be life-threatening, or require hospitalization may be considered an SAE when, based upon appropriate medical judgment, the event may jeopardize the participant and may require medical or surgical intervention to prevent one of the outcomes listed in this definition.

8.2 Time Period and Frequency for Event Assessment and Follow-Up

Unanticipated problems will be recorded in the data collection system throughout the study.

The PI will record all reportable events with start dates occurring any time after informed consent is obtained until 7 (for nonserious AEs) or 30 days (for SAEs) after the last day of study participation. At each study visit, the investigator will inquire about the occurrence of AE/SAEs since the last visit. Events will be followed for outcome information until the event returns to baseline, resolves, or stabilizes. If the AE does not meet these outcomes within 30 days after discontinuation or the end of the study, the participant will be referred to an appropriate practitioner for continued care.

8.3 Characteristics of Adverse Events

8.3.1 Relationship to Study Drug

The PI will make the determination of the relationship of an AE to the study drug according to the following guidelines:

1. Related (Possible, Probable, Definite)
    a. The event is known to occur with the study intervention.

   b. There is a temporal relationship between the intervention and event onset.
   c. The event abates when the intervention is discontinued.
   d. The event reappears upon a re-challenge with the intervention.

2. Not Related (Unlikely, Not Related)
   a. There is no temporal relationship between the intervention and event onset.
   b. An alternate etiology has been established.

8.3.2 Relationship to Study Procedures

The same procedure as described in Section 8.3.1 for determining the relatedness of an AE to study drug will be used to determine its relatedness to a study procedure.

8.3.3 Expectedness of AEs

The NIDCR Medical Monitor and the Study PI will be responsible for determining whether an AE is expected or unexpected. An AE will be considered unexpected if the nature, severity, or frequency of the event is not consistent with the risk information described in the protocol or the Investigator's Brochure or discussed with the participant during the informed consent process.

8.3.4 Severity of Event

Adverse events will be graded on a scale from 1 to 5 according to the following standards from the National Cancer Institute's *Common Terminology Criteria for Adverse Events* (v 4.0), which may be found in its entirety at http://ctep.cancer.gov/reporting/ctc.html:

- Grade 1 = mild AE
- Grade 2 = moderate AE
- Grade 3 = severe or medically significant AE
- Grade 4 = life-threatening AE
- Grade 5 = death

8.4 Reporting Procedures

8.4.1 Adverse Event Reporting to IRB and NIDCR

Study staff will report AEs on a case report form in the EDC system. Adverse events will be reported to the IRBs and DSMB according to guidelines established by those boards.

8.4.2 Unanticipated Problem Reporting to IRB and NIDCR

Incidents or events that meet the OHRP criteria for unanticipated problems require the creation and completion of an unanticipated problem report form within the EDC system. If an unanticipated problem also meets the criteria for an AE or SAE, it will be reported as an unanticipated problem and an AE or SAE. The OHRP recommends that investigators include the following information when reporting an adverse event, or any other incident, experience, or outcome as an unanticipated problem to the IRB:

- Appropriate identifying information for the research protocol, such as the title, investigator's name, and the IRB project number
- A detailed description of the adverse event, incident, experience, or outcome
- An explanation of the basis for determining that the adverse event, incident, experience, or outcome represents an unanticipated problem
- A description of any changes to the protocol or other corrective actions that have been taken or are proposed in response to the unanticipated problem

To satisfy the requirement for prompt reporting, unanticipated problems will be reported using the following timeline:

- Unanticipated problems that are SAEs will be reported to the IRB and to NIDCR within 1 week of the investigator becoming aware of the event.
- Any other UP will be reported to the IRB and to NIDCR within 2 weeks of the investigator becoming aware of the problem.
- All UPs should be reported to appropriate institutional officials (as required by an institution's written reporting procedures), the supporting agency head (or designee), and OHRP within one month of the IRB's receipt of the report of the problem from the investigator.

All unanticipated problems will be submitted on the UP form within the EDC system to NIDCR's centralized reporting system via Rho Product Safety. However, in cases when the EDC system is not available, the investigator may report the UP by fax or email:

- Product Safety Fax Line (US): 1-888-746-3293
- Product Safety Email: rho_productsafety@rhoworld.com

General questions about UP reporting can be directed to the Rho Product Safety Help Line (available 8:00AM – 5:00PM Eastern Time):

- US: 1-888-746-7231

8.4.3 Serious Adverse Event Reporting to NIDCR

Any AE meeting the specified serious adverse event criteria will be submitted on an SAE form within the EDC system to NIDCR's centralized safety system via Rho Product Safety. However, in cases when the EDC system is not available, the investigator may report the SAE by fax or email. Once submitted, Rho Product Safety will send a confirmation email to the investigator

within 1 business day. The investigator should contact Rho Product Safety if this confirmation is not received. This process applies to both initial and follow-up SAE reports.

SAE Reporting Contact Information:

- Product Safety Fax Line (US): 1-888-746-3293
- Product Safety Email: rho_productsafety@rhoworld.com

General questions about SAE reporting can be directed to the Rho Product Safety Help Line (available 8:00AM – 5:00PM Eastern Time):

- US: 1-888-746-7231

The study clinician will complete a Serious Adverse Event Form and submit within the EDC system (or via fax or email if the EDC system is not available) within the following timelines:

- All deaths and immediately life-threatening events, whether related or unrelated to the study drug, will be recorded on the Serious Adverse Event Form and submitted to Product Safety within 24 hours of site awareness.
- Serious adverse events other than death and immediately life-threatening events, regardless of relationship to the study drug, will be reported within 72 hours of site awareness.

All SAEs will be followed until resolution or stabilization.

Serious adverse events will be reported to the IRBs and DSMB according to guidelines established by those boards.

8.4.4 Reporting Abnormal Clinical Findings

Any clinically significant worsening of a medical condition (other than TMD) established at baseline or the development of any new clinically significant medical condition during the study will be considered an AE and will be reported as described previously.

8.4.5 Reporting of Pregnancy

Pregnancy will be recorded on a case report form within the EDC system if it begins any time during the study. Pregnancy will not be regarded as an SAE unless there is suspicion that the study drug may have interfered with the effectiveness of a contraceptive medication and the event meets the criteria for an unanticipated problem. Results of all positive pregnancy tests will be given to the participant and/or her legally authorized representative, as appropriate.

Participants who become pregnant while participating in the study will be discontinued from the study and referred for appropriate care. If a participant becomes pregnant while receiving the study drug, she will be followed for safety until a pregnancy outcome is reached. If the pregnancy results in an outcome other than a normal birth or elective abortion of a healthy fetus, it will be reported as an SAE.

8.5 Halting Rules

See Section 4.4.6, Suspension or Termination of the Study.

9 STUDY OVERSIGHT

In addition to the PI's responsibility for oversight, safety oversight will be under the direction of a DSMB convened by NIDCR. The DSMB is composed of members with expertise in pain management. The DSMB will meet periodically or as needed to assess safety and efficacy data, study progress, and data integrity for the study. If safety concerns arise, more frequent meetings may be held. The DSMB will operate under the rules of an NIDCR-approved charter that will be written at the organizational meeting of the DSMB. At this time, most data elements that the DSMB needs to assess will be clearly defined. The DSMB will provide recommendations to the NIDCR. The PI will be responsible for responding to inquiries and recommendations made by the DSMB.

10 CLINICAL MONITORING

Clinical site monitoring is conducted to ensure that the rights of human participants are protected, that the study is implemented in accordance with the protocol and/or other operating procedures, and that the quality and integrity of study data and data collection methods are maintained. Monitoring for this study will be performed by NIDCR's Clinical Research Operations and Management Support (CROMS) contractor. The monitor will evaluate study processes and documentation based on NIDCR standards and the ICH E6: GCP guidelines.

Details of clinical site monitoring will be documented in a Clinical Monitoring Plan (CMP) developed by the CROMS contractor, in collaboration with the NIDCR Office of Clinical Trials and Operations Management (OCTOM) and the NIDCR Program Official. The CMP will specify the frequency of monitoring, monitoring procedures, the level of clinical site monitoring activities (e.g., the percentage of participant data to be reviewed), and the distribution of monitoring reports. Some monitoring activities may be performed remotely, while others will take place at the study site(s). Staff from the CROMS contractor will conduct monitoring activities and provide reports of the findings and associated action items in accordance with the details described in the CMP. Documentation of monitoring activities and findings will be provided to the site study team, the study PIs, OCTOM, and the NIDCR. The NIDCR reserves the right to conduct independent audits as necessary.

11 STATISTICAL CONSIDERATIONS

11.1 Study Hypotheses

11.1.1 Primary Hypothesis

We hypothesize that among this study population of participants with TMD, the net change in the mean pain index at Visit 4 will be greater in the propranolol-treated arm than in the placebo-treated arm. Net change is defined as the difference between the participant's mean pain index at Visit 1 and each subsequent visit. Efficacy of propranolol is defined as the difference between the propranolol and placebo arms in the mean net change. Figure 2 uses hypothetical data to illustrate how the primary hypothesis is restricted to efficacy at Visit 4 (ΔV4).

Figure 2. Hypothetical Data Illustrating the Primary Hypothesis

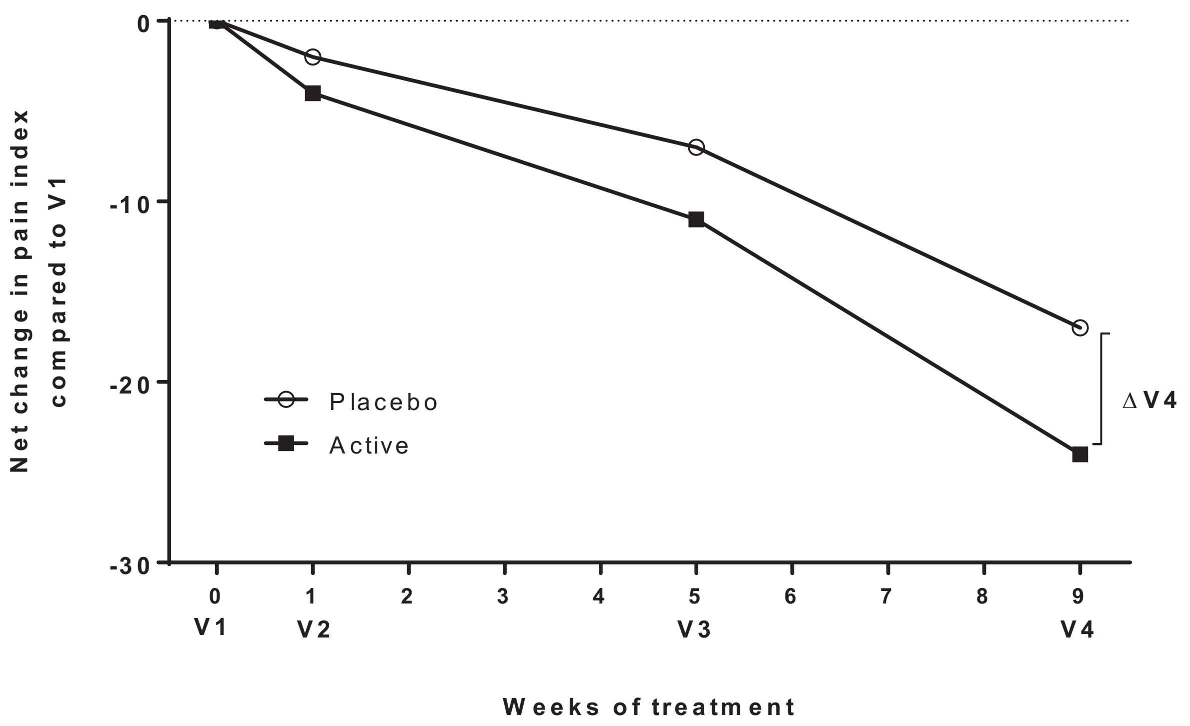

11.1.2 Secondary Hypothesis

- We hypothesize that among this study population of participants with TMD, the efficacy of propranolol will vary according to the number of LPS haplotypes of the *COMT* gene.
- We hypothesize that, compared with participants in the placebo-treated arm, participants in the propranolol-treated arm will have more favorable secondary endpoints, namely: (1) reduction in additional ratings of pain; (2) reduction in examiner-assessed levels of

pain sensitivity (pressure and heat pain sensitivity); (3) better participant ratings of physical function, emotional function, and global improvement; and (4) less frequent use of rescue medications and over-the-counter medications for pain.

11.1.3 Exploratory Hypotheses

- We hypothesize that efficacy of propranolol will vary according to genetic variants including, but not limited to the *COMT* gene promoter and the *ADRB2* and *ADRB3* genes.
- We hypothesize that efficacy of propranolol will vary according to phenotypic characteristics.

11.2 Sample Size Considerations

This study plans to randomize 200 participants, which will provide 94% power (alpha = 0.05) to detect a difference between a 27% reduction in pain index in the propranolol arm and a 9% reduction in the placebo arm (primary hypothesis). Assuming haplotype distributions and within-haplotype treatment effects that are similar to those observed in the pilot study, the same sample size would provide 61% power (alpha = 0.05) to detect an interaction between haplotype and treatment group (secondary hypothesis). The assumptions for these power calculations are shown in the following table. Power calculations were made using the GLMPOWER procedure in SAS, which assumes a data structure of one observation per person. This is a simplification of the situation that will occur with the linear mixed model described in Section 11.4.1 in which efficacy will be evaluated using a data structure in which most participants will have three observations per person. The simplified power calculations are shown here because conventional software for power calculations does not deal with the more complex situation of linear mixed models.

	Primary Hypothesis		Secondary Hypothesis					
COMT haplotype	All haplotypes		0 LPS copies		1 LPS copy		2 LPS copies	
Treatment arm	Drug	Placebo	Drug	Placebo	Drug	Placebo	Drug	Placebo
% of participants (N = 200)	50%	50%	15%	15%	25%	25%	10%	10%
Mean baseline pain index	3000	3000	3000	3000	3000	3000	3000	3000
Net change	-810	-270	-1350	-270	-810	-270	-270	-270
Percentage change	-27	-9	-45	-9	-27	-9	-9	-9

∗ LPS stands for low pain sensitive, one of the major haplotypes of *COMT*. The standard deviation for the mean baseline pain index is estimated to be 1190. The net change takes into account a 10% lost-to-follow-up rate and imputation using the last observation carried forward approach.

11.3 Analysis Samples

11.3.1 Intention-To-Treat Sample

Consistent with the primary statistical method that will use a linear mixed model (also called "mixed model repeated measures" model), the intention-to-treat sample is defined as all study participants who have at least one valid measurement of the primary endpoint at Visit 2 or later. A "valid" assessment for the primary endpoint is defined as at least 4 daily reports of pain intensity and duration collected in the 7-day period prior to the visit (Section 2.5.1). Treatment group allocation will be defined as the study arm into which the subject was randomized regardless of the actual study treatment received. By necessity, the intention-to-treat sample excludes participants who provide no data for the primary endpoint after randomization. Note that follow-up assessments will be included in the intention-to-treat analysis when sufficient diary data are collected at the follow-up assessment, even if there were protocol violations. Likewise, the intention-to-treat sample will include all available follow-up assessments from any discontinued participants.

11.3.2 Per-Protocol Sample

Secondary statistical analysis of the primary hypothesis will be in the spirit of per-protocol analysis. The per-protocol sample will be a subset of the intention-to-treat sample, excluding any assessments made at or after a visit at which the participant is found to have any protocol deviation listed in Section 5.8. In addition, all assessments for participants whose compliance with study medication is less than 60% over all study visits will be excluded from per-protocol analyses.

11.3.3 Safety Sample

The safety sample will include all randomized participants who received at least one dose of study medication. Participants in the safety sample will be analyzed with the treatment group according to the medication they actually received, regardless of their randomized assignment.

11.4 Analyses

Complete details of all statistical analyses will be provided in the Statistical Analysis Plan, which will be completed prior to database lock and analysis. Below is an overview of the analyses for the primary and secondary hypotheses.

11.4.1 Analysis of the Primary Hypothesis

The primary statistical analysis to evaluate the primary hypothesis will use all data from the intention-to-treat analytical sample in a linear mixed model. During the analysis of the primary hypothesis, treatment allocation will be masked (i.e., groups will be labeled A and B). Treatment allocation will be unmasked for further analyses.

The dependent variable will be the participant's change in the weekly pain index, computed as the weekly pain index at the follow-up visit (i.e., at Visit 2, Visit 3, or Visit 4) minus the Visit 1 weekly pain index. Explanatory variables will comprise fixed factors and a continuous covariate. The fixed factors will include the following: (a) treatment allocation (two categories) (b) visit sequence (three categories); (c) interaction of treatment allocation and visit sequence (two dummy variables); and (d) covariates of study site (three categories), sex (two categories) and race/ethnicity (probably three categories: non-Hispanic White, non-Hispanic Black, and other/multiple racial or ethnic groups – the final number of conditions will be dictated by the frequency distribution of race/ethnicity in the study sample). The baseline score collected at Visit 1 will be the continuous covariate, modeled as a fixed effect The study participant will be modeled as a random factor.

In a linear mixed model containing all predictor variables including the interaction terms for visit and treatment allocation, the primary treatment effect will be tested at Visit 4. The null hypothesis of no treatment effect will be rejected if the P-value at Visit 4 is ≤ 0.05. For descriptive purposes (and regardless of the P-value for efficacy at Visit 4), efficacy estimates at each visit (2, 3 and 4) will be described in terms of adjusted means and 95% confidence intervals.

If the propranolol treatment arm has a greater reduction in the weekly pain index than the placebo arm at Visit 4, a threshold of "responder analysis" will be undertaken to estimate the odds of at least a 30% reduction, relative to Visit 1, in the pain index at Visit 4. Likewise, another threshold of responder analysis will describe the odds of experiencing at least a 50% reduction. These estimates will be calculated from a logistic model in which the dependent variable is a binary variable that dichotomizes the change score at the appropriate threshold (i.e., ≥ 30% or ≥ 50%).

Secondary analyses of the primary hypothesis will be conducted as described above, but using the per-protocol sample.

Stratified analysis in the intention-to-treat sample will estimate treatment effects separately for the two sexes and for the main racial-ethnic groups. The analysis will be descriptive, reporting means and 95% confidence intervals. There will be no hypothesis tests of efficacy within demographic subgroups because of the expected small numbers of males and minorities.

11.4.2 Analysis of the Secondary Hypotheses

To test the secondary hypothesis of gene-by-treatment interaction, the linear mixed model described for the primary analysis will be extended by adding three sets of predictor variables: (1) the number of *COMT* LPS haplotypes (0, 1, or 2) will be modeled as a fixed effect, either as a continuous variable, or as a 3-level categorical variable (i.e., two dummy variables), with the decision based on the approach that optimizes model fit, as determine by Akaike's Information Criterion; (2) two-way interactions between haplotype effect and treatment allocation and between haplotype effect and visit sequence; and (3) a three-way interaction term between the

haplotype effect, treatment allocation, and visit sequence. The gene-by-treatment interaction at V4 is the interaction of interest.

For descriptive purposes, an analogous model will generate efficacy estimates according to *COMT* $Val^{158}Met$ genotype as the genetic marker instead of the number of LPS haplotype. In an analogous manner, the genotype will be coded either as a continuous or categorical variable based on optimal model fit. Efficacy estimates will be calculated for descriptive purposes only; there will be no hypothesis tests for the interaction terms.

Another descriptive set of secondary analyses will adapt the approach for analysis of the LPS haplotype, restricting the model to non-Hispanic whites. This is consistent with the approach of some genetic researchers who advocate racial-group restriction as a preferable analytic method to control for population stratification.

Linear mixed models will be used to evaluate effects of treatment group allocation on secondary efficacy variables (see Statistical Analysis Plan for full details).

Linear regression or other appropriate statistical modeling will evaluate the effects of treatment on the number of days in which rescue medications for TMD-related pain reportedly were used and on the number of days in which over-the-counter analgesics (namely, NSAIDs, acetaminophen, and aspirin) reportedly were used for treatment of any type of pain.

See the Statistical Analysis Plan for more details on these secondary analyses.

11.4.3 Analysis of the Exploratory Hypotheses

The exploratory hypotheses will extend the analytical procedures described above. In summary:

- The linear mixed model described for the secondary hypothesis will be modified by specifying genetic variants including, but not limited to the *COMT* gene promoter and the *ADRB2* and *ADRB3* genes.
- Other phenotypes will be evaluated as potential treatment effect modifiers using a similar strategy in which linear mixed models will test for phenotype-by-treatment interactions. Each phenotype will be categorized based on characteristics assessed either at V0 or V1. The phenotypes are: body mass index; smoking; migraine headache; tenderness to body palpation; catastrophizing subscale of the CSQ-R questionnaire; self-rated general health from the SF-12v2 questionnaire; anxiety and depression subscales from the HADS questionnaire; sleep quality from the PSQI questionnaire; and subjects' rating of confidence in treatment outcome. Full details are provided in the Statistical Analysis Plan.

The exploratory analysis will further explore the results, insights, or new questions that may arise during the study's analyses and will not be limited to the exploratory analyses listed above.

11.5 Planned Interim Analyses

No interim analyses are planned.

12 SOURCE DOCUMENTS AND ACCESS TO SOURCE DATA AND DOCUMENTS

Source data are all information, original records of clinical findings, observations, or other activities in a clinical trial necessary for the reconstruction and evaluation of the trial. Examples of these original documents and data records include, but are not limited to, hospital records, clinical and office charts, laboratory notes, memoranda, participants' memory aid or evaluation checklists, pharmacy dispensing records, recorded data from automated instruments, recorded audio tapes of counseling sessions, copies or transcriptions certified after verification as being accurate and complete, microfiches, photographic negatives, microfilm or magnetic media, x-rays, and participant files and records kept at the pharmacy, at the laboratories, and medico-technical departments involved in the clinical trial. It is acceptable to use case report forms as source documents. For this study, some clinical examination and questionnaire data may be recorded directly from the participant onto the Teleform case report forms (paper), with the case report form being the source document.

Each clinical site will maintain appropriate medical and research records for this trial in compliance with Section 4.9 of ICH E6 and regulatory and institutional requirements for the protection of confidentiality of participants. As part of participating in an NIDCR-funded study, each site will permit authorized representatives of the NIDCR, including CROMS clinical monitors, and regulatory agencies to examine (and when required by applicable law, to copy) clinical records for the purposes of quality assurance reviews, audits, and evaluation of the study's safety and progress.

13 QUALITY CONTROL AND QUALITY ASSURANCE

Quality control is the ongoing, concurrent review of data collection forms for completion and logic. Quality assurance is a comprehensive, retrospective review of all components of research records to assess adherence to protocol, standard operation procedures, and regulations and to evaluate the accuracy of the records. Quality management is the process of assessing the quality of processes within a system and encompasses quality assurance and quality control.

The quality management program will include, but will not be limited to, the following:

- A study start-up list of tasks that must be completed and approved by the NIDCR program officer prior to the first participant visit
- Training of staff on the protocol, study procedures, and use of the data collection forms and systems
- Documentation and tracking of training for each staff member
- A Clinical Monitoring Plan that describes periodic clinic visits by monitors to ensure that the rights of human participants are protected, the study is implemented in accordance with the protocol, and the integrity of study data is maintained

- A Data Management Plan that describes procedures for data entry and completion, data security, and data quality control and validation

14 PROTECTION OF HUMAN PARTICIPANTS

14.1 Institutional Review Board

Each participating institution will provide for the review and approval of this protocol and the associated informed consent documents and recruitment materials by an appropriate IRB registered with the OHRP. Any amendments to the protocol or consent materials will also be approved by the IRB before they are placed into use.

14.2 Informed Consent Process

Written documentation of informed consent will be obtained prior to an individual's participation in the study. An IRB-approved consent form describing in detail the study interventions, procedures, and risks will be given to the participant. The participant will be asked to read and review the document, and upon reviewing the document, the investigator or designee will explain the research study to the participant and answer any questions that may arise. The participant will have the opportunity to discuss the study with others, such as family members, or to take additional time before agreeing to participate. A participant may withdraw consent at any time throughout the course of the trial. A copy of the informed consent document will be given to the participant for her records.

14.3 Exclusion of Special Populations

This protocol does not exclude any special populations.

14.4 Participant Confidentiality

Participant confidentiality will be strictly held in trust by the participating investigators, their staff, the data coordinating center, and the NIDCR and its agents. This confidentiality will be extended to cover testing of biological samples and genetic tests in addition to the clinical information relating to study participants.

The study protocol, documentation, data, and all other information generated will be held in strict confidence. No information concerning the study or the data will be released to any unauthorized third party without prior written approval of the NIDCR program official.

The clinical monitor or other authorized representatives of the NIDCR may inspect all documents and records required to be maintained by the investigator, including but not limited to medical records (office, clinic, or hospital) and pharmacy records for the participants in this study. The clinical study site will permit access to such records.

Research information about the participants will be collected on either paper or electronic forms that will contain a study identification number rather than a name or any other information that could be used to identify the participant. Only the study staff will have access to the list that links the name and identification number. All paper research forms will be kept under lock and key.

14.5 Future Use of Stored Specimens

Biological specimens will be stored indefinitely at one or both of the clinical sites for participants who provide consent. The consent form will allow participants to choose whether to have their samples stored indefinitely for future analyses. Any new use of study specimens, i.e., uses not described in this protocol, will require IRB review and approval. Stored specimens will be identifiable by participant identification number. The link between the participant's name and the identification number will be maintained and stored confidentially by the study investigators. Any loss or unintentional destruction of the samples will be reported to the IRBs. If it is determined that no additional investigations are planned on the study specimens, the specimens will be disposed of in accordance with environmental protection laws, local regulations, and guidelines of the US federal government.

The consent for future use of specimens will include the possibility that genetic information may be obtained from the specimens and shared with other researchers via an NIH database. In accordance with the NIH Genomic Data Sharing Policy, an IRB will need to ensure that data submission is consistent with the informed consent provided by the research participant.

14.6 Data Handling and Record Keeping

All source documents will be completed in a neat, legible manner to ensure accurate interpretation of data. Black or blue ink is required to ensure clarity of reproduced copies. When making corrections, the original entry will be crossed out with a single line, and the correction will be initialed and dated. No entries will be erased or overwritten. Data reported on case report forms will be consistent with the source documents or the discrepancies will be explained and documented. For this study, case report forms (paper or electronic) may serve as the source document for the collection of some types of data, such as questionnaire data or clinical examination findings.

14.7 Data Management Responsibilities

The investigator will ensure the accuracy, completeness, legibility, and timeliness of the data collection. During the study, the investigator will maintain complete and accurate documentation for the study. All source documents and laboratory reports will be reviewed by the clinical team and data entry staff, who will ensure that all recorded information is accurate and complete. The data coordinating center will be responsible for data management, data quality review, and data analyses.

14.8 Data Capture Methods

Study staff will complete case report forms online via a web-based EDC system that has been validated and is compliant with Title 21 CFR Part 11. The data will be stored remotely at a central database managed by the data coordinating center. Data quality will be ensured through the data collection system's continuous monitoring of data and real-time detection and correction of errors. All elements of data entry (i.e., time, date, verbatim text, and the name of the person performing the data entry) will be recorded in an electronic audit trail to allow all changes in the database to be monitored and maintained in accordance with federal regulations.

14.9 Types of Data

Clinical examination findings, clinical test results, demographic and medical history data, outcome measures obtained from questionnaires, and genetic data will be collected in this study.

14.10 Timing of Reports

The data coordinating center will post study progress reports (i.e., screening, enrollment, and study progress reports) to the study website periodically throughout the study period, as determined by the NIDCR program official and the PI. The data coordinating center will also post data management reports that contain measures of data quality, such as the number of outstanding data queries and data completion rates. Study reports will be provided to the DSMB and IRBs prior to their periodic meetings. No interim statistical analyses are planned.

14.11 Study Records Retention

Records will be maintained for at least 3 years from the date that the final financial status report is submitted to the NIH. No records will be destroyed without the written consent of the NIH.

14.12 Protocol Deviations

A protocol deviation is any noncompliance with the clinical trial protocol, GCP, or the MOP. The noncompliance may be on the part of the participant, the investigator, or the study site staff. All protocol deviations will be promptly reported within the EDC system and to the NIDCR program official. Protocol deviations will also be reported to the IRBs and the DSMB according to their guidelines. As a result of deviations, corrective actions will be developed by the site(s) and implemented promptly.

15 PUBLICATION/DATA SHARING POLICY

This study will comply with the NIH Public Access Policy, which ensures that the public has access to the published results of NIH-funded research. It requires scientists to submit final peer-reviewed journal manuscripts that arise from NIH funds to the digital archive PubMed Central upon acceptance for publication.

This study will comply with the NIH Data Sharing Policy, which ensures that data are as widely and freely available as possible while safeguarding the privacy of participants, and protecting confidential and proprietary data. Genetic and non-genetic data collected in this study will be stored for use by other researchers. Datasets will not contain any personal identifying information, and an IRB will need to determine whether the data may be released to researchers.

The International Committee of Medical Journal Editors (ICMJE) member journals have adopted a clinical trials registration policy as a condition for publication. The ICMJE defines a clinical trial as any research project that prospectively assigns human participants to intervention or concurrent comparison or control groups to study the cause-and-effect relationship between a medical intervention and a health outcome. Medical interventions include drugs, surgical procedures, devices, behavioral treatments, process-of-care changes, and the like. Health outcomes include any biomedical or health-related measures obtained in patients or participants, including pharmacokinetic measures and AEs. The ICMJE policy requires that all clinical trials be registered in a public trials registry such as ClinicalTrials.gov, which is sponsored by the National Library of Medicine. Other biomedical journals are considering adopting similar policies. For interventional clinical trials performed under NIDCR grants and cooperative agreements, it is the grantee’s responsibility to register the trial in an acceptable registry, so the research results may be considered for publication in ICMJE member journals. The ICMJE does not review specific studies to determine whether registration is necessary; instead, the committee recommends that researchers who have questions about the need to register err on the side of registration or consult the editorial office of the journal in which they wish to publish.

U.S. Public Law 110-85 (Food and Drug Administration Amendments Act of 2007 or FDAAA), Title VIII, Section 801 mandates that a "responsible party" (i.e., the sponsor or designated principal investigator) register and report results of certain "applicable clinical trials:"

Trials of Drugs and Biologics: Controlled, clinical investigations, other than Phase I investigations, of a product subject to FDA regulation;

Trials of Devices: Controlled trials with health outcomes of a product subject to FDA regulation (other than small feasibility studies) and pediatric post market surveillance studies.

NIH grantees must take specific steps to ensure compliance with NIH implementation of FDAAA.

16 LITERATURE REFERENCES

1. De Leeuw R, editor. Orofacial pain: guidelines for assessment, diagnosis, and management. 4th ed. Chicago: Quintessence Publishing Co, Inc; 2008.

2. Machado LP, Nery Cde G, Leles CR, Nery MB, Okeson JP. The prevalence of clinical diagnostic groups in patients with temporomandibular disorders. Cranio. 2009;27(3):194-9.

3. Dworkin SF, LeResche L. Research diagnostic criteria for temporomandibular disorders: review, criteria, examinations and specifications, critique. J Craniomandib Disord. 1992;6(4):301-55.

4. Dworkin SF, Huggins KH, LeResche L, Von Korff M, Howard J, Truelove E, Sommers E. Epidemiology of signs and symptoms in temporomandibular disorders: clinical signs in cases and controls. J Am Dent Assoc. 1990;120(3):273-81.

5. Dworkin SF, LeResche L. Temporomandibular disorder pain: Epidemiologic data. APS Bulletin. 1993;April/May:12.

6. Dahlstrom L, Carlsson GE. Temporomandibular disorders and oral health-related quality of life. A systematic review. Acta Odontol Scand.68(2):80-5.

7. Von Korff M, Ormel J, Keefe FJ, Dworkin SF. Grading the severity of chronic pain. Pain. 1992;50(2):133-49.

8. Chen H, Slade G, Lim PF, Miller V, Maixner W, Diatchenko L. Relationship between temporomandibular disorders, widespread palpation tenderness, and multiple pain conditions: a case-control study. J Pain. 2012;13(10):1016-27.

9. Plesh O, Adams SH, Gansky SA. Temporomandibular joint and muscle disorder-type pain and comorbid pains in a national US sample. J Orofac Pain. 2011;25(3):190-8.

10. Hersh EV, Balasubramaniam R, Pinto A. Pharmacologic management of temporomandibular disorders. Oral Maxillofac Surg Clin North Am. 2008;20(2):197-210, vi.

11. List T, Axelsson S. Management of TMD: evidence from systematic reviews and meta-analyses. J Oral Rehabil. 2010;37(6):430-51.

12. Evaskus DS, Laskin DM. A biochemical measure of stress in patients with myofascial pain-dysfunction syndrome. J Dent Res. 1972;51(5):1464-6.

13. Perry F, Heller PH, Kamiya J, Levine JD. Altered autonomic function in patients with arthritis or with chronic myofascial pain. Pain. 1989;39(1):77-84.

14. Torpy DJ, Papanicolaou DA, Lotsikas AJ, Wilder RL, Chrousos GP, Pillemer SR. Responses of the sympathetic nervous system and the hypothalamic-pituitary-adrenal axis to interleukin-6: a pilot study in fibromyalgia. Arthritis Rheum. 2000;43(4):872-80.

15. Khasar SG, McCarter G, Levine JD. Epinephrine produces a beta-adrenergic receptor-mediated mechanical hyperalgesia and in vitro sensitization of rat nociceptors. J Neurophysiol. 1999;81(3):1104-12.

16. Light KC, Bragdon EE, Grewen KM, Brownley KA, Girdler SS, Maixner W. Adrenergic dysregulation and pain with and without acute beta-blockade in women with fibromyalgia and temporomandibular disorder. J Pain. 2009;10(5):542-52.

17. Wood PB, Kablinger AS, Caldito GS. Open trial of pindolol in the treatment of fibromyalgia. Ann Pharmacother. 2005;39(11):1812-6.

18. Rodrigues LL, Oliveira MC, Pelegrini-da-Silva A, de Arruda Veiga MC, Parada CA, Tambeli CH. Peripheral sympathetic component of the temporomandibular joint inflammatory pain in rats. J Pain. 2006;7(12):929-36.

19. Keller S, Frishman WH. Neuropsychiatric effects of cardiovascular drug therapy. Cardiol Rev. 2003;11(2):73-93.

20. Tchivileva IE, Lim PF, Smith SB, Slade GD, Diatchenko L, McLean SA, Maixner W. Effect of catechol-O-methyltransferase polymorphism on response to propranolol therapy in chronic musculoskeletal pain: a randomized, double-blind, placebo-controlled, crossover pilot study. Pharmacogenet Genomics. 2010;20(4):239-48.

21. Mannisto PT, Kaakkola S. Catechol-O-methyltransferase (COMT): biochemistry, molecular biology, pharmacology, and clinical efficacy of the new selective COMT inhibitors. Pharmacol Rev. 1999;51(4):593-628.

22. Marbach JJ, Levitt M. Erythrocyte catechol-O-methyltransferase activity in facial pain patients. J Dent Res. 1976;55(4):711.

23. Nackley AG, Shabalina SA, Chivileva IE, Satterfield KS, Korchynskyy O, Maixner W, Diatchenko L, editors. Common human catechol-O-methyltransferase haplotypes modulate RNA stability, protein expression, and enzymatic activity. American Society for Human Genetics; 2005; Salt Lake City, Utah.

24. Lotta T, Vidgren J, Tilgmann C, Ulmanen I, Melen K, Julkunen I, Taskinen J. Kinetics of human soluble and membrane-bound catechol O-methyltransferase: a revised mechanism and description of the thermolabile variant of the enzyme. Biochemistry. 1995;34(13):4202-10.

25. Gursoy S, Erdal E, Herken H, Madenci E, Alasehirli B, Erdal N. Significance of catechol-O-methyltransferase gene polymorphism in fibromyalgia syndrome. Rheumatol Int. 2003;23(3):104-7.

26. Erdal ME, Herken H, Yilmaz M, Bayazit YA. Significance of the catechol-O-methyltransferase gene polymorphism in migraine. Brain Res Mol Brain Res. 2001;94(1-2):193-6.

27. Diatchenko L, Slade GD, Nackley AG, Bhalang K, Sigurdsson A, Belfer I, Goldman D, Xu K, Shabalina SA, Shagin D, Max MB, Makarov SS, Maixner W. Genetic basis for individual

variations in pain perception and the development of a chronic pain condition. Hum Mol Genet. 2005;14(1):135-43.

28. Zubieta JK, Heitzeg MM, Smith YR, Bueller JA, Xu K, Xu Y, Koeppe RA, Stohler CS, Goldman D. COMT val158met genotype affects mu-opioid neurotransmitter responses to a pain stressor. Science. 2003;299(5610):1240-3.

29. Rakvag TT, Klepstad P, Baar C, Kvam TM, Dale O, Kaasa S, Krokan HE, Skorpen F. The Val158Met polymorphism of the human catechol-O-methyltransferase (COMT) gene may influence morphine requirements in cancer pain patients. Pain. 2005;116(1-2):73-8.

30. Diatchenko L, Nackley AG, Slade GD, Bhalang K, Belfer I, Max MB, Goldman D, Maixner W. Catechol-O-methyltransferase gene polymorphisms are associated with multiple pain-evoking stimuli. Pain. 2006;125(3):216-24.

31. Nackley AG, Shabalina SA, Tchivileva IE, Satterfield K, Korchynskyi O, Makarov SS, Maixner W, Diatchenko L. Human catechol-O-methyltransferase haplotypes modulate protein expression by altering mRNA secondary structure. Science. 2006;314(5807):1930-3.

32. Nackley AG, Tan KS, Fecho K, Flood P, Diatchenko L, Maixner W. Catechol-O-methyltransferase inhibition increases pain sensitivity through activation of both beta2- and beta3-adrenergic receptors. Pain. 2007;128(3):199-208.

33. Dworkin RH, Turk DC, Farrar JT, Haythornthwaite JA, Jensen MP, Katz NP, Kerns RD, Stucki G, Allen RR, Bellamy N, Carr DB, Chandler J, Cowan P, Dionne R, Galer BS, Hertz S, Jadad AR, Kramer LD, Manning DC, Martin S, McCormick CG, McDermott MP, McGrath P, Quessy S, Rappaport BA, Robbins W, Robinson JP, Rothman M, Royal MA, Simon L, Stauffer JW, Stein W, Tollett J, Wernicke J, Witter J. Core outcome measures for chronic pain clinical trials: IMMPACT recommendations. Pain. 2005;113(1-2):9-19.

34. Schulz KF, Altman DG, Moher D. CONSORT 2010 statement: updated guidelines for reporting parallel group randomized trials. Ann Intern Med. 2010;152(11):726-32.

35. Manfredini D, Arveda N, Guarda-Nardini L, Segu M, Collesano V. Distribution of diagnoses in a population of patients with temporomandibular disorders. Oral Surg Oral Med Oral Pathol Oral Radiol. 2012;114(5):e35-41.

36. Schiffman E, Ohrbach R, Truelove E, Look J, Anderson G, Goulet J-P, List T, Svensson P, Gonzalez Y, Lobbezoo F, Michelotti A, Brooks SL, Ceusters W, Drangsholt M, Ettlin D, Gaul C, Goldberg L, Haythornthwaite J, Hollender L, Jensen R, John MT, deLaat A, deLeeuw R, Maixner W, van der Meulen M, Murray GM, Nixdorf DR, Palla S, Petersson A, Pionchon P, Smith B, Visscher CM, Zakrzewska J, Dworkin SF. Diagnostic Criteria for Temporomandibular Disorders (DC/TMD) for Clinical and Research Applications: Recommendations of the International RDC/TMD Consortium Network and Orofacial Pain Special Interest Group. J Oral Facial Pain Headache. 2014;28(1):6-27.

37. Wolfe F, Smythe HA, Yunus MB, Bennett RM, Bombardier C, Goldenberg DL, Tugwell P, Campbell SM, Abeles M, Clark P, et al. The American College of Rheumatology 1990 Criteria

for the Classification of Fibromyalgia. Report of the Multicenter Criteria Committee. Arthritis Rheum. 1990;33(2):160-72.

38. Babor TF, Higgins-Biddle JC, Saunders JB, Monteiro MG. The Alcohol Use Disorders Identification Test Guidelines for Use in Primary Care, 2nd edition. WHO/MSD/MSB/01.6a, World Health Organization, Geneva, 2001.

39. Melzack R. The McGill Pain Questionnaire: major properties and scoring methods. Pain. 1975;1(3):277-99.

40. Underwood MR, Barnett AG, Vickers MR. Evaluation of two time-specific back pain outcome measures. Spine (Phila Pa 1976). 1999;24(11):1104-12.

41. Ware J, Jr., Kosinski M, Keller SD. A 12-Item Short-Form Health Survey: construction of scales and preliminary tests of reliability and validity. Med Care. 1996;34(3):220-33.

42. Guy W. ECDEU assessment manual for psychopharmacology. In: U.S. Department of Health EaW, editor. Rockville, MD1976. p. 217-22.

43. Ohrbach R, Granger C, List T, Dworkin S. Preliminary development and validation of the Jaw Functional Limitation Scale. Community Dent Oral Epidemiol. 2008;36(3):228-36.

44. Ohrbach R, Larsson P, List T. The jaw functional limitation scale: development, reliability, and validity of 8-item and 20-item versions. J Orofac Pain. 2008;22(3):219-30.

45. Cohen S, Kamarck T, Mermelstein R. A global measure of perceived stress. J Health Soc Behav. 1983;24(4):385-96.

46. Zigmond AS, Snaith RP. The hospital anxiety and depression scale. Acta Psychiatr Scand. 1983;67(6):361-70.

47. Krogstad BS, Jokstad A, Dahl BL, Soboleva U. Somatic complaints, psychologic distress, and treatment outcome in two groups of TMD patients, one previously subjected to whiplash injury. J Orofac Pain. 1998;12(2):136-44.

48. Buysse DJ, Reynolds CF, 3rd, Monk TH, Berman SR, Kupfer DJ. The Pittsburgh Sleep Quality Index: a new instrument for psychiatric practice and research. Psychiatry Res. 1989;28(2):193-213.

49. Rosenstiel AK, Keefe FJ. The use of coping strategies in chronic low back pain patients: relationship to patient characteristics and current adjustment. Pain. 1983;17(1):33-44.

50. The International Classification of Headache Disorders, 3rd edition (beta version). Cephalalgia. 2013;33(9):629-808.

51. Kosinski M, Bayliss MS, Bjorner JB, Ware JE, Jr., Garber WH, Batenhorst A, Cady R, Dahlof CG, Dowson A, Tepper S. A six-item short-form survey for measuring headache impact: the HIT-6. Qual Life Res. 2003;12(8):963-74.

APPENDIX A: SCHEDULE OF EVENTS

Study Phase:	Pre-screening[a]	Screening and Baseline	Randomization and Treatment (1 week titration, 8 weeks maintenance, and 1 week taper)				Follow-Up
**Clinic Visit:**		**V0**	**V1[b]**	**V2**	**V3**	**V4**	**V5**
**Study Day:**	**≤ 28 days prior to V0**	**7 to 21 days prior to V1**	**0**	**7 (+ 3)**	**35 (± 7)**	**63 (+ 6)**	**77 (+ 7)**
**Procedure**							
Contact Information	x						
Prescreening Interview Script	x						
Informed Consent[c]		x					
Eligibility Review	x	x	x				
AUDIT Questionnaire		x					
Smoking Questionnaire		x					x
Alcohol Consumption per week			x	x	x	x	x
Demographic Information		x					
Medical History and Review	x	x	x	x	x	x	x
Concomitant Medications		x	x	x	x	x	x
Concomitant Therapies		x	x	x	x	x	x
Dispense New Daily Symptom Diaries		x	x	x	x	x	
Adverse Event Review			x	x	x	x	x
Collect Daily Symptom Diaries			x	x	x	x	x
Assess Compliance with Daily Symptom Diaries			x	x	x	x	x
Collect Confidence in Treatment Outcome			x				
Randomization			x				
Dispense Study Drug			x[d]	x	x	x	
Collect Study Drug Container(s)				x	x	x	x
Assess Compliance with Study Drug				x	x	x	x

Study Phase:	Pre-screening[a]	Screening and Baseline	Randomization and Treatment (1 week titration, 8 weeks maintenance, and 1 week taper)				Follow-Up
Clinic Visit:		V0	V1[b]	V2	V3	V4	V5
Study Day:	≤ 28 days prior to V0	7 to 21 days prior to V1	0	7 (+ 3)	35 (± 7)	63 (+ 6)	77 (+ 7)
**Clinical Examinations and Tests**							
Physical Measurements		x					
Vital Signs		x	x	x	x	x	x
Urine Pregnancy Test (females)		x	x	x	x	x	x
Manual Tender Point Exam		x					
TMD Examination		x			x	x	
Heat and Pressure Pain Tests		x			x	x	
12-Lead ECG		x					
Blood Draw			x			x	
Schedule Next Visit	x	x	x	x	x	x	
**Outcome Measure Questionnaires**							
Symptom Inventory		x	x	x	x	x	x
Fibromyalgia Questionnaire		x					
Headache Questionnaire[e]			x				
SF-McGill Pain Questionnaire		x		x	x	x	x
Graded Chronic Pain Scale[e]			x		x	x	
SF-12 Health Survey v2[e]			x		x	x	
Jaw Functional Limitation Scale[e]			x		x	x	
Perceived Stress Scale[e]			x		x	x	
Hospital Anxiety and Depression Scale[e]			x		x	x	
Pittsburgh Sleep Quality Index[e]			x		x	x	
Headache Impact Test (HIT-6)[e]			x		x	x	
SCL-90R Somatization Scale[e]			x		x	x	
Coping Strategies Questionnaire-Revised[e]			x				

Study Phase:	Pre-screening[a]	Screening and Baseline	Randomization and Treatment (1 week titration, 8 weeks maintenance, and 1 week taper)				Follow-Up
Clinic Visit:		V0	V1[b]	V2	V3	V4	V5
Study Day:	≤ 28 days prior to V0	7 to 21 days prior to V1	0	7 (+ 3)	35 (± 7)	63 (+ 6)	77 (+ 7)
Patient Global Impression of Change[e]					x	x	

AUDIT = Alcohol Use Disorders Identification Test; ECG = electrocardiogram; SCL-90 = Symptom Checklist -90R; SF = Short Form; V = Visit

[a] May occur by phone or at a clinic visit, and may be combined with the Screening and Baseline Visit (Visit 0).

[b] If Visit 1 cannot occur within 3 weeks of Visit 0 and the participant is to remain in the study, then Visit 0 will be repeated as an unscheduled visit. The timing of Visits 3-6 and their windows are established from the date of Visit 1.

[c] Includes consent for study participation, consent to store biological specimens for future studies, and Health Insurance Portability and Accountability Act statement, if applicable.

[d] The first dose of study drug should be taken in the evening of the Day 1.

[e] Questionnaires will be distributed at Visit 0 and the participant will return completed questionnaires at Visit 1.

APPENDIX B: PACKAGE INSERT FOR PROPRANOLOL HYDROCHLORIDE EXTENDED-RELEASE

Propranolol hydrochloride extended-release capsules

Rx only

DESCRIPTION

Propranolol hydrochloride is a synthetic beta-adrenergic receptor-blocking agent chemically described as 2-Propanol, 1-[(1-methylethyl)amino]-3-(1-naphthalenyloxy)-, hydrochloride,(±)-. Its molecular and structural formulae are:

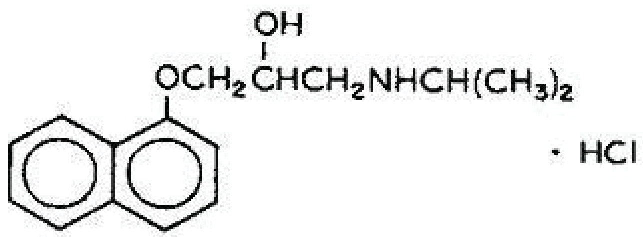

$C_{16}H_{21}NO_2 \cdot HCl$

Propranolol hydrochloride is a stable, white, crystalline solid which is readily soluble in water and ethanol. Its molecular weight is 295.80.

Propranolol hydrochloride extended-release capsules are formulated to provide a sustained release of propranolol hydrochloride. Propranolol hydrochloride extended-release capsules are available as 60 mg, 80 mg, 120 mg, and 160 mg capsules for oral administration.

The inactive ingredients contained in propranolol hydrochloride extended-release capsules are: cellulose, ethylcellulose, gelatin capsules, hypromellose, and titanium dioxide. In addition, propranolol hydrochloride extended-release capsules 60 mg, 80 mg, and 120 mg capsules contain D&C Red No. 28 and FD&C Blue No. 1; propranolol hydrochloride extended-release capsules 160 mg capsules contain FD&C Blue No. 1.

These capsules comply with USP Dissolution Test 1.

CLINICAL PHARMACOLOGY

General

Propranolol is a nonselective, beta-adrenergic receptor-blocking agent possessing no other autonomic nervous system activity. It specifically competes with beta-adrenergic receptor-stimulating agents for available receptor sites. When access to beta-receptor sites is blocked by propranolol, the chronotropic, inotropic, and vasodilator responses to beta-adrenergic stimulation are decreased proportionately. At dosages greater than required for beta blockade, propranolol also exerts a quinidine-like or anesthetic-like membrane action, which affects the cardiac action potential. The significance of the membrane action in the treatment of arrhythmias is uncertain.

Propranolol hydrochloride extended-release capsules should not be considered a simple mg-for-mg substitute for conventional propranolol and the blood levels achieved do not match (are lower than) those of two to four times daily dosing with the same dose (see **DOSAGE AND ADMINISTRATION**). When changing to propranolol hydrochloride extended-release capsules from conventional propranolol, a possible need for retitration upwards should be considered, especially to maintain effectiveness at the end of the dosing interval. In most clinical settings, however, such as hypertension or angina where there is little correlation between plasma levels and clinical effect, propranolol hydrochloride extended-release capsules have been therapeutically equivalent to the same mg dose of conventional propranolol hydrochloride extended-release capsules as assessed by 24-hour effects on blood pressure and on 24-hour exercise responses of heart rate, systolic pressure, and rate pressure product.

Mechanism of Action

The mechanism of the antihypertensive effect of propranolol has not been established. Among the factors that may be involved in contributing to the antihypertensive action include: (1) decreased cardiac output, (2) inhibition of renin release by the kidneys, and (3) diminution of tonic sympathetic nerve outflow from vasomotor centers in the brain. Although total peripheral resistance may increase initially, it readjusts to or below the pretreatment level with chronic use of propranolol. Effects of propranolol on plasma volume appear to be minor and somewhat variable.

In angina pectoris, propranolol generally reduces the oxygen requirement of the heart at any given level of effort by blocking the catecholamine-induced increases in the heart rate, systolic blood pressure, and the velocity and extent of myocardial contraction. Propranolol may increase oxygen requirements by increasing left ventricular fiber length, end diastolic pressure, and systolic ejection period. The net physiologic effect of beta-adrenergic blockade is usually advantageous and is manifested during exercise by delayed onset of pain and increased work capacity.

Propranolol exerts its antiarrhythmic effects in concentrations associated with beta-adrenergic blockade, and this appears to be its principal antiarrhythmic mechanism of action. In dosages greater than required for beta blockade, propranolol also exerts a quinidine-like or anesthetic-like membrane action which affects the cardiac action potential. The significance of the membrane action in the treatment of arrhythmias is uncertain.

The mechanism of the anti-migraine effect of propranolol has not been established. Beta-adrenergic receptors have been demonstrated in the pial vessels of the brain.

PHARMACOKINETICS AND DRUG METABOLISM

Absorption

Propranolol is highly lipophilic and almost completely absorbed after oral administration. However, it undergoes high first pass metabolism by the liver and on average, only about 25% of

propranolol reaches the systemic circulation. Propranolol hydrochloride extended-release capsules (60, 80, 120, and 160 mg) release propranolol HCl at a controlled and predictable rate. Peak blood levels following dosing with propranolol hydrochloride extended-release capsules occur at about 6 hours.

The effect of food on propranolol hydrochloride extended-release capsules bioavailability has not been investigated.

Distribution
Approximately 90% of circulating propranolol is bound to plasma proteins (albumin and alpha-1-acid glycoprotein). The binding is enantiomer-selective. The S(–)-enantiomer is preferentially bound to alpha-1-glycoprotein and the R(+)-enantiomer preferentially bound to albumin. The volume of distribution of propranolol is approximately 4 liters/kg.

Propranolol crosses the blood-brain barrier and the placenta, and is distributed into breast milk.

Metabolism and Elimination
Propranolol is extensively metabolized with most metabolites appearing in the urine. Propranolol is metabolized through three primary routes: aromatic hydroxylation (mainly 4-hydroxylation), N-dealkylation followed by further side-chain oxidation, and direct glucuronidation. It has been estimated that the percentage contributions of these routes to total metabolism are 42%, 41% and 17%, respectively, but with considerable variability between individuals. The four major metabolites are propranolol glucuronide, naphthyloxylactic acid and glucuronic acid, and sulfate conjugates of 4-hydroxy propranolol.

In-vitro studies have indicated that the aromatic hydroxylation of propranolol is catalyzed mainly by polymorphic CYP2D6. Side-chain oxidation is mediated mainly by CYP1A2 and to some extent by CYP2D6. 4-hydroxy propranolol is a weak inhibitor of CYP2D6.

Propranolol is also a substrate of CYP2C19 and a substrate for the intestinal efflux transporter, p-glycoprotein (p-gp). Studies suggest however that p-gp is not dose-limiting for intestinal absorption of propranolol in the usual therapeutic dose range.

In healthy subjects, no difference was observed between CYP2D6 extensive metabolizers (EMs) and poor metabolizers (PMs) with respect to oral clearance or elimination half-life. Partial clearance of 4-hydroxy propranolol was significantly higher and naphthyloxyactic acid was significantly lower in EMs than PMs.

When measured at steady state over a 24-hour period the areas under the propranolol plasma concentration-time curve (AUCs) for the propranolol hydrochloride extended-release capsules are approximately 60% to 65% of the AUCs for a comparable divided daily dose of propranolol hydrochloride extended-release capsules. The lower AUCs for the propranolol hydrochloride

extended-release capsules are due to greater hepatic metabolism of propranolol, resulting from the slower rate of absorption of propranolol. Over a twenty-four (24) hour period, blood levels are fairly constant for about twelve (12) hours, then decline exponentially. The apparent plasma half-life is about 10 hours.

Enantiomers

Propranolol is a racemic mixture of two enantiomers, R(+) and S(–). The S(–)-enantiomer is approximately 100 times as potent as the R(+)-enantiomer in blocking beta adrenergic receptors. In normal subjects receiving oral doses of racemic propranolol, S(–)-enantiomer concentrations exceeded those of the R(+)-enantiomer by 40-90% as a result of stereoselective hepatic metabolism. Clearance of the pharmacologically active S(–)-propranolol is lower than R(+)-propranolol after intravenous and oral doses.

Special Population

Geriatric

The pharmacokinetics of propranolol hydrochloride extended-release capsules have not been investigated in patients over 65 years of age.

In a study of 12 elderly (62-79 years old) and 12 young (25-33 years old) healthy subjects, the clearance of S-enantiomer of propranolol was decreased in the elderly. Additionally, the half-life of both the R- and S-propranolol were prolonged in the elderly compared with the young (11 hours vs. 5 hours).

Clearance of propranolol is reduced with aging due to decline in oxidation capacity (ring oxidation and side chain oxidation). Conjugation capacity remains unchanged. In a study of 32 patients age 30 to 84 years given a single 20-mg dose of propranolol, an inverse correlation was found between age and the partial metabolic clearances to 4-hydroxypropranolol (40HP ring oxidation) and to naphthoxylactic acid (NLA-side chain oxidation). No correlation was found between age and the partial metabolic clearance to propranolol glucuronide (PPLG conjugation).

Gender

In a study of 9 healthy women and 12 healthy men, neither the administration of testosterone nor the regular course of the menstrual cycle affected the plasma binding of the propranolol enantiomers. In contrast, there was a significant, although non-enantioselective diminution of the binding of propranolol after treatment with ethinyl estradiol. These findings are inconsistent with another study, in which administration of testosterone cypionate confirmed the stimulatory role of this hormone on propranolol metabolism and concluded that the clearance of propranolol in men is dependent on circulating concentrations of testosterone. In women, none of the metabolic clearances for propranolol showed any significant association with either estradiol or testosterone.

Race
A study conducted in 12 Caucasian and 13 African-American male subjects taking propranolol, showed that at steady state, the clearance of R(+)- and S(–)-propranolol were about 76% and 53% higher in African-Americans than in Caucasians, respectively.

Chinese subjects had a greater proportion (18% to 45% higher) of unbound propranolol in plasma compared to Caucasians, which was associated with a lower plasma concentration of alpha-1-acid glycoprotein.

Renal Insufficiency
The pharmacokinetics of propranolol hydrochloride extended-release capsules have not been investigated in patients with renal insufficiency.

In a study conducted in 5 patients with chronic renal failure, 6 patients on regular dialysis, and 5 healthy subjects, who received a single oral dose of 40 mg of propranolol, the peak plasma concentrations (C_{max}) of propranolol in the chronic renal failure group were 2 to 3-fold higher (161±41 ng/mL) than those observed in the dialysis patients (47±9 ng/mL) and in the healthy subjects (26±1 ng/mL). Propranolol plasma clearance was also reduced in the patients with chronic renal failure. Studies have reported a delayed absorption rate and a reduced half-life of propranolol in patients with renal failure of varying severity. Despite this shorter plasma half-life, propranolol peak plasma levels were 3-4 times higher and total plasma levels of metabolites were up to 3 times higher in these patients than in subjects with normal renal function.

Chronic renal failure has been associated with a decrease in drug metabolism via down regulation of hepatic cytochrome P450 activity resulting in a lower "first-pass" clearance. Propranolol is not significantly dialyzable. Hepatic Insufficiency
The pharmacokinetics of propranolol hydrochloride extended-release capsules have not been investigated in patients with hepatic insufficiency.

Propranolol is extensively metabolized by the liver. In a study conducted in 6 patients with cirrhosis and 7 healthy subjects receiving 160 mg of a long-acting preparation of propranolol once a day for 7 days, the steady-state propranolol concentration in patients with cirrhosis was increased 2.5-fold in comparison to controls. In the patients with cirrhosis, the half-life obtained after a single intravenous dose of 10 mg propranolol increased to 7.2 hours compared to 2.9 hours in control (see **PRECAUTIONS**).

Drug Interactions
All drug interaction studies were conducted with propranolol. There are no data on drug interactions with propranolol hydrochloride extended-release capsules capsules.

<u>Interactions with Substrates, Inhibitors or Inducers of Cytochrome P-450 Enzymes</u>
Because propranolol's metabolism involves multiple pathways in the Cytochrome P-450 system (CYP2D6, 1A2, 2C19), co-administration with drugs that are metabolized by, or affect the

activity (induction or inhibition) of one or more of these pathways may lead to clinically relevant drug interactions (see **Drug Interactions** under **PRECAUTIONS**).

Substrates or Inhibitors of CYP2D6
Blood levels and/or toxicity of propranolol may be increased by co-administration with substrates or inhibitors of CYP2D6, such as amiodarone, cimetidine, delavudin, fluoxetine, paroxetine, quinidine, and ritonavir. No interactions were observed with either ranitidine or lansoprazole.

Substrates or Inhibitors of CYP1A2
Blood levels and/or toxicity of propranolol may be increased by co-administration with substrates or inhibitors of CYP1A2, such as imipramine, cimetidine, ciprofloxacin, fluvoxamine, isoniazid, ritonavir, theophylline, zileuton, zolmitriptan, and rizatriptan.

Substrates or Inhibitors of CYP2C19
Blood levels and/or toxicity of propranolol may be increased by co-administration with substrates or inhibitors of CYP2C19, such as fluconazole, cimetidine, fluoxetine, fluvoxamine, tenioposide, and tolbutamide. No interaction was observed with omeprazole.

Inducers of Hepatic Drug Metabolism
Blood levels of propranolol may be decreased by co-administration with inducers such as rifampin, ethanol, phenytoin, and phenobarbital. Cigarette smoking also induces hepatic metabolism and has been shown to increase up to 77% the clearance of propranolol, resulting in decreased plasma concentrations.

Cardiovascular Drugs
Antiarrhythmics
The AUC of propafenone is increased by more than 200% by co-administration of propranolol.

The metabolism of propranolol is reduced by co-administration of quinidine, leading to a two to three fold increased blood concentration and greater degrees of clinical beta-blockade.

The metabolism of lidocaine is inhibited by co-administration of propranolol, resulting in a 25% increase in lidocaine concentrations.

Calcium Channel Blockers
The mean C_{max} and AUC of propranolol are increased respectively, by 50% and 30% by co-administration of nisoldipine and by 80% and 47%, by co-administration of nicardipine.

The mean C_{max} and AUC of nifedipine are increased by 64% and 79%, respectively, by co-administration of propranolol.

Propranolol does not affect the pharmacokinetics of verapamil and norverapamil. Verapamil does not affect the pharmacokinetics of propranolol.

Non-Cardiovascular Drugs

Migraine Drugs

Administration of zolmitriptan or rizatriptan with propranolol resulted in increased concentrations of zolmitriptan (AUC increased by 56% and C_{max} by 37%) or rizatriptan (the AUC and C_{max} were increased by 67% and 75%, respectively).

Theophylline

Co-administration of theophylline with propranolol decreases theophylline oral clearance by 30% to 52%.

Benzodiazepines

Propranolol can inhibit the metabolism of diazepam, resulting in increased concentrations of diazepam and its metabolites. Diazepam does not alter the pharmacokinetics of propranolol.

The pharmacokinetics of oxazepam, triazolam, lorazepam, and alprazolam are not affected by co-administration of propranolol.

Neuroleptic Drugs

Co-administration of long-acting propranolol at doses greater than or equal to 160 mg/day resulted in increased thioridazine plasma concentrations ranging from 55% to 369% and increased thioridazine metabolite (mesoridazine) concentrations ranging from 33% to 209%.

Co-administration of chlorpromazine with propranolol resulted in a 70% increase in propranolol plasma level.

Anti-Ulcer Drugs

Co-administration of propranolol with cimetidine, a non-specific CYP450 inhibitor, increased propranolol AUC and C_{max} by 46% and 35%, respectively. Co-administration with aluminum hydroxide gel (1200 mg) may result in a decrease in propranolol concentrations.

Co-administration of metoclopramide with the long-acting propranolol did not have a significant effect on propranolol’s pharmacokinetics.

Lipid Lowering Drugs

Co-administration of cholestyramine or colestipol with propranolol resulted in up to 50% decrease in propranolol concentrations.

Co-administration of propranolol with lovastatin or pravastatin, decreased 18% to 23% the AUC of both, but did not alter their pharmacodynamics. Propranolol did not have an effect on the pharmacokinetics of fluvastatin.

Warfarin

Concomitant administration of propranolol and warfarin has been shown to increase warfarin bioavailability and increase prothrombin time.

PHARMACODYNAMICS AND CLINICAL EFFECTS

Hypertension

In a retrospective, uncontrolled study, 107 patients with diastolic blood pressure 110 to 150 mmHg received propranolol 120 mg t.i.d. for at least 6 months, in addition to diuretics and potassium, but with no other hypertensive agent. Propranolol contributed to control of diastolic blood pressure, but the magnitude of the effect of propranolol on blood pressure cannot be ascertained.

Four double-blind, randomized, crossover studies were conducted in a total of 74 patients with mild or moderately severe hypertension treated with propranolol hydrochloride extended-release capsules 160 mg once daily or propranolol 160 mg given either once daily or in two 80 mg doses. Three of these studies were conducted over a 4-week treatment period. One study was assessed after a 24-hour period. Propranolol hydrochloride extended-release capsules were as effective as propranolol in controlling hypertension (pulse rate, systolic and diastolic blood pressure) in each of these trials.

Angina Pectoris

In a double-blind, placebo-controlled study of 32 patients of both sexes, aged 32 to 69 years, with stable angina, propranolol 100 mg t.i.d. was administered for 4 weeks and shown to be more effective than placebo in reducing the rate of angina episodes and in prolonging total exercise time.

Twelve male patients with moderately severe angina pectoris were studied in a double-blind, crossover study. Patients were randomized to either Propranolol hydrochloride extended-release capsules 160 mg daily or conventional propranolol 40 mg four times a day for 2 weeks.

Nitroglycerine tablets were allowed during the study. Blood pressure, heart rate and ECG's were recorded during serial exercise treadmill testing. Propranolol hydrochloride extended-release capsules were as effective as conventional propranolol for exercise heart rate, systolic and diastolic blood pressure, duration of anginal pain and ST-segment depression before or after exercise, exercise duration, angina attack rate and nitroglycerine consumption.

In another double-blind, randomized, crossover trial, the effectiveness of propranolol hydrochloride extended-release capsules 160 mg daily and conventional propranolol 40 mg four times a day were evaluated in 13 patients with angina. ECG's were recorded while patients exercised until angina developed. Propranolol hydrochloride extended-release capsules were as effective as conventional propranolol for amount of exercise performed, ST-segment depression, number of anginal attacks, amount of nitroglycerine consumed, systolic and diastolic blood pressures and heart rate at rest and after exercise.

Migraine

In a 34-week, placebo-controlled, 4-period, dose-finding crossover study with a double-blind randomized treatment sequence, 62 patients with migraine received propranolol 20 to 80 mg 3 or 4 times daily. The headache unit index, a composite of the number of days with headache and the associated severity of the headache, was significantly reduced for patients receiving propranolol as compared to those on placebo.

Hypertrophic Subaortic Stenosis

In an uncontrolled series of 13 patients with New York Heart Association (NYHA) class 2 or 3 symptoms and hypertrophic subaortic stenosis diagnosed at cardiac catheterization, oral propranolol 40-80 mg t.i.d. was administered and patients were followed for up to 17 months. Propranolol was associated with improved NYHA class for most patients.

INDICATIONS AND USAGE

Hypertension

Propranolol hydrochloride extended-release capsules are indicated in the management of hypertension. It may be used alone or used in combination with other antihypertensive agents, particularly a thiazide diuretic. Propranolol hydrochloride extended-release capsules is not indicated in the management of hypertensive emergencies.

Angina Pectoris Due to Coronary Atherosclerosis

Propranolol hydrochloride extended-release capsules are indicated to decrease angina frequency and increase exercise tolerance in patients with angina pectoris.

Migraine

Propranolol hydrochloride extended-release capsules are indicated for the prophylaxis of common migraine headache. The efficacy of propranolol in the treatment of a migraine attack that has started has not been established, and propranolol is not indicated for such use.

Hypertrophic Subaortic Stenosis

Propranolol hydrochloride extended-release capsules improve NYHA functional class in symptomatic patients with hypertrophic subaortic stenosis.

CONTRAINDICATIONS

Propranolol is contraindicated in 1) cardiogenic shock; 2) sinus bradycardia and greater than first-degree block; 3) bronchial asthma; and 4) in patients with known hypersensitivity to propranolol hydrochloride.

WARNINGS

Angina Pectoris

There have been reports of exacerbation of angina and, in some cases, myocardial infarction, following abrupt discontinuance of propranolol therapy. Therefore, when discontinuance of propranolol is planned, the dosage should be gradually reduced over at least a few weeks, and the patient should be cautioned against interruption or cessation of therapy without the physician's

advice. If propranolol therapy is interrupted and exacerbation of angina occurs, it usually is advisable to reinstitute propranolol therapy and take other measures appropriate for the management of unstable angina pectoris. Since coronary artery disease may be unrecognized, it may be prudent to follow the above advice in patients considered at risk of having occult atherosclerotic heart disease who are given propranolol for other indications.

Hypersensitivity and Skin Reactions
Hypersensitivity reactions, including anaphylactic/anaphylactoid reactions, have been associated with the administration of propranolol (see **ADVERSE REACTIONS**).

Cutaneous reactions, including Stevens-Johnson Syndrome, toxic epidermal necrolysis, exfoliative dermatitis, erythema multiforme, and urticaria, have been reported with use of propranolol (see **ADVERSE REACTIONS**).

Cardiac Failure
Sympathetic stimulation may be a vital component supporting circulatory function in patients with congestive heart failure, and its inhibition by beta blockade may precipitate more severe failure. Although beta blockers should be avoided in overt congestive heart failure, some have been shown to be highly beneficial when used with close follow-up in patients with a history of failure who are well compensated and are receiving diuretics as needed. Beta-adrenergic blocking agents do not abolish the inotropic action of digitalis on heart muscle.

In Patients without a History of Heart Failure, continued use of beta blockers can, in some cases, lead to cardiac failure.

Nonallergic Bronchospasm (e.g., Chronic Bronchitis, Emphysema)
In general, patients with bronchospastic lung disease should not receive beta-blockers. Propranolol should be administered with caution in this setting since it may provoke a bronchial asthmatic attack by blocking bronchodilation produced by endogenous and exogenous catecholamine stimulation of beta-receptors.

Major Surgery
Chronically administered beta-blocking therapy should not be routinely withdrawn prior to major surgery, however the impaired ability of the heart to respond to reflex adrenergic stimuli may augment the risks of general anesthesia and surgical procedures.

Diabetes and Hypoglycemia
Beta-adrenergic blockade may prevent the appearance of certain premonitory signs and symptoms (pulse rate and pressure changes) of acute hypoglycemia, especially in labile insulin-dependent diabetics. In these patients, it may be more difficult to adjust the dosage of insulin.

Propranolol therapy, particularly when given to infants and children, diabetic or not, has been associated with hypoglycemia especially during fasting as in preparation for surgery.

Hypoglycemia has been reported in patients taking propranolol after prolonged physical exertion and in patients with renal insufficiency.

Thyrotoxicosis
Beta-adrenergic blockade may mask certain clinical signs of hyperthyroidism. Therefore, abrupt withdrawal of propranolol may be followed by an exacerbation of symptoms of hyperthyroidism, including thyroid storm. Propranolol may change thyroid-function tests, increasing T4 and reverse T3, and decreasing T3.

Wolff-Parkinson-White Syndrome
Beta-adrenergic blockade in patients with Wolff-Parkinson-White syndrome and tachycardia has been associated with severe bradycardia requiring treatment with a pacemaker. In one case, this result was reported after an initial dose of 5 mg propranolol.

PRECAUTIONS
General
Propranolol should be used with caution in patients with impaired hepatic or renal function. Propranolol hydrochloride extended-release capsules are not indicated for the treatment of hypertensive emergencies.

Beta-adrenergic receptor blockade can cause reduction of intraocular pressure. Patients should be told that propranolol hydrochloride extended-release capsules may interfere with the glaucoma screening test. Withdrawal may lead to a return of increased intraocular pressure.

While taking beta-blockers, patients with a history of severe anaphylactic reaction to a variety of allergens may be more reactive to repeated challenge, either accidental, diagnostic, or therapeutic. Such patients may be unresponsive to the usual doses of epinephrine used to treat allergic reaction.

Clinical Laboratory Tests
In patients with hypertension, use of propranolol has been associated with elevated levels of serum potassium, serum transaminases, and alkaline phosphatase. In severe heart failure, the use of propranolol has been associated with increases in Blood Urea Nitrogen.

Drug Interactions
Caution should be exercised when propranolol hydrochloride extended-release capsules are administered with drugs that have an affect on CYP2D6, 1A2, or 2C19 metabolic pathways. Co-administration of such drugs with propranolol may lead to clinically relevant drug interactions and changes on its efficacy and/or toxicity (see **Drug Interactions** in **PHARMACOKINETICS AND DRUG METABOLISM**). Alcohol when used concomitantly with propranolol, may increase plasma levels of propranolol.

Cardiovascular Drugs

Antiarrhythmics

Propafenone has negative inotropic and beta-blocking properties that can be additive to those of propranolol.

Quinidine increases the concentration of propranolol and produces greater degrees of clinical beta-blockade and may cause postural hypotension.

Amiodarone is an antiarrhythmic agent with negative chronotropic properties that may be additive to those seen with β-blockers such as propranolol.

The clearance of lidocaine is reduced with administration of propranolol. Lidocaine toxicity has been reported following co-administration with propranolol.

Caution should be exercised when administering propranolol hydrochloride extended-release capsules with drugs that slow A-V nodal conduction, e.g., lidocaine and calcium channel blockers.

Digitalis Glycosides

Both digitalis glycosides and beta-blockers slow atrioventricular conduction and decrease heart rate. Concomitant use can increase the risk of bradycardia.

Calcium Channel Blockers

Caution should be exercised when patients receiving a beta-blocker are administered a calcium-channel-blocking drug with negative inotropic and/or chronotropic effects. Both agents may depress myocardial contractility or atrioventricular conduction.

There have been reports of significant bradycardia, heart failure, and cardiovascular collapse with concurrent use of verapamil and beta-blockers.

Co-administration of propranolol and diltiazem in patients with cardiac disease has been associated with bradycardia, hypotension, high degree heart block, and heart failure.

ACE Inhibitors

When combined with beta-blockers, ACE inhibitors can cause hypotension, particularly in the setting of acute myocardial infarction.

The antihypertensive effects of clonidine may be antagonized by beta-blockers. Propranolol hydrochloride extended-release capsules should be administered cautiously to patients withdrawing from clonidine.

Alpha Blockers

Prazosin has been associated with prolongation of first dose hypotension in the presence of beta-blockers.

Postural hypotension has been reported in patients taking both beta-blockers and terazosin or doxazosin.

Reserpine

Patients receiving catecholamine-depleting drugs, such as reserpine should be closely observed for excessive reduction of resting sympathetic nervous activity, which may result in hypotension, marked bradycardia, vertigo, syncopal attacks, or orthostatic hypotension.

Inotropic Agents

Patients on long-term therapy with propranolol may experience uncontrolled hypertension if administered epinephrine as a consequence of unopposed alpha-receptor stimulation. Epinephrine is therefore not indicated in the treatment of propranolol overdose (see **OVERDOSAGE**).

Isoproterenol and Dobutamine

Propranolol is a competitive inhibitor of beta-receptor agonists, and its effects can be reversed by administration of such agents, e.g., dobutamine or isoproterenol. Also, propranolol may reduce sensitivity to dobutamine stress echocardiography in patients undergoing evaluation for myocardial ischemia.

Non-Cardiovascular Drugs

Nonsteroidal Anti-Inflammatory Drugs

Nonsteroidal anti-inflammatory drugs (NSAIDs) have been reported to blunt the antihypertensive effect of beta-adrenoreceptor blocking agents.

Administration of indomethacin with propranolol may reduce the efficacy of propranolol in reducing blood pressure and heart rate.

Antidepressants

The hypotensive effects of MAO inhibitors or tricyclic antidepressants may be exacerbated when administered with beta-blockers by interfering with the beta blocking activity of propranolol.

Anesthetic Agents

Methoxyflurane and trichloroethylene may depress myocardial contractility when administered with propranolol.

Warfarin

Propranolol when administered with warfarin increases the concentration of warfarin. Prothrombin time, therefore, should be monitored.

Neuroleptic Drugs

Hypotension and cardiac arrest have been reported with the concomitant use of propranolol and haloperidol.

Thyroxine
Thyroxine may result in a lower than expected T3 concentration when used concomitantly with propranolol.

Carcinogenesis, Mutagenesis, Impairment of Fertility
In dietary administration studies in which mice and rats were treated with propranolol hydrochloride for up to 18 months at doses of up to 150 mg/kg/day, there was no evidence of drug-related tumorigenesis. On a body surface area basis, this dose in the mouse and rat is, respectively, about equal to and about twice the maximum recommended human oral daily dose (MRHD) of 640 mg propranolol hydrochloride. In a study in which both male and female rats were exposed to propranolol hydrochloride in their diets at concentrations of up to 0.05% (about 50 mg/kg body weight and less than the MRHD), from 60 days prior to mating and throughout pregnancy and lactation for two generations, there were no effects on fertility. Based on differing results from Ames Tests performed by different laboratories, there is equivocal evidence for a genotoxic effect of propranolol in bacteria (*S. typhimurium* strain TA 1538).

Pregnancy: Pregnancy Category C
In a series of reproductive and developmental toxicology studies, propranolol was given to rats by gavage or in the diet throughout pregnancy and lactation. At doses of 150 mg/kg/day, but not at doses of 80 mg/kg/day (equivalent to the MRHD on a body surface area basis), treatment was associated with embryotoxicity (reduced litter size and increased resorption rates) as well as neonatal toxicity (deaths). Propranolol hydrochloride also was administered (in the feed) to rabbits (throughout pregnancy and lactation) at doses as high as 150 mg/kg/day (about 5 times the maximum recommended human oral daily dose). No evidence of embryo or neonatal toxicity was noted.

There are no adequate and well-controlled studies in pregnant women. Intrauterine growth retardation, small placentas, and congenital abnormalities have been reported in neonates whose mothers received propranolol during pregnancy. Neonates whose mothers are receiving propranolol at parturition have exhibited bradycardia, hypoglycemia and/or respiratory depression. Adequate facilities for monitoring such infants at birth should be available. Propranolol hydrochloride extended-release capsules should be used during pregnancy only if the potential benefit justifies the potential risk to the fetus.

Nursing Mothers
Propranolol is excreted in human milk. Caution should be exercised when propranolol hydrochloride extended-release capsules are administered to a nursing woman.

Pediatric Use
Safety and effectiveness of propranolol in pediatric patients have not been established.

Bronchospasm and congestive heart failure have been reported coincident with the administration of propranolol therapy in pediatric patients.

Geriatric Use

Clinical studies of propranolol hydrochloride extended-release capsules did not include sufficient numbers of subjects aged 65 and over to determine whether they respond differently from younger subjects. Other reported clinical experience has not identified differences in responses between the elderly and younger patients. In general, dose selection for an elderly patient should be cautious, usually starting at the low end of the dosing range, reflecting the greater frequency of the decreased hepatic, renal or cardiac function, and of concomitant disease or other drug therapy.

ADVERSE REACTIONS

The following adverse events were observed and have been reported in patients using propranolol.

Cardiovascular: Bradycardia; congestive heart failure; intensification of AV block; hypotension; paresthesia of hands; thrombocytopenic purpura; arterial insufficiency, usually of the Raynaud type.

Central Nervous System: Light-headedness; mental depression manifested by insomnia, lassitude, weakness, fatigue; catatonia; visual disturbances; hallucinations; vivid dreams; an acute reversible syndrome characterized by disorientation for time and place, short-term memory loss, emotional lability, slightly clouded sensorium, and decreased performance on neuropsychometrics. For immediate release formulations, fatigue, lethargy, and vivid dreams appear dose related.

Gastrointestinal: Nausea, vomiting, epigastric distress, abdominal cramping, diarrhea, constipation, mesenteric arterial thrombosis, ischemic colitis.

Allergic: Hypersensitivity reactions, including anaphylactic/anaphylactoid reactions; pharyngitis and agranulocytosis; erythematous rash; fever combined with aching and sore throat; laryngospasm; respiratory distress.

Respiratory: Bronchospasm.

Hematologic: Agranulocytosis, nonthrombocytopenic purpura, and thrombocytopenic purpura.

Autoimmune: Systemic lupus erythematosus (SLE).

Skin and mucous membranes: Stevens-Johnson Syndrome, toxic epidermal necrolysis, dry eyes, exfoliative dermatitis, erythema multiforme, urticaria, alopecia, SLE-like reactions, and psoriasisiform rashes. Oculomucocutaneous syndrome involving the skin, serous membranes,

and conjunctivae reported for a beta-blocker (practolol) have not been associated with propranolol.

Genitourinary: Male impotence; Peyronie's disease.

OVERDOSAGE

Propranolol is not significantly dialyzable. In the event of overdosage or exaggerated response, the following measures should be employed:

General: If ingestion is or may have been recent, evacuate gastric contents, taking care to prevent pulmonary aspiration.

Supportive Therapy: Hypotension and bradycardia have been reported following propranolol overdose and should be treated appropriately. Glucagon can exert potent inotropic and chronotropic effects and may be particularly useful for the treatment of hypotension or depressed myocardial function after a propranolol overdose. Glucagon should be administered as 50-150 mcg/kg intravenously followed by continuous drip of 1-5 mg/hour for positive chronotropic effect. Isoproterenol, dopamine or phosphodiesterase inhibitors may also be useful. Epinephrine, however, may provoke uncontrolled hypertension. Bradycardia can be treated with atropine or isoproterenol. Serious bradycardia may require temporary cardiac pacing.

The electrocardiogram, pulse, blood pressure, neurobehavioral status and intake and output balance must be monitored. Isoproterenol and aminophylline may be used for bronchospasm.

DOSAGE AND ADMINISTRATION

General

Propranolol hydrochloride extended-release capsules provide propranolol hydrochloride in a sustained-release capsule for administration once daily. If patients are switched from propranolol hydrochloride tablets to propranolol hydrochloride extended-release capsules, care should be taken to assure that the desired therapeutic effect is maintained. Propranolol hydrochloride extended-release capsules should not be considered a simple mg-for-mg substitute for propranolol hydrochloride tablets. Propranolol hydrochloride extended-release capsules have different kinetics and produces lower blood levels. Retitration may be necessary, especially to maintain effectiveness at the end of the 24-hour dosing interval.

Hypertension

The usual initial dosage is 80 mg propranolol hydrochloride extended-release capsules once daily, whether used alone or added to a diuretic. The dosage may be increased to 120 mg once daily or higher until adequate blood pressure control is achieved. The usual maintenance dosage is 120 to 160 mg once daily. In some instances a dosage of 640 mg may be required. The time needed for full hypertensive response to a given dosage is variable and may range from a few days to several weeks.

Angina Pectoris
Starting with 80 mg propranolol hydrochloride extended-release capsules once daily, dosage should be gradually increased at three- to seven-day intervals until optimal response is obtained. Although individual patients may respond at any dosage level, the average optimal dosage appears to be 160 mg once daily. In angina pectoris, the value and safety of dosage exceeding 320 mg per day have not been established.

If treatment is to be discontinued, reduce dosage gradually over a period of a few weeks (see **“WARNINGS”**).

Migraine
The initial oral dose is 80 mg propranolol hydrochloride extended-release capsules once daily. The usual effective dose range is 160 to 240 mg once daily. The dosage may be increased gradually to achieve optimal migraine prophylaxis. If a satisfactory response is not obtained within four to six weeks after reaching the maximal dose, propranolol hydrochloride extended-release capsules therapy should be discontinued. It may be advisable to withdraw the drug gradually over a period of several weeks depending on the patient's age, comorbidity, and dose of propranolol hydrochloride extended-release capsules.

Hypertrophic Subaortic Stenosis
The usual dosage is 80 to 160 mg propranolol hydrochloride extended-release capsules once daily.

HOW SUPPLIED
Propranolol hydrochloride extended-release capsules

Each white capsule, identified by 3 narrow bands, 1 wide band, and “AK 60,” contains 60 mg of propranolol hydrochloride in bottles of 100 (NDC 43478-900-88).

Each white/light blue capsule, identified by 3 narrow bands, 1 wide band, and “AK 80,” contains 80 mg of propranolol hydrochloride in bottles of 100 (NDC 43478-901-88).

Each white/dark blue capsule, identified by 3 narrow bands, 1 wide band, and “AK 120,” contains 120 mg of propranolol hydrochloride in bottles of 100 (NDC 43478-902-88).

Each dark blue/light blue capsule, identified by 3 narrow bands, 1 wide band, and “AK 160,” contains 160 mg of propranolol hydrochloride in bottles of 100 (NDC 43478-903-88).

Store at 20° to 25°C (68° to 77°F); excursions permitted to 15° to 30°C (59° to 86°F). [See USP Controlled Room Temperature]

Protect from light, moisture, freezing, and excessive heat.

Dispense in a tight, light-resistant container as defined in the USP.

This product's label may have been updated. For current package insert and further product information, please call our medical communications department toll-free at 1-877-567-0862

Manufactured for Rouses Point Pharmaceuticals, LLC
Cranford, NJ 07016
By Wyeth Pharmaceuticals, Inc.
Philadelphia, PA 19101

490F001
Rev 12/10

Statistical Analysis Plan

Protocol Title: Effect of *COMT* Genetic Polymorphisms on Response to Propranolol Therapy in Temporomandibular Disorder

Protocol Number: 14-067-E

**Version Number and Date:**	Version 3.0; 21 February 2018
**Funding Sponsor:**	National Institute of Dental and Craniofacial Research
**Study Principal Investigator:**	Inna Tchivileva, MD
**SAP Prepared by:**	Robert James / Gary Slade BDSc, Dip DPH, PhD
**Analyses to be Conducted by:**	Robert James / Gary Slade BDSc, Dip DPH, PhD

This document is confidential and proprietary to the sponsor. Acceptance of this document constitutes agreement by the recipient that no unpublished information contained herein will be reproduced, published, or otherwise disclosed without the prior written approval of the sponsor, except that this document may be disclosed to appropriate Institutional Review Boards under the condition that they keep the information confidential.

DOCUMENT VERSION CONTROL

Version Number	Date	Comments/Changes
1.0	22 May 2013	Created by the study team prior to external review of the protocol.
2.0	27 June 2017	Added NIDCR protocol number and protocol short title; updated based on protocols V4.0-11.0

Version Number	Date	Comments/Changes
3.0	21 February 2018	Minor changes involving grammar, wordsmithing, punctuation, and other editorial changes have been made throughout the document.<br><br>Added and removed text to reflect updates based on protocol v12.0.

APPROVALS

Approved: Inna Tchivileva Digitally signed by Inna Tchivileva Date: 2018.05.22 10:48:57 -04'00' Date: ______

Inna Tchivileva, MD
Principal Investigator
UNC School of Dentistry

Gary Slade Digitally signed by Gary Slade DN: cn=Gary Slade, o, ou, email=gary_slade@dentistry.unc.edu, c=US Date: 2018.05.22 11:01:35 -04'00' Date: ______

Gary Slade, BDSc, Dip DPH, PhD
Co-Principal Investigator
UNC School of Dentistry

Robert James Digitally signed by Robert James DN: dc=com, dc=rhoworld, dc=ms, ou=Departments, ou=_Users, ou=Blue, cn=Robert James, email=robert_james@rhoworld.com Date: 2018.05.23 15:19:57 -04'00' Date: ______

Robert James, MS
Statistical Scientist
Data Coordinating Center
Rho, Inc

Samuel Arbes Digitally signed by Samuel Arbes DN: dc=com, dc=rhoworld, dc=ms, ou=Departments, ou=_Users, ou=Red, cn=Samuel Arbes, email=Samuel_Arbes@rhoworld.com Date: 2018.05.23 09:39:15 -04'00' Date: ______

Samuel Arbes, DDS, MPH, PhD
Principal Investigator
Data Coordinating Center
Rho, Inc.

TABLE OF CONTENTS

1. PURPOSE OF THE ANALYSES 5
2. PROTOCOL SUMMARY 5
2.1 Study Objectives 5
2.1.1 Primary Objective 5
2.1.2 Secondary Objectives 5
2.1.3 Exploratory Objectives 6
2.2 Overall Study Design and Plan 6
2.3 Study Population 6
2.4 Treatment Regimens 6
2.5 Treatment Group Assignments or Randomization 6
2.6 Sample Size Determination 7
3. GENERAL ANALYSIS AND REPORTING CONVENTIONS 7
4. ANALYSIS POPULATIONS 8
4.1 Intention-to-Treat Population 8
4.2 Per-Protocol Population 9
4.3 Safety Population 9
5. STUDY PATIENTS 9
5.1 Disposition of Patients 9
5.2 Protocol Deviations 9
6. DEMOGRAPHIC AND OTHER BASELINE CHARACTERISTICS 10
7. MEASUREMENTS OF TREATMENT COMPLIANCE 10
8. EFFICACY EVALUATION 11
8.1 Handling of Dropouts or Missing Data 11
8.2 Efficacy Variables 11
8.3 Analysis Methods 14
8.3.1 Primary Efficacy Analyses 14
8.3.2 Secondary Efficacy Analyses 15
8.3.3 Exploratory Efficacy Analyses 17
9. SAFETY EVALUATION 18
9.1 Overview of Safety Analysis Methods 18
9.2 Adverse Events 18
9.3 Deaths, Serious Adverse Events, and Other Significant Adverse Events 19
9.4 Vital Signs and Other Observations Related to Safety 19
9.5 Unanticipated Problems 20
10. INTERIM ANALYSES AND DATA MONITORING 21
11. CHANGES TO THE ANALYSES PLANNED IN THE PROTOCOL 21
12. APPENDICES 22
12.1 Study Schematic 22
12.2 Schedule of Events 23

LIST OF ABBREVIATIONS

AE	Adverse event
COMT	Catechol-O-methyltransferase
DCC	Data Coordinating Center
DSMB	Data and Safety Monitoring Board
ECG	Electrocardiogram
GLMM	Generalized Linear Mixed Model
HIT	Headache Impact Test
ITT	Intention-to-Treat Population
LPS	Low pain sensitive
NIDCR	National Institute of Dental and Craniofacial Research
NIH	National Institutes of Health
PP	Per-Protocol Population
PPT	Pressure pain threshold
SAE	Serious adverse event
SAP	Statistical Analysis Plan
SF-12	Short Form 12 Health Survey
TEAE	Treatment emergent adverse event
TMD	Temporomandibular disorder
TMJ	Temporomandibular joint
Val Met	Valine allele of the *COMT* gene
V1	Study visit 1, the study of the randomization and start of study drug administration
V2	Study visit 2 at 1 week post-randomization
V3	Study visit 3 at 5 week post-randomization
V4	Study visit 4 at 9 week post-randomization

1. PURPOSE OF THE ANALYSES

The purpose of the analyses is to quantify and test associations described in the primary, secondary, and exploratory objectives of the protocol.

2. PROTOCOL SUMMARY

A total of 200 participants aged 18 to 65 years with chronic temporomandibular disorder (TMD), at three clinic sites, will be randomized in a 1:1 ratio to propranolol or placebo in a parallel-group, masked clinical trial. Participants will attend 6 clinic visits over a period of 12 to 15 weeks.

Potential participants will be prescreened by telephone or at a clinic visit. During the Screening and Baseline Visit, participants will be consented, evaluated for eligibility, and assessed for baseline characteristics. Baseline procedures will include clinical tests and examinations and the administration of questionnaires.

At the Randomization and Titration Visit, participants will be randomized to propranolol or placebo and will begin a 10-week drug treatment phase that is divided into 1 week of drug titration, 8 weeks of drug maintenance, and 1 week of drug tapering. During treatment, many of the baseline assessments will be repeated. The final study visit will occur 1 week after drug tapering ends.

The primary endpoint will be a weekly pain index derived from a daily symptom diary. Secondary endpoints will be additional ratings of clinical pain; examiner assessments of experimental pain sensitivity; participant ratings of physical function, emotional function, and global improvement; occurrence of symptoms and adverse events; and use of rescue medications. DNA analyses will be conducted to identify polymorphisms in the gene that encodes catechol-O-methyltransferase (COMT), an enzyme that metabolizes catecholamines such as epinephrine, norepinephrine, and dopamine and is associated with responses to pain.

2.1 Study Objectives

2.1.1 Primary Objective

The primary objective is to investigate the efficacy of extended-release propranolol compared to placebo in the reduction of the pain index in participants with TMD at week 9 of treatment (at Visit 4).

2.1.2 Secondary Objectives

The secondary objectives are:

- To investigate gene-by-treatment group interaction to determine whether the efficacy of extended-release propranolol in the reduction of the pain index varies according to participants' polymorphisms in the coding region of the *COMT* gene.

- To investigate the efficacy of extended-release propranolol compared with placebo using secondary endpoints: additional pain ratings; examiner assessments of experimental pain sensitivity; participant ratings of physical function, emotional function, global impression of change; occurrence of symptoms and AEs; and use of rescue medications.

2.1.3 Exploratory Objectives

The exploratory objectives are:

- To investigate whether the efficacy of extended-release propranolol in the reduction of the pain index varies according to participants' polymorphisms including, but not limited to three other genetic regions: a) the *COMT* promoter area; b) adrenergic receptor β2 *(ADRB2)* gene; and c) the adrenergic receptor β3 *(ADRB3)* gene.

- To investigate whether the efficacy of extended-release propranolol in the reduction of the pain index varies according to various phenotypic characteristics.
- To collect and archive biospecimens for future biological and statistical analyses.

2.2 Overall Study Design and Plan

This investigation is a two-arm, parallel-group, placebo-controlled, masked, Phase II clinical trial. Participants will attend 6 clinic visits over a period of 12 to 15 weeks, and the trial will take approximately 3 years to complete. The study schematic and schedule of events are provided in Sections 12.1 and 12.2.

Throughout this statistical analysis plan (SAP), V1, V2, V3, V4, and V5 refer respectively to study visit 1 (randomization and start of drug treatment), visit 2 (1 week post randomization), visit 3 (5 weeks post randomization), visit 4 (9 weeks post randomization), and visit 5 (11 weeks post randomization and 1 week after the planned termination of study drug administration).

2.3 Study Population

A total of 200 participants aged 18 to 65 years of either sex and any race/ethnicity with chronic temporomandibular disorder (TMD), at three clinic sites, will be enrolled.

2.4 Treatment Regimens

Participants will be randomized to propranolol or placebo and will begin a 10-week drug treatment phase that is divided into 1 week of drug titration, 8 weeks of drug maintenance, and 1 week of drug tapering. The titration dose and the tapering dose are 60 mg once per day by mouth whereas the maintenance dose is 60 mg twice per day by mouth.

2.5 Treatment Group Assignments or Randomization

At the Randomization and Titration Visit, participants will be randomized 1:1 to propranolol or placebo. Randomization will be stratified by site and conducted within blocks to insure equal

numbers of participants per arm. Randomization will be accomplished by a web-based randomization system developed and maintained by the Data Coordinating Center (DCC).

2.6 Sample Size Determination

This study plans to randomize 200 participants, which will provide 94% power (alpha = 0.05) to detect a difference between a 27% reduction in pain intensity in the propranolol arm and a 9% reduction in the placebo arm (primary hypothesis). Assuming haplotype distributions and within-haplotype treatment effects that are similar to those observed in the pilot study, the same sample size would provide 61% power (alpha = 0.05) to detect an interaction between haplotype and treatment group (secondary hypothesis).

Power calculations were made using the GLMPOWER procedure in SAS, which assumes a data structure of one observation per person. This is a simplification of the situation that will occur with the linear mixed model, described in Section 10.4.1, where efficacy will be evaluated using a data structure where most participants will have three observations per person. The simplified power calculations are shown here because conventional software for power calculations does not deal with the more complex situation of linear mixed models.

3. GENERAL ANALYSIS AND REPORTING CONVENTIONS

The following is a list of general analysis and reporting conventions to be applied for this study.

- Categorical variables will be summarized using counts (n) and percentages (%) and will be presented in the form n (%).

- Moment statistics including mean and standard deviation will be reported at 1 more significant digit than the precision of the data.

- Order statistics including median, min, and max will be reported to the same level of precision as the original observations. If any values are calculated to have more significant digits then the value should be rounded so that it is the same level of precision as the original data.

- Following SAS default rules, the median will be reported as the average of the two middle numbers if the dataset contains even numbers.

- Test statistics including t and z test statistics will be reported to two decimal places.

- P-values will be reported to 3 decimal places if greater than 0.001. If less than 0.001, then p-values will be reported as <0.001. P-values will be reported with a leading 0, e.g., 0.05 rather than .05.

- No preliminary rounding should be performed; rounding should only occur after analysis. To round, consider digit to right of last significant digit: if < 5 then round down, if >=5 then round up.

- All listings will be sorted in order of treatment group (e.g., active, placebo), participant, and time of assessment (e.g., visit, time, and/or event).

If departures from these general conventions are present in specific sections of this SAP, then those conventions will take precedence over these general conventions.

The items listed below will apply to all data analyses.

- For categorical variables, summary statistics will consist of the number and percentage of patients in each category. All percentages will be rounded to one decimal point. The number and percentage of patients will always be presented in the form XX (XX.X) where the percentage is in parentheses. To ensure completeness, all summaries for categorical and discrete variables will include all categories, even if none of the patients had a response in a particular category. Unless otherwise noted, for all percentages, the number of patients in the analysis population for the treatment group who have an observation will be the denominator.

- For continuous variables, summary statistics will consist of the number of patients, mean, median, standard error of the mean (SE), minimum, and maximum values. The summary statistic n will be the number of patients with non-missing values. All means and medians will be reported to one more significant digit than the values being analyzed. Standard errors will be reported to two more significant digits than the values being analyzed. The minimum and maximum will be reported to the same number of significant digits as the values being analyzed.

- For tests of hypothesis of treatment group differences, the associated P-value will be reported.

- Version 9.4 or later of the SAS® software package will be used to produce all summaries, listings, statistical analyses, and graphical presentations.

4. ANALYSIS POPULATIONS

4.1 Intention-to-Treat Population

Section 11.3.1 of the protocol defines the intention-to-treat (ITT) sample as follows:

> Consistent with the primary statistical method that will use a linear mixed model, the intention-to-treat (ITT) sample is defined as all study participants who have at least one valid measurement of the primary endpoint (at Visit 2 or later. A "valid" assessment for the primary endpoint is defined as at least 4 daily reports of pain intensity and duration collected in the 7-day period prior to the visit (Section 2.5.1 of the protocol). Treatment group allocation will be defined as the study arm into which the subject was randomized regardless of the actual study treatment received. By necessity, the ITT sample excludes participants who provide no data for the primary endpoint after randomization. Note that follow-up

assessments will be included in the ITT analysis when sufficient diary data are collected at the follow-up assessment, even if there were protocol violations. Likewise, the ITT sample will include all available follow-up assessments from any discontinued participants.

4.2 Per-Protocol Population

As stated in Section 11.3.2 of the protocol, the per-protocol (PP) sample will be a subset of the ITT sample, excluding any assessments made at or after a visit at which the participant is found to have any major protocol deviation as listed in Section 5.2 below and excluding all assessments for participants whose compliance with study medication is less than 60% over all the study visits.

4.3 Safety Population

Section 11.3.3 of the protocol defines the safety population as follows:

> The safety sample will include all randomized participants who received at least one dose of study medication. Participants in the safety sample will be analyzed with the treatment group according to the medication they actually received, regardless of their randomized assignment.
>
> All adverse events (AEs) will be recorded on a case report form regardless of study population. For safety analyses, all AEs will be provided in listings, but only treatment emergent adverse events (TEAEs) within the safety population will be reported in summary tables.

5. STUDY PATIENTS

5.1 Disposition of Patients

The screened population will consist of all consented participants and the randomized population will consist of all screened participants who are randomized. Any participant who does not meet the criteria for randomization with be discontinued from the trial. Participants who withdraw from the study voluntarily or are withdrawn by an investigator will be discontinued from the study. Study staff will complete a study disposition form in the electronic data capture system, indicating the reason for discontinuation. The disposition of all participants and the study populations to which they belong will be presented in the participant listings and summarized by treatment allocation in a disposition table.

5.2 Protocol Deviations

The protocol deviations listed below are related to the use of investigational product and concomitant therapies and will be considered major protocol deviations. These deviations will exclude a participant's data from the PP analysis (Section 4.2).

- Participant is unable to reach the maintenance dosage of the study drug or placebo or is unable to remain on the maintenance dosage.
- Initiation of opioid medications.
- Initiation of TMJ surgical treatment (including TMJ arthrocentesis).
- Initiation of therapeutic injections for the management of pain.
- Initiation of occlusal appliance therapy.
- Initiation of any of the following non-pharmacological therapies: acupuncture, biofeedback, and/or TENS.
- Initiation after Visit 1 and usage between Visits 1 and 4 of short-acting, non-prescription analgesics (e.g., NSAIDs, aspirin, and/or acetaminophen) for pain that exceeds the definition of episodic use as described in Section 5.7.1 of the protocol.

These and other types of protocol deviations will be reported during the study. The types of protocol deviations will be summarized descriptively for both the Safety and ITT populations by treatment group. A listing of the protocol deviations of individual participants will be included in the participant listings.

6. DEMOGRAPHIC AND OTHER BASELINE CHARACTERISTICS

Demographic and other baseline characteristics will be summarized descriptively for both the ITT and the PP populations. While no formal statistical testing will be done, all demographic and baseline characteristics will be assessed for clinically significant differences between the treatment groups. P-values from these comparisons will be considered descriptive.

To further assess the overall health of the study participants prior to study treatment, any significant medical history will be included in the participant listings.

All prior and concomitant medications will be coded to preferred drug names and therapeutic drug class using the *WHO Drug Dictionary*. Use of concomitant medications will be summarized for each therapeutic drug class and each preferred drug name. Concomitant medications will include medications reported on the Concomitant Medications page of the CRF.

7. MEASUREMENTS OF TREATMENT COMPLIANCE

Participants will be asked to bring their medication containers to each visit during the treatment period. Study staff will count the number of capsules returned and will enter the number on a case report form in the electronic data capture system. Participants will also record the study medication use in their Daily Symptom Diary that will be reviewed by the study staff at Visits 2, 3, 4, and 5. Participant compliance with study medication will be defined as taking 60% or more of the study medication over all study visits. In reports, pill counts will supersede the Daily Symptom Diary in determining compliance. Treatment compliance will be summarized by treatment group and ITT population.

8. EFFICACY EVALUATION

8.1 Handling of Dropouts or Missing Data

There will be no imputation for missing data. Missing data will be accommodated in the linear mixed model.

8.2 Efficacy Variables

The efficacy variables are listed and described in Table 8-1.

Table 8-1 Efficacy Variables

Efficacy Variables	Description and Derivation
**Primary**	
Net Change in the Mean Pain Index	The weekly mean pain index will be computed for the week prior to randomization and for each week during the treatment phase. The weekly mean pain index will be based on at least 4 daily reports of facial pain intensity and duration collected in the 7-day period prior to the visit. When there are fewer than 4 daily reports, the weekly mean pain index will be considered missing. The net change in the mean pain index will be calculated as the weekly mean pain index at V2, V3, or V4 minus the weekly mean pain index at V1.
30% Responder Status	The binomial 30% responder status will be positive if the participant experiences a decrease of 30% from V1 in their TMD weekly mean pain index at V4.
50% Responder Status	The binomial 50% responder status will be positive if the participant experiences a decrease of 50% from V1 in their TMD weekly mean pain index at V4.
**Secondary**	
Net Change in the Mean Pain Intensity	The weekly mean pain intensity will be based on at least 4 daily reports of facial pain intensity collected in the 7-day period prior to the visit. When there are fewer than 4 daily reports, the weekly mean pain intensity will be considered missing. The net change in the mean pain intensity will be calculated as the weekly mean pain intensity at V2, V3, or V4 minus the weekly mean pain intensity at V1.
Net Change in the Mean Pain	The weekly mean pain duration will be based on at least 4

Efficacy Variables	Description and Derivation
Duration	daily reports of facial pain duration collected in the 7-day period prior to the visit. When there are fewer than 4 daily reports, the weekly mean pain duration will be considered missing. The net change in the mean pain duration will be calculated as the weekly mean pain duration at V2, V3, or V4 minus the weekly mean pain duration at V1.
Quantitative sensory tests of sensitivity to experimental pain (QST form)	For each visit when it was measured, Thermal Pain Tolerance, recorded in degrees Celsius, will be computed as the mean of 4 pain tolerance trials.<br>For each visit when it was measured, Thermal Pain Threshold recorded in degrees Celsius, will be computed as the mean of 4 pain threshold trials.
Pressure Pain Threshold (DC/TMD exam)	For each visit when they were measured, five variables, recorded in kg, representing mean Pressure Pain Threshold at each of five anatomical locations: a) Temporalis muscle, b) Masseter muscle; c) TMJ; d) Trapezius muscle; and e) lateral epicondyle eminence (item 8 of DC/TMD Exam form). At each site, the mean response to two trials, each performed bilaterally, will be computed. For each trial, threshold is defined as the amount of pressure at which the participant first perceives the stimulus to be painful..
Facial pain ratings (SF-McGill questionnaire)	For each visit when it was administered, two subscales, each a continuous variable, will be computed from the Short Form McGill Pain Questionnaire: a) Affective component, and b) Sensory component.<br>In addition, 4 variables assessing facial pain will be analyzed: a) Present Pain Intensity (Item II of the SF-McGill); b) Weekly Pain Intensity (Item III of the SF-McGill); c) Weekly Pain Duration (Item IV of the SF-McGill); and d) Weekly Fatigue (Item V of the SF-McGill).
Clinical measures of limitation in jaw function (DC/TMD Exam)	For each visit when they were measured, the following clinical measures will be derived.<br>Three measurements of range of jaw motion will be analyzed, each recorded in millimeters: a) Pain-free jaw opening; b) Maximum unassisted jaw opening; and c) Maximum assisted jaw opening (Item 4 of the DC/TMD form)<br>The count of clinical pain masticatory structure locations will be computed (range=0 to 8) representing locations

Efficacy Variables	Description and Derivation
	where pain is reported during the 30-day period prior to examination: temporalis muscles, masseter muscles, other masticatory muscles, and TMJs, each reported bilaterally (Item 1a of DC/TMD form).<br>The count of examination hyperalgesia masticatory structure locations will be computed (range=0 to 8) representing places in which pain is elicited during jaw provocation during the examination: temporalis muscles, masseter muscles, other masticatory muscles, and TMJs, each reported bilaterally (Items 4, 5 and 6 of DC/TMD form). Jaw provocation includes jaw mobility maneuvers and standardized pressure palpation.<br>The count of examination familiar pain masticatory structure locations will be computed (range=0 to 8) representing places in which pain is elicited during jaw provocation during the examination: temporalis muscles, masseter muscles, other masticatory muscles, and TMJs, each reported bilaterally, and confirmed by the subject to be familiar to pain experienced in the prior 30 days (Items 4, 5 and 6 of DC/TMD form). Jaw provocation includes jaw mobility maneuvers and standardized pressure palpation.
Vital signs	For each visit when it was measured, systolic and diastolic blood pressure (recorded in mmHg) and heart rate (recorded in beats per minute) will be computed, each as the mean of three repeated measurements taken 2 min apart and recorded as Vital Signs in RAVE.
Subjective health status (SF-12v2 questionnaire)	For each visit when it was administered, published scoring algorithms will be used to compute two continuous variables from the SF-12v2 questionnaire: a) Physical Component Subscale and b) Mental Component Subscale (SF-12v2 questionnaire).
Headache Impact (HIT-6 questionnaire)	For each visit when it was administered, published scoring algorithms will be used to compute the Total Score for headache impact, a continuous variable.
Graded chronic pain ratings (GCPS questionnaire)	For each visit when it was administered, published scoring algorithms will be used to compute two continuous measurements (Characteristic Pain Intensity and Pain Interference) and one derived ordinal score (Grade of Chronic Pain) from the GCPS questionnaire.

Efficacy Variables	Description and Derivation
Anxiety and Depression (HADS questionnaire)	For each visit when it was administered, published scoring algorithms will be used to compute two continuous measurements: Anxiety, and Depression scores.
Patient global impression of change (PGIC questionnaire)	The ordinal rating of change, measured at the subject's visits 3 and 4, will be reported as two secondary outcome variables.
Subjective measure of jaw functional limitation (JFLS questionnaire)	For each visit when it was administered, published scoring algorithms will be used to compute four continuous scores of subjective jaw function from the JFLS questionnaire:<br>a) Chewing Limitation b) Opening Limitation; c) Verbal & Emotional Expression Limitation; and d) Combined Global Measure.
Psychological Stress (PSS questionnaire)	For each visit when it was administered, published scoring algorithms will be used to compute a continuous summary score of psychological stress from the PSS questionnaire.
Somatic symptoms (SCL-90R questionnaire)	For each visit when it was administered, published scoring algorithms will be used to compute the Somatization Scale from the SCL-90R questionnaire.
Sleep quality (PSQI questionnaire)	For each visit when it was administered, published scoring algorithms will be used to compute the global measure of sleep quality from the PSQI questionnaire.

8.3 Analysis Methods

The ITT population will be used in all analyses unless otherwise stated. The alpha level for determining significance will be set to 0.05 for all inference tests. The mixed models analyses described below automatically adjust for missing values; there will be no further imputation or adjustments for missing values. During the analysis of the primary hypothesis, treatment allocation will be masked (i.e., groups will be labeled A and B) during the statistical programming and unmasked only after the statistical programming code has been finalized.

8.3.1 Primary Efficacy Analyses

The primary statistical analysis to evaluate the primary hypothesis will use all data from the ITT analytical sample in a linear mixed model. Mixed models will be used to analyze the treatment effect at V4 (Proc Mixed, SAS, version 9.4 or greater, SAS Institute, Inc., Cary, North Carolina). The mixed model will include the net change in the mean pain index as the dependent variable. The model's independent variables will include fixed effects for site, sex, race-ethnicity, the

mean pain index at V1 (as a baseline covariate), visit (V2, V3 and V4), treatment allocation (active versus placebo), and a visit by treatment interaction. Participants will be included as a random effect in the mixed model. The mixed model will be run twice assuming either a compound symmetry or an unstructured variance structure. The mixed model achieving the lowest Akaike information criterion will be selected as the best model and used for reporting of results. The model's denominator degrees of freedom will be adjusted for any possible effects of sample imbalances due to any missing values using Kenward Roger's method in SAS. For descriptive purposes (and regardless of the P-value for efficacy at Visit 4), efficacy estimates at each visit (2, 3 and 4) will be described in terms of adjusted means and 95% confidence intervals.

If there is a treatment effect, then "responder analyses" will be performed to compare the responder status of the treatment allocation groups. Participants will have their binomial responder status classified at both the 30% and 50% response threshold levels. The odds ratios of treatment group allocation on the responder status of participants at both the 30% and 50% threshold levels will be estimated using logistic regression with covariate adjustments for site and V1 weekly mean pain index. The odds ratio for the effect of treatment allocation on responder status for both the 30% and 50% threshold levels will be reported with their 95% confidence limits and their associated P-values.

As secondary efficacy analyses, the above primary hypothesis analyses will be repeated on the PP population. The results of the ITT and PP population based analyses will be reported separately. If it appears reasonable to assume that the results of the primary hypothesis are independent of choice of the analysis population (ITT or PP), then all subsequent analyses will be performed on the ITT population only. Stratified analysis will estimate ITT treatment effects separately for the two sexes and for the main racial-ethnic groups. The analysis will be descriptive, reporting means and 95% confidence intervals. There will be no hypothesis tests of efficacy within demographic subgroups because of the expected small numbers of males and minorities.

8.3.2 Secondary Efficacy Analyses

For secondary efficacy measures that are continuous variables or counts (Table 8-1), the effect of treatment arm on the net change in the secondary efficacy measures will be analyzed using the same statistical model as in the primary efficacy analyses but with derived scores for each measure used as the dependent variables. In summary, data from all applicable visits will be analyzed with linear mixed models as described for the primary hypothesis. For most secondary efficacy measures, the data were collected at V1, V3 and V4, although the schedule differed for a few data forms as shown in Appendix A. Outcomes will be reported at each study visit using number of subjects, the mean ± standard error, and 95% confidence limits of the mean, median, min, and max. Tests for significance of treatment group differences in the change from V1 to V4 will be evaluated with linear mixed models as described for the primary hypothesis. P-value testing for the main effects of treatment allocation will be reported for overall treatment effect and for the treatment effects at each study visit.

For secondary efficacy variables measured on an ordinal scale, the non-parametric signed-rank Wilcoxon test will instead be used to test for change between visits within each treatment group,

and there will be no adjustment for covariates. Patients' Global Impression of Change is the one secondary-efficacy measure that was not measured at V0 or V1, so the test for treatment group differences in that outcome will be evaluated using the Wilcoxon rank sum.

To test the secondary hypothesis of gene-by-treatment interaction, the linear mixed model described for the primary analysis will be extended by adding three sets of predictor variables: (1) the number of *COMT* LPS haplotypes (0, 1, or 2) will be modeled as a fixed effect (both as a continuous variable and as a categorical variable); (2) two-way interactions between haplotype effect and treatment allocation and between haplotype effect and visit sequence; and (3) a three-way interaction term between the haplotype effect, treatment allocation, and visit sequence. The gene-by-treatment interaction at V4 is the interaction of interest. A contrast statement within the mixed model will test for a gene-by-treatment interaction at V4 with a P-value ≤ 0.05 considered statistically significant. Both an unstructured and compound symmetry variance structure, as well as, fitting the haplotype variable as a continuous (linear) and a categorical variable will be tested. The statistical model with the lowest Akaike information criterion will consider the best model and used for inference testing. Residual analysis will be used to confirm that there are no major violations of model assumptions. Model adjusted means will be reported with their 95% confidence intervals. For descriptive purposes, a second set of secondary analyses will be performed on the valine allele of the *COMT* gene using valine allele (Val/Met allele) counts in an identical manner as for the LPS haplotype counts described. A third set of secondary analyses will adapt the approach for analysis of the LPS haplotype, restricting the model to non-Hispanic whites. This is consistent with the approach of some genetic researchers who advocate racial-group restriction as a preferable analytic method to control for population stratification.

The participants' use of rescue medications for TMD pain as well as over-the-counter analgesics (namely, NSAIDs, acetaminophen, and aspirin) for the treatment of any type of pain during a period from V1 to V4 will be summarized by treatment group, both overall and by study visit. The number of days on over-the counter analgesics and the number of days on over-the counter analgesics as a ratio of days on study drug will be summarized (n, mean and standard error) by treatment group, both overall and by study visit. Log-transformed linear regression models will test for treatment group effects on the total number of days a participant is on rescue medications for the treatment of TMD-related pain and (in a separate analysis) for over-the-counter analgesics for the treatment of any pain. The days on rescue medications (or over-the-counter analgesics for any pain) will be log-transformed and used as the dependent variable. If there are some participants with 0 days on rescue medications (or over-the-counter analgesics), then a log(x+1) transformation of days will be used to prevent log(0) values. Study site, mean pain index at V1, and days on study (log-scale) will be used as covariates in the statistical models. Adjusted mean estimates and their 95% confidence intervals of the model adjusted days on rescue medications (or analgesics for any pain) will be reported with their treatment group effect p-values. If residual analysis suggests that model assumptions are unreasonable, then the following alternative model will be used to test for treatment group effects on the use of rescue medications (or over-the-counter analgesics for any pain). This alternative model will be a generalized linear mixed model using a negative binomial link with days on rescue medications (or over-the-counter analgesics for any pain) as the dependent variable and covariates for study site, mean pain index at V1, and for days on study.

There are approximately 20 participants that were enrolled early at UB who did not have the "type of pain" documented when taking over-the-counter analgesics. It cannot be known whether the over-the-counter analgesics were taken due to TMD pain (i.e., rescue medications) and thus these 20 participants will be excluded from the above treatment group effects on the rescue medication use analysis. These subjects will be included in the use of over-the-counter analgesics for any pain.

All of the above secondary efficacy analyses are secondary to and supportive of the primary hypothesis analysis, and thus no statistical corrections will be made for multiplicity for any of the secondary analyses. P-values ≤ 0.05 will be considered statistically significant.

Additional secondary analyses of the incidence of adverse events are described in Section 9.

8.3.3 Exploratory Efficacy Analyses

The mixed model described for the secondary hypothesis of the LPS and Val/Met haplotypes will be modified by specifying genetic variants for the *COMT* promoter, *ADRB2* gene, and *ADRB3* gene. Other genetic variants may also be included in the mixed model. The only difference in these mixed model analyses is that the *ADRB2 and ADRB3* gene types will be included as categorical fixed effects rather than the linear continuous fixed effects in the LPS and Val/Met haplotype mixed models.

Other stratum-specific estimates of treatment effect will be explored in an analogous manner, namely through the use of mixed models in which the stratification variable is a categorical fixed effect. Each stratum will be a mutually exclusive category of study participants, classified according to the following characteristics assessed either at V0 or V1:

- Body Mass Index, computed from measurements recorded at V1, will be used to create two strata, with the dichotomy based on one of two established thresholds ≥25 or ≥30. The decision as to which threshold will be made after inspecting the distribution of BMI, and select the threshold that is closest to the median.

- Smoking status, assessed using the Smoking questionnaire completed at V0, will be used to create two strata: current tobacco smokers or not (the latter therefore including former smokers and never smokers). Smokeless tobacco and other sources of nicotine will not be used for this classification.

- Migraine headache will be classified using responses to the headache questionnaire (HAQ) administered at V1. Two strata will be created, classified according to presence or absence of one or more of four types of migraine sub-types (Migraine without aura, Migraine with aura, Chronic migraine, or Probable migraine without aura).

- Degree of examiner-assessed tenderness to body palpation, recorded at Item 4 of the Fibromyalgia Questionnaire at V0, will be used to create two strata, based as closely as possible on a median split of the total number of tender points.

- Published scoring algorithms will be used to compute the catastrophizing subscale of the revised Coping Strategies Questionnaire (CSQ-R) measured at V1. The two strata will be based as closely as possible on a median split of the subscale score.

- Self-rated general health at V1, reported at item 1 of the SF-12v2 questionnaire, will be used to create two strata based as closely as possible on a median split of the ordinal rating (high, low).

- Anxiety and depression as measured using the two subscales of the Hospital Anxiety and Depression Scale (HADS) administered at V1. Two strata will be created based as closely as possible on a median split of each of the subscale scores (high, low).

- Sleep quality, as measured using the Global Score of the Pittsburgh Sleep Quality Index (PSQI) administered at V1. Two strata will created based as closely as possible on a median split of Global Score (high, low).

- Subjects' rating of confidence in treatment outcome reported at V1 will be used to create two strata based as closely as possible on a median split of the ordinal rating (high, low).

Exploratory analyses of the incidence of adverse events are described in Section 9.

9. SAFETY EVALUATION

9.1 Overview of Safety Analysis Methods

All safety analyses will be carried out using the Safety population. The following assessments will be used to evaluate the safety of propranolol for participants with TMD:

- Adverse events (AEs)
- Vital signs
- Unanticipated Problems

9.2 Adverse Events

All AEs will be coded to a system organ class (SOC) and preferred term (PT) using the *Medical Dictionary for Regulatory Activities* (MedDRA) version 18.0. All tabular AE summaries will be for treatment-emergent AEs (TEAEs). TEAEs will be defined as those AEs with an onset on or after the date of first dose of study drug (study visit 1: V1). If an AE was recorded prior to the first dose of study drug and there was an increase in its severity, the AE will be considered a TEAE. All other AEs will be classified as non-treatment-emergent. Non-treatment-emergent AEs will be flagged in all AE data listings.

An overall summary table will be developed to report the number of events and the incidence of participants having at least one event in the following categories:

- TEAEs

- TEAEs indicated as serious (SAEs)
- TEAEs that lead to study drug discontinuation
- TEAEs with an outcome of death
- TEAEs that were reported as having a definite, probable, or possible relation to study drug
- TEAEs reported as having a severity rating of severe

In addition, a summary table of TEAEs classified by system organ class (SOC) and preferred term (PT) will be provided for each of the following:

- TEAEs
- TEAEs by maximum severity
- TEAEs by relationship to study drug
- TEAEs by week of treatment

The summary of all TEAEs will present both the number of TEAEs and the incidence of TEAEs by severity, and relationship to study drug. When reporting the number of TEAEs, if the same TEAE occurs for a patient on multiple occasions, the event will be counted once for each occurrence. When reporting the incidence, a patient will only be counted once if they ever experience an event within the SOC and ever experience the individual PT.

If for any TEAE (system organ class or preferred term) the incidence rate is 5% or more in either treatment arm then a logistic regression will be performed and descriptive p-values of treatment arm differences will be reported.

For the summary of TEAEs by severity, if the severity of the TEAE is not reported, then the severity of the AE will be counted as severe. If the same TEAE occurs for a patient on multiple occasions, the TEAE will be categorized according to the highest severity rating for that TEAE in that patient. For the summary of TEAEs by relationship to study drug, if the relationship is missing, it will be counted as definite/certain. If the same TEAE occurs for a patient on multiple occasions, the TEAE will be categorized according to the closest relationship to study drug reported for that TEAE in that patient.

9.3 Deaths, Serious Adverse Events, and Other Significant Adverse Events

In addition to these summary tables, the incidence of adverse events leading to death, the incidence of SAEs, and incidence of TEAEs that led to study drug being discontinued will be presented in summary tables and listings. The summary listings will provide all of the information reported for that AE and will include the number of days from date of first dose to date of the occurrence of the AE.

9.4 Vital Signs and Other Observations Related to Safety

Listings will include all participant vital signs and any other comments as provided by the examining clinician. Vital signs statistics will be summarized in table form by treatment group and study visit. These tables will include summary statistics appropriate for variable type as listed in Section 3.

9.5 Unanticipated Problems

Unanticipated problems will be included in the listings.

10. INTERIM ANALYSES AND DATA MONITORING

No interim analyses have been planned.

11. CHANGES TO THE ANALYSES PLANNED IN THE PROTOCOL

No changes to the analyses planned in the protocol have been made.

12. APPENDICES

12.1 Study Schematic

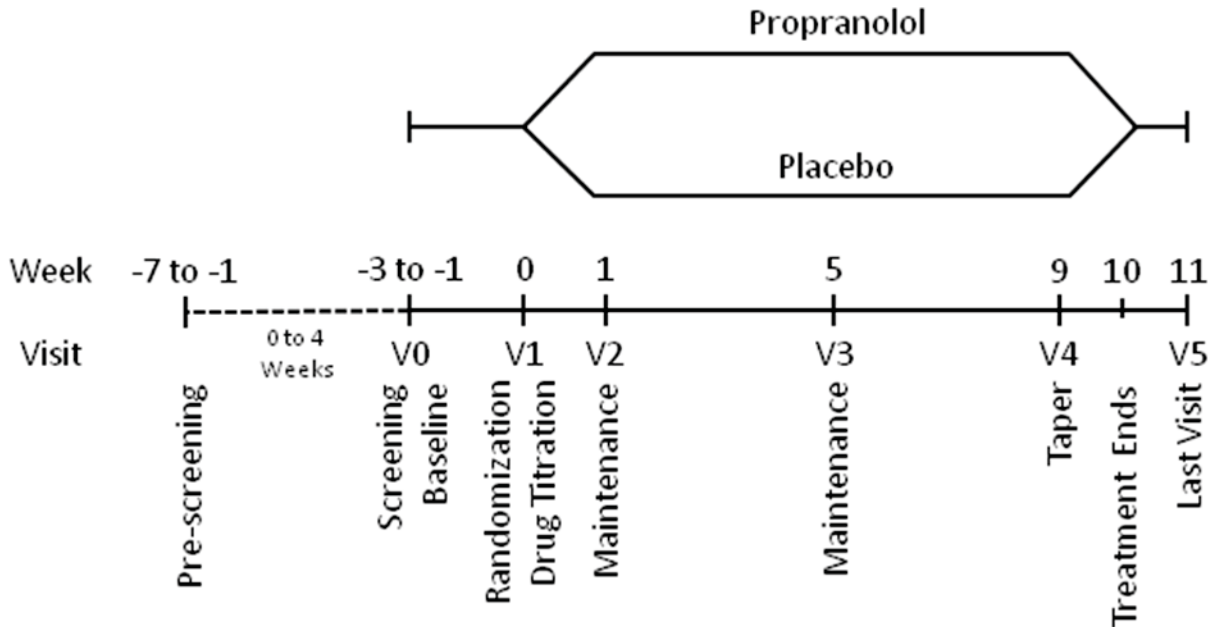

12.2 Schedule of Events

Study Phase:	Pre-screening[a]	Screening and Baseline	Randomization and Treatment (1 week titration, 8 weeks maintenance, and 1 week taper)				Follow-Up
**Clinic Visit:**		**V0**	**V1[b]**	**V2**	**V3**	**V4**	**V5**
**Study Day:**	**≤ 28 days prior to V0**	**7 to 21 days prior to V1**	**0**	**7 (+ 3)**	**35 (± 7)**	**63 (+ 6)**	**77 (+ 7)**
**Procedure**							
Contact Information	x						
Prescreening Interview Script	x						
Informed Consent[c]		x					
Eligibility Review	x	x	x				
AUDIT Questionnaire		x					
Smoking Questionnaire		x					x
Alcohol Consumption per week			x	x	x	x	x
Demographic Information		x					
Medical History and Review	x	x	x	x	x	x	x
Concomitant Medications		x	x	x	x	x	x
Concomitant Therapies		x	x	x	x	x	x
Dispense New Daily Symptom Diaries		x	x	x	x	x	
Adverse Event Review			x	x	x	x	x
Collect Daily Symptom Diaries			x	x	x	x	x
Assess Compliance with Daily Symptom Diaries			x	x	x	x	x
Collect Confidence in Treatment Outcome			x				
Randomization			x				
Dispense Study Drug			x[d]	x	x	x	
Collect Study Drug Container(s)				x	x	x	x
Assess Compliance with Study Drug				x	x	x	x
**Clinical Examinations and Tests**							
Physical Measurements		x					

Study Phase:	Pre-screening[a]	Screening and Baseline	Randomization and Treatment (1 week titration, 8 weeks maintenance, and 1 week taper)				Follow-Up
Clinic Visit:		V0	V1[b]	V2	V3	V4	V5
Study Day:	≤ 28 days prior to V0	7 to 21 days prior to V1	0	7 (+ 3)	35 (± 7)	63 (+ 6)	77 (+ 7)
Vital Signs		x	x	x	x	x	x
Urine Pregnancy Test (females)		x	x	x	x	x	x
Manual Tender Point Exam		x					
TMD Examination		x			x	x	
Heat and Pressure Pain Tests		x			x	x	
12-Lead ECG		x					
Blood Draw			x			x	
Schedule Next Visit	x	x	x	x	x	x	
**Outcome Measure Questionnaires**							
Symptom Inventory		x	x	x	x	x	x
Fibromyalgia Questionnaire		x					
Headache Questionnaire[e]			x				
SF-McGill Pain Questionnaire		x		x	x	x	x
Graded Chronic Pain Scale[e]			x		x	x	
SF-12 Health Survey v2[e]			x		x	x	
Jaw Functional Limitation Scale[e]			x		x	x	
Perceived Stress Scale[e]			x		x	x	
Hospital Anxiety and Depression Scale[e]			x		x	x	
Pittsburgh Sleep Quality Index[e]			x		x	x	
Headache Impact Test (HIT-6) [e]			x		x	x	
SCL-90R Somatization Scale[e]			x		x	x	
Coping Strategies Questionnaire-Revised[e]			x				
Patient Global Impression of Change[e]					x	x	

AUDIT = Alcohol Use Disorders Identification Test; ECG = electrocardiogram; SCL-90 = Symptom Checklist -90R; SF = Short Form; V = Visit

[a] May occur by phone or at a clinic visit, and may be combined with the Screening and Baseline Visit (Visit 0).

[b] If Visit 1 cannot occur within 3 weeks of Visit 0 and the participant is to remain in the study, then Visit 0 will be repeated as an unscheduled visit. The timing of Visits 3-6 and their windows are established from the date of Visit 1.

[c] Includes consent for study participation, consent to store biological specimens for future studies, and Health Insurance Portability and Accountability Act statement, if applicable.

[d] The first dose of study drug should be taken in the evening of the Day 1.

[e] Questionnaires will be distributed at Visit 0 and the participant will return completed questionnaires at Visit 1.
