# Supplementary material for: Effect of Treatment Expectation on Placebo Response and Analgesic Efficacy: A Secondary Aim in a Randomized Clinical Trial
Source: JAMA Netw Open. 2020 Apr 16;3(4):e202907. doi: 10.1001/jamanetworkopen.2020.2907 (PMC7163405; doi:10.1001/jamanetworkopen.2020.2907)
Supplement: Supplement 2. — eFigure. CONSORT Flow Diagram [file jamanetwopen-3-e202907-s002.pdf]

Supplementary Online Content

Sanders AE, Slade GD, Fillingim RB, Ohrbach R, Arbes SJ Jr, Tchivileva IE. Effect of treatment expectation on placebo response and analgesic efficacy: a secondary aim in a randomized clinical trial. *JAMA Network Open*. 2020;3(4):e202907. doi:10.1001/jamanetworkopen.2020.2907

eFigure. CONSORT Flow Diagram

This supplementary material has been provided by the authors to give readers additional information about their work.

© 2020 Sanders AE et al. *JAMA Network Open.*

eFigure. CONSORT Flow Diagram

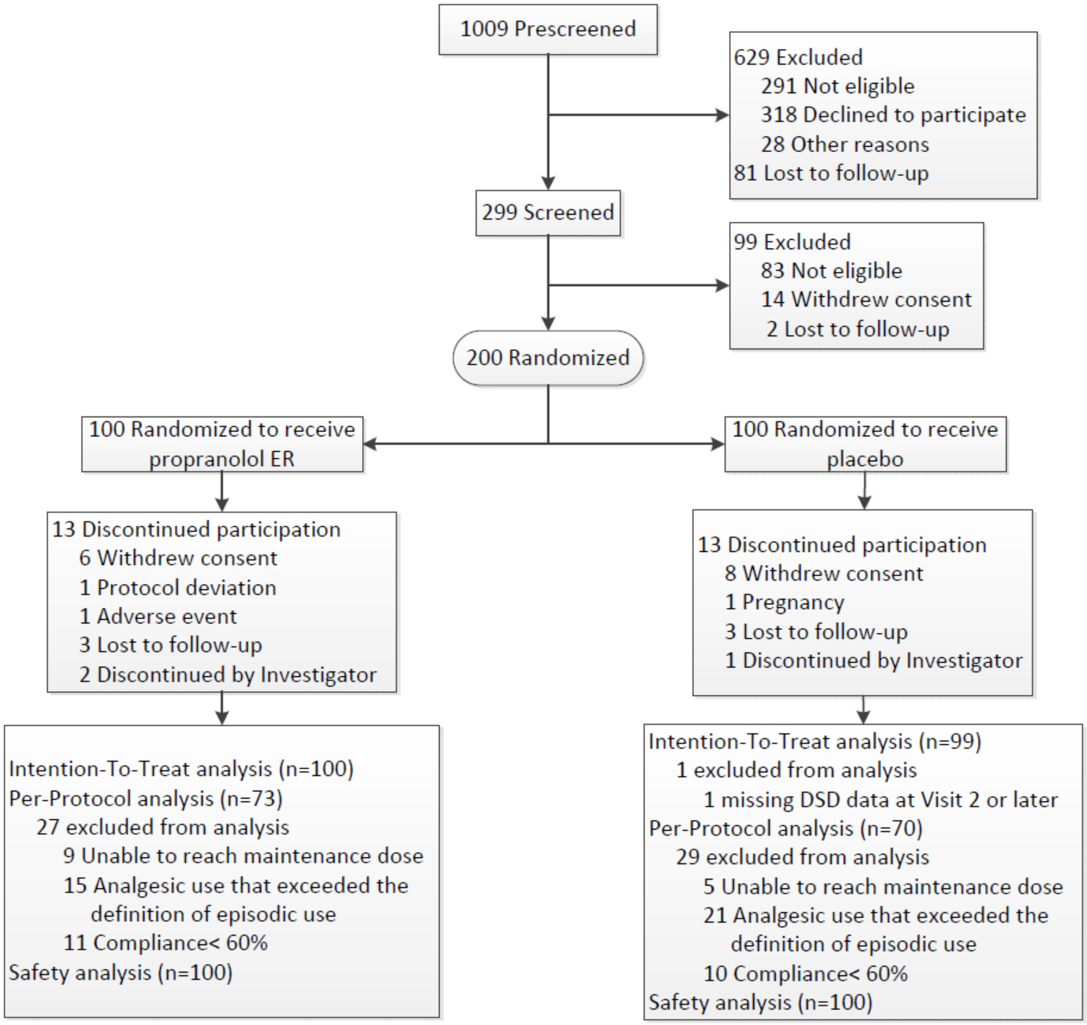

© 2020 Sanders AE et al. *JAMA Network Open.*
