# Supplementary material for: Effect of Treatment Expectation on Placebo Response and Analgesic Efficacy: A Secondary Aim in a Randomized Clinical Trial
Source: JAMA Netw Open. 2020 Apr 16;3(4):e202907. doi: 10.1001/jamanetworkopen.2020.2907 (PMC7163405; doi:10.1001/jamanetworkopen.2020.2907)
Supplement: Supplement 3. — Data Sharing Statement [file jamanetwopen-3-e202907-s003.pdf]

# Data Sharing Statement

Sanders. Effect of Treatment Expectation on Placebo Response and Analgesic Efficacy. *JAMA Netw Open*. Published April 16, 2020. 10.1001/jamanetworkopen.2020.2907

**Data**
**Data available:** Yes
**Data types:** Deidentified participant data, Data dictionary
**How to access data:** Dr. Gary Slade (co-PI) will provide this information. gary_slade@unc.edu
**When available:** beginning date: 01-01-2021

**Supporting Documents**
**Document types:** None

**Additional Information**
**Who can access the data:** Researchers whose proposed use of the data has been approved).
**Types of analyses:** Any approved purpose
**Mechanisms of data availability:** With a signed data access agreement
